# Characterization and gene expression analysis of the *cir *multi-gene family of *plasmodium chabaudi chabaudi *(AS)

**DOI:** 10.1186/1471-2164-13-125

**Published:** 2012-03-29

**Authors:** Jennifer Lawton, Thibaut Brugat, Yam Xue Yan, Adam James Reid, Ulrike Böhme, Thomas Dan Otto, Arnab Pain, Andrew Jackson, Matthew Berriman, Deirdre Cunningham, Peter Preiser, Jean Langhorne

**Affiliations:** 1 Division of Parasitology, MRC National Institute for Medical Research, London, UK; 2 Division of Genomics and Genetics, Nanyang Technological University Singapore, Singapore; 3 Parasite Genomics, Wellcome Trust Sanger Institute, Hinxton, UK; 4 Pathogen Genomics Group, Computational Bioscience Research Center, Chemical Life Sciences and Engineering Division, 4700 King Abdullah University of Science and Technology, Thuwal 23955-6900, Kingdom of Saudi Arabia

## Abstract

**Background:**

The *pir *genes comprise the largest multi-gene family in *Plasmodium*, with members found in *P. vivax, P. knowlesi *and the rodent malaria species. Despite comprising up to 5% of the genome, little is known about the functions of the proteins encoded by *pir *genes. *P. chabaudi *causes chronic infection in mice, which may be due to antigenic variation. In this model, *pir *genes are called *cir*s and may be involved in this mechanism, allowing evasion of host immune responses. In order to fully understand the role(s) of CIR proteins during *P. chabaudi *infection, a detailed characterization of the *cir *gene family was required.

**Results:**

The *cir *repertoire was annotated and a detailed bioinformatic characterization of the encoded CIR proteins was performed. Two major sub-families were identified, which have been named A and B. Members of each sub-family displayed different amino acid motifs, and were thus predicted to have undergone functional divergence. In addition, the expression of the entire *cir *repertoire was analyzed via RNA sequencing and microarray. Up to 40% of the *cir *gene repertoire was expressed in the parasite population during infection, and dominant *cir *transcripts could be identified. In addition, some differences were observed in the pattern of expression between the *cir *subgroups at the peak of *P. chabaudi *infection. Finally, specific *cir *genes were expressed at different time points during asexual blood stages.

**Conclusions:**

In conclusion, the large number of *cir *genes and their expression throughout the intraerythrocytic cycle of development indicates that CIR proteins are likely to be important for parasite survival. In particular, the detection of dominant *cir *transcripts at the peak of *P. chabaudi *infection supports the idea that CIR proteins are expressed, and could perform important functions in the biology of this parasite. Further application of the methodologies described here may allow the elucidation of CIR sub-family A and B protein functions, including their contribution to antigenic variation and immune evasion.

## Background

An important feature of the malaria parasite is the export of proteins to the surface of infected red blood cells (iRBCs). Surface proteins or variant surface antigens (VSA) have been identified so far in three species infecting humans: *Plasmodium falciparum, P. vivax and P. knowlesi*, as well as in the rodent malaria parasites *P. chabaudi *and *P. yoelii *[1-5] . These proteins are implicated in antigenic variation and immune evasion, as well as parasite accumulation or sequestration in host tissues; features which may be critical in determining the outcome of malaria reviewed by [6,7]. Since VSAs are recognized by antibodies, they are also likely to be important targets for a protective immune response eg. [8,9].

In most *Plasmodium *species VSAs are encoded by multi-gene families, usually located in sub-telomeric chromosome regions [2,10-12]. In *P. falciparum*, several VSA gene families have been identified such as *var, rif, stevor *and *surf *reviewed by [7]. Among them, the best characterized is the *var *gene family encoding *Pf*EMP1 proteins, which is implicated in both antigenic variation and sequestration [1,13]. This family consists of 60 genes that can be grouped into several families (A-E) according to chromosomal location, coding and non-intergenic sequences, direction of transcription, and domain arrangements [14,15]. Similarly, the *rif *genes, the largest multi-copy gene family in *P. falciparum *(circa 150-200 genes), has been divided into subgroups A and B on the basis of sequence similarity [16,17] . A- and B-type RIFINs have different sub-cellular localizations, in that only the A-type RIFINs appear to be exported towards the surface of iRBCs [16]. Thus, A-type RIFINs may be more likely to play a role in the host/parasite relationship during the blood stages of *P. falciparum*.

*Plasmodium *interspersed repeat (*pir*) genes have been identified in *Plasmodium vivax *(*vir*), *P. knowlesi *(*kir*), *P. berghei *(*bir*), *P. chabaudi *(*cir*), and *P. yoelii *(*yir*) [2,18,19]. Together, *pirs *form the largest multi-gene family identified to date in *Plasmodium *species, and occupy up to 5% of the parasite genome. On completion of the *P. vivax *Salvador I genome sequence, a total of 12 *vir *sub-families were identified [20-22]. Similarly, 5 sub-families have been identified within the *yir *repertoire [23]. However, so far no function has been ascribed to the subgroups of *yir *and *vir *families.

Microarray studies carried out on *P. vivax *and *P. yoelii *suggest that approximately 50% of the *yir *and *vir *repertoires are expressed in a population of iRBCs during infection, with no evidence of preferential transcription according to their chromosomal location or phylogenetic sub-groups [2,4,20,24]. Surprisingly, considering the large proportion of *yir *genes transcribed in the parasite population, only 1-3 *yirs *are transcribed in a single iRBC [4]. This suggests that transcription of these genes is under tight control. The function(s) of PIR proteins, however, remain unknown.

Rodent malarias offer the only feasible system in which the contribution of PIR proteins to immune evasion and to sequestration and pathology can be examined. Of these, *P. chabaudi *is the only rodent species that naturally produces a chronic infection in mice. These chronic infections are likely to be perpetuated by antigenic variation [25-27]; however, the antigens involved have not yet been determined. *P. chabaudi *also exhibits other important features observed in human *Plasmodium *infections, including rosetting and adhesion to host endothelial cells [5,28], and thus is an ideal model in which to investigate the role of PIR proteins.

The *cir *genes comprise the largest gene family in *P. chabaudi*, and are located in sub-telomeric regions of chromosomes [10,29]. Recently, restriction fragment length polymorphism (RFLP) analysis indicated that *cir *genes may have tissue specific patterns of expression [30]. However, as such techniques are not gene specific, they are only able to provide clues about general *cir *expression. Furthermore, very little is known about the function of CIRs in stimulating or evading host immunity during *P. chabaudi *infection.

Here we describe two major sub-families within the *cir *repertoire of the recently completed *P. chabaudi *AS genome. Using an approach similar to that applied to the RIFIN repertoire [17], we found both conserved and sub-family specific amino acid motifs, and predict functional divergence between the proteins from different CIR sub-families.

We have analyzed in detail the expression of the entire *cir *repertoire via RNA sequencing (Illumina RNA-seq) and microarray. Up to 40% of the *cir *gene repertoire was expressed in the parasite population during infection, and dominant *cir *transcripts could be identified, with some differences in the pattern of expression between the *cir *subgroups. Finally, we found specific *cir *genes were expressed at different time points during asexual blood stages. Together these data will allow future investigation of the CIR family to elucidate their roles in the host/pathogen relationship.

## Methods

### Ethics statement

This study was carried out in strict accordance with the UK Animals (Scientific Procedures) Act 1986 and was approved by the Ethical Committee of the MRC National Institute for Medical Research, and the British Home Office (PPL: 80/2538).

### Annotation of *cir *genes

*cir *genes were annotated using Artemis release 11 [31,32] onto the eight-fold coverage assembly of the *Plasmodium chabaudi chabaudi *AS genome [33]. Putative coding sequences containing conserved features of previously published *cir *genes identified from the three-fold coverage genome assembly [34]. These were then searched for similarity to the *pir *superfamily genes via a combination of basic local alignment search tool (BLAST) and Hidden Markov Model (HMM) on PIR super-family proteins [CIR_BIR_YIR (PF06022)], available in the PFAM database [35], following previously described methods [35,36].

### Detection of conserved motifs

Motif Elicitation analysis (MEME, [37]) was used to identify up to 20 conserved amino acid motifs within the CIR repertoire. The average motif locations were identified and plotted onto each gene. WebLogos were generated with the MEME program, using an adaptation of the WebLogo software [38].

### Analysis of sequence similarity

Amino acid sequences of 183 *cir *genes were aligned using the MUltiple Sequence Comparison by Log- Expectation algorithm (Muscle, [39]). Sequences aligning poorly with the other CIRs were excluded from the alignment and regions containing large insertions were deleted (Additional file 1). The sequence similarity along the alignment is attached in Additional file 2.

Since phylogenetic trees only represent a bifurcating lineage and imply phylogenetic relationships, reticulate networks were created within the program Splitstree 4.0 [40]. All networks used the algorithms NeighborNet [41] for calculation of distances and Equal angle [42] for calculation of splits. 500 bootstrap replicates were generated (Additional file 3).

To support the network, a phylogenetic tree was also constructed using the Maximum Likelihood method from the PhyML server [43]. Here, the evolutionary model applied was Le Gascuel (LG) [44], and the branch support was calculated by approximate likelihood ratio test (aLRT) [45]. Three YIR [46] and three BIR sequences (Ulrike Böhme, WTSI, personal communication) were added to the CIR alignment to allow a root to be placed within this tree (Additional file 4).

Clades identified by both methods with high branch support values contained highly similar CIR sequences. Small clades within each major sub-family were denoted numerically: A1-A5 and B1-B4. Members of each clade are tabulated in Additional file 5.

### Detection of phylogenetic incompatibilities between *cir *genes

Phylogenetic incompatibilities within the alignment of 183 CIRs, and each identified sub-family, were analyzed using the pairwise homoplasy index (PHI) in Splitstree v4.0 [40].

Phylogenetic profiling was used to detect phylogenetic inconsistencies between four *cir *DNA sequences, selected at random, using a hidden Markov model method within the TOPALi platform v2.5 [47,48]. The probability of generating each of the three possible tree topologies for the four sequences was modelled in a given 100 nucleotide window. Possible recombination breakpoints were identified where the most probable topology altered at different positions along the alignment.

Five *cir *quartets, chosen at random, were analyzed per clade by each method described above (Additional file 6).

### Function shift analysis

The alignment of 183 CIRs was split into two files containing only A- and B-type CIRs. The two alignments were then used to apply the 'FunShift' methodology [49], and predict whether these 2 groups of proteins may perform different functions. Positions containing only gaps in a subfamily were not counted.

The method used was the same as described previously by Abhiman and Sonnhammer [49] with a few modifications. Rate-Shifting Sites (RSS) were defined as positions conserved in one sub-family but variable in the other, and were identified using the likelihood ratio test (LRT) program [50]. The U-values generated by this program indicate the likelihood of rate change for each position in the alignment between the two sub-families. U-values above 4.0 were considered significant at the 5% significance level, as previously described [50].

Conservation-Shifting Sites (CSS) were defined as positions that were conserved in both groups, but containing different residues in each. CSS were detected using the method developed by Abhiman and Sonnhammer [49]. This calculates a Z-score based on the normalized cumulative relative entropy at each position of the alignment, between the two sub-families. Z-scores exceeding 0.5 per alignment position were considered significant [49].

CSS and RSS are plotted within a sub-section of the alignment in Additional file 7.

### Mice and parasites

Female BALB/c and C57BL/6 mice aged 6-8 weeks were obtained from the specific pathogen-free unit at the MRC National Institute for Medical Research (NIMR), London. For experimental purposes, mice were housed conventionally with sterile bedding, food and irradiated water on a 12 hour light-dark cycle.

A cloned line of *Plasmodium chabaudi chabaudi *(AS) was used in this study [51]. Stabilates were cryo-preserved in blood from BALB/c mice. To obtain parasites for experimental infection, an aliquot of the stabilate was injected intraperitoneally (i.p.) into immunodeficient BALB/c *RAG2^-/- ^*mice [52]. Blood was taken from the donor mice 7 days after infection and experimental mice were infected by injecting 10^5 ^infected erythrocytes i.p. Parasitaemia was monitored by examination of Giemsa-stained blood films as previously described [53].

Blood was collected from each mouse by cardiac puncture under terminal anaesthesia into Krebs saline (114 mM NaCl, 4.57 mM KCl, 1.15 mM MgSO4) containing 0.2% glucose and 25 U/ml heparin (Leo Pharmaceuticals) 7 days after infection. Leukocytes were removed via Plasmodipur filtration (Euro-Diagnostica) according to manufacturer's instructions. Blood was then stored at -80°C in TRIZOL reagent (Invitrogen) for subsequent RNA extraction.

### RNA extraction

RNA was extracted from *P. chabaudi *infected blood samples by guanidinium thiocyanate-phenol-chloroform extraction according to standard methods [54] and DNase digested using Turbo DNAse (Ambion) according to the manufacturer's instructions.

### Microarray hybridization and analysis

*P. chabaudi *AS is a highly synchronous parasite for which development in the blood follows its host's circadian rhythm. Twelve time-points were then collected; one every two hours, to cover the entire 24 h cycle of blood stage development. At the peak of parasitaemia, one mouse was sacrificed at each time point and thin blood films were made and stained with Giemsa for optical microscopy. The pan-rodent microarray was designed using the OligoRankPick program as previously described [55]. The RNA preparation, Cy-dye coupling to cDNA, hybridization and slide scanning were performed as described by Bozdech and colleagues [56].

Data processing and analysis (including the Fast Fourier Transform) were carried out as described by Bozdech and colleagues [24]. The phaseogram (Figure 7) contains genes with > 1.7 log_2 _ratio of change in mRNA abundance across the IDC. A list of identified genes can be found in Additional file 8.

### RNA sequencing

10 μg of *P. chabaudi *total RNA obtained from BALB/c and C57BL/6 mice was used for this analysis. RNA sequencing was performed using an Illumina GAIIx following the methodology described by Otto and colleagues [57] and was used to create 76 bp paired-end reads. TopHat [58] was used to map reads against the *Plasmodium chabaudi chabaudi *AS reference genome [33], with maximum intron size set to 10000 and inner-mate distance set to 100. Gene expression levels (RPKM) were calculated as defined by Mortazavi and colleagues [59] with minor modifications. Non-uniquely mapping reads were excluded and read-length windows of protein-coding regions that were non-unique were excluded from the gene length term used in calculating RPKM.

Each sequencing run contained different amounts of noise (eg. reads mapping to generally unexpressed parts of the genome: introns and intergenic regions). Therefore we calculated RPKMs over 500 bp windows of exonic and intronic sequences on chromosome 14. For each sequencing run, we took an RPKM cutoff above which only 10% of intron sequences were expressed. A list of identified genes can be found in Additional file 9, with the threshold calculation in Additional file 10. Legends for Additional files 1-10 can be found in Additional file 11.

A Kolmogorov-Smirnov test [60] was used to compare the distribution of *cir *sub-groups according to their level of expression.

## Results

### I) Bioinformatic analysis of the CIR multi-gene family

#### a) Identification of cir genes

Completion of the *P. chabaudi *AS genome sequencing and revised assembly allowed the initial *cir *annotation to be manually revisited. Conserved features from the initially identified *cir *genes [18,29], such as the relative exon lengths, splice sites and amino acid sequences were used to identify putative *cir *coding sequences in the assembled contigs. 117 *cir *genes were first identified and manually annotated. A hidden Markov model (HMM) was then constructed on the basis of the identified *cir *repertoire and was used to detect more divergent *cir *genes, bringing the total of identified *cirs *to 196 [33], including 3 additional *cir*-like genes, containing some but not all expected features of *cir *family members.

Only three partial *cir *genes were identified, arising from the few unresolved contig assemblies for the *P. chabaudi *AS genome. Three long *cir *genes were also identified, containing an extended first exon. The majority of *cir *genes contained one predicted TM domain including some of the divergent *cir*s.

#### b) Sequence similarity of CIR proteins

In order to investigate similarity between CIRs, the amino acid sequences identified during *cir *gene annotation were aligned using Muscle [39], and refined manually (Additional file 1a). Upon addition of more divergent *cir *genes to the repertoire, 16 sequences aligned poorly with the majority of CIRs and were excluded from the analysis (tabulated in Additional file 1b). These included the three partial CIRs, eight CIR sequences with low C- or N-terminal similarity to other members of the repertoire, two CIRs encoded by genes with an atypical structure and three sequences which had been identified as *cir*-like genes, containing some but not all features of *cir *genes.

The sequence similarity of the alignment was determined using Plotcon [61]. The most conservation was found between amino acids 75-120 and 350-385 (Additional file 2).

**Figure 1:**
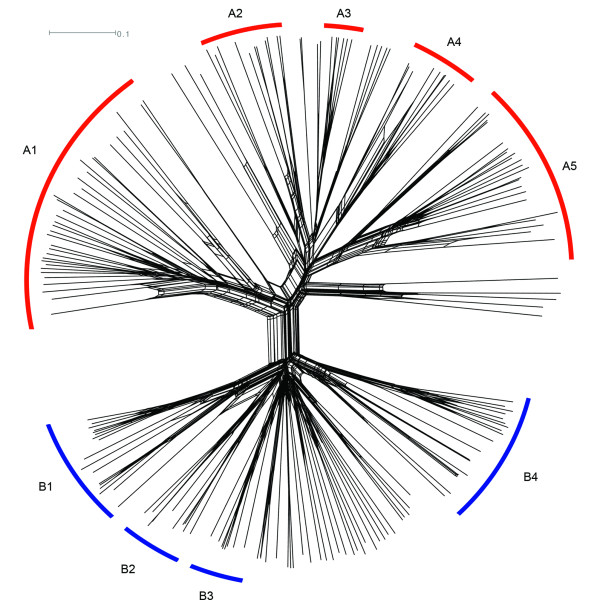
**Relationships between CIR amino acid sequences**. Similarities between CIR sequences were visualized using a network created in Splitstree4 [40], using the NeighborNet and Equal Angle algorithms [41,42]. This network is shown without branch support values, for ease of view. Clades within each major sub-family are indicated by red (sub-family A) or blue (sub-family B) brackets.

To determine the relationships between CIR sequences, a network was created (Figure 1, Additional file 3), using the NeighborNet and Equal Angle algorithms [41,42] in the Splitstree program [40]. This methodology prevented bias from inferred evolutionary relationships, common to phylogenetic analyses [62]. Assumptions of linear evolution are not truly appropriate in the analysis of multi-gene family members which are likely to undergo frequent recombination, as the *var *and *sicavar *genes are known to do [19,63,64]. Instead, production of a network enables visualization of box-like structures (reticulations), where recombination may have occurred between *cir*s and linear evolution cannot be assumed (reviewed by [62]).

Figure 1 shows two distinct sub-families, each of which comprises multiple smaller clades, the members of which are shown in Additional file 5. The large sub-family A contained more divergent CIR sequences, many of which were identified by the CIR HMM. Five clades containing more than five CIR sequences were present within this sub-family: A1-A5 (highlighted in red, Figure 1), of which clade A1 was the most distinct. Sub-family B was comprised of the most conserved CIR sequences, and could also be further sub-divided into 4 clades: B1-B4 (highlighted in blue, Figure 1). The definition of the A1 group as a part of sub-family A was further reinforced by the creation of a phylogenetic tree to support the clades identified by the network in Figure 1. The Maximum Likelihood tree contained the same sub-families and smaller clades as observed in Figure 1, even after three YIR and three BIR sequences ([46], Böhme *et al*., unpublished) were added to the CIR alignment to enable a root to be placed (Additional file 4 TreeBase study accession URL http://purl.org/phylo/treebase/phylows/study/TB2:S12458 [65-67]). The clade A1 continued to cluster within CIR sub-family A, indicating that these CIR sequences were more similar to each other than to members of CIR sub-family B.

In addition, the visualization of CIR sequence relationships using network methodology indicated phylogenetic inconsistencies within the *cir *repertoire, as many box-like structures (reticulations) were present (Figure 1). Across the whole *cir *repertoire, different regions of *cir *genes were found to display different phylogenetic relationships with each other (Additional file 6). Such phylogenetic incompatabilities may arise from either data ambiguity, convergent evolution or recombination events [62], suggesting that recombination may have played a strong role in shaping the *cir *repertoire.

#### c) Identification of conserved amino acid motifs

**Figure 2:**
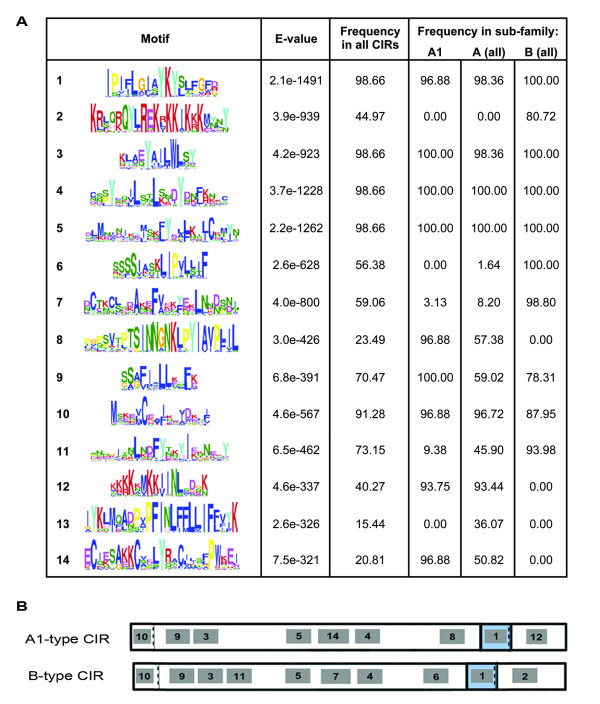
**Conserved amino acid motifs within CIR sequences**. Amino acid motifs present within the CIR repertoire were identified by MEME analysis (Bailey and Elkan, 1994). These are shown as Weblogo images [38], A). Hydrophobic residues are shown in blue, polar, non-charged residues in green, acidic residues in pink and positively charged residues in red. E-values refer to the significance of each motif found within the CIR sequences. The frequency (percentage) of CIR sequences within the whole repertoire, clade A1 or whole sub-families A and B containing each motif is shown. Examples of motif arrangement in CIR proteins are shown, B), using PCHAS_040110 and PCHAS_070130, which belong to the sub-families A (clade A1) and B, respectively. Exon: exon boundaries in the encoding *cir *genes are indicated by dotted lines.

The identification of conserved amino acid sequences may provide indications of possible protein function(s). Multiple Expectation maximization for Motif Elicitation analysis (MEME, [37]) identified 14 motifs in the CIR amino acid sequences. Each motif is represented in Figure 2a as a WebLogo image [38], where the height of each letter indicates the proportion of CIR sequences containing that residue.

Motifs 1 and 3 were the most conserved, being present in 98.66% of CIR sequences. Motif 1 contained the almost unanimous YK residues, corresponding to the start of the third *cir *exon and part of the predicted CIR transmembrane domain. Motif 3 was found within the second *cir *exon and contained the highly conserved sequence YAILWLSY. Motif 10, present in 91.28% of CIRs, contained some degeneracy, but a clear conserved methionine indicated the N-terminus of all CIR proteins. All CIRs possessing motif 10 also contained a cysteine six residues into the sequence. Conserved cysteine residues were also located within motifs 5, 7 and 14. Motif 10 was found at the N-terminus of almost all CIR proteins (91%). Motifs 9, 3, 11, 5, 4 and 1 were also found in members of all sub-families.

The remaining motifs appeared to have sub-family specific distributions, with motifs 2, 6 and 7 only found within sub-family B sequences. By contrast, motifs 8, 12, 13 and 14 were specific to members of CIR sub-family A. The arrangement of motifs within the amino acid sequence is indicated for a typical member of each major sub-family in Figure 2b.

Part of motif 8 (unique to sub-family A) lies within the predicted TM domain and the TM domains of A-type CIRs contain more proline residues than B-type CIRs. In addition the majority of CIRs containing more than one TM domain are found within sub-family A. Together, these differences indicate that the TM domain of CIR sub-type A proteins may have altered properties compared to the rest of the CIRs.

#### d) Similarities between CIRs and RIFINS and function shift analysis

The identification of two major CIR sub-families in Figure 1 resembled the *P. falciparum *RIFIN repertoire organization [16,17]. As the *rif *and *stevor *multi-gene families have been suggested to be distantly related to the *pir *genes [18], we compared the CIR and RIFIN repertoires.

The RIFIN sub-family A is defined by the presence of a 25 amino acid sequence, which is absent from RIFIN-B types [16,17]. An insertion sequence could be detected only in members of CIR sub-family A, which was most conserved in the clade A1. This was located between position 253 and 316 of the alignment of 183 CIRs (Additional file 7a). Furthermore, in this insertion (displayed as a weblogo, [38]), showed some similarities with the A-type RIFIN insertion sequence (Additional file 6b and c, [16,17]). Approximately a third of the residues in each insertion sequence were hydrophobic and very few basic residues were present. Notably, both sequences included two conserved cysteine residues.

**Figure 3:**
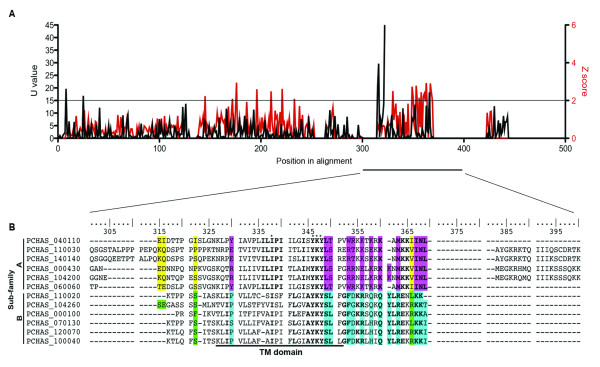
**Funshift analysis of CIR sub-families A and B**. Significant rate shifting sites (RSS, defined as U values greater than 4) and conservation shifting sites (CSS, defined as Z scores greater than 0.5) are plotted along the CIR alignment, A). The sequences of 5 representative CIR sub-family A and B members are shown, B), with the most significantly shifting sites highlighted as follows: U values greater than 15 are shown in yellow for sub-family A and green for sub-family B members; Z scores greater than 2 are shown in pink for sub-family A and blue for sub-family B members. The most significantly shifting sites, (as defined in B), are listed in Table 1.

The two RIFIN sub-families have been shown to display different sub-cellular localizations [16], and thus A- and B-type RIFINs may have different functions. This hypothesis has been supported by bio-informatic analysis of the whole RIFIN repertoire in the *P. falciparum *clones 3D7, DD2 and HB3 [17]. To investigate whether the members of each major CIR sub-type could also have functionally diverged, the alignment of 183 CIRs was split into the two major sub-families, between which Rate- and Conservation- Shifting Sites were compared (RSS and CSS, respectively), as previously described [17,49]. Briefly, RSS measures the probability that each position in the alignment has a different mutation rate in the two sub-families, whilst CSS compares the amino acid distribution between members of each sub-family. Significant CSS and RSS sites are plotted in Figure 3a. 77 RSS (15.4% of all positions) and 158 CSS (31.8% of all positions) were identified along the alignment, strongly suggesting that functional divergence between the CIR sub-families may have occurred according to the criteria generated with protein families of known function [36].

The most significant CSS and RSS sites above the threshold indicated in Figure 3a are highlighted in a section of the CIR amino acid alignment, Figure 3b. Notably, several of the sites had altered conservation of cysteine residues (for example: at site 210 a conserved cysteine residue was present in sub-family A CIRs, but the residues N, K, S, G or D could be found at this position in members of sub-family B, Table 1) or changes in residue conservation within the predicted TM domain, between residues 330-352 (at sites 330, 349 and 350, Figure 3b and Table 1). Similar shifts have also been observed between the RIFIN A and B sub-families, with four highly significant shifts in cysteine conservation, and seven shifted sites detected within the RIFIN TM domain [17].

**Table 1:** Highly significant rate and conservation shifting sites identified between the two major CIR sub-families

Position in alignment	Residues in CIR sub-family:		
		
	A	B	
**Significant conservation shifting sites (CSS):**			**Z score**

145	G W S R H Y I N L Q D	K	2.24
172	I	L	2.09
176	I V L F S	Y	2.95
196	C S W K L F Y I Q	F L I N	2.60
210	C	N K S G D	2.49
221	K	S L F	2.62
233	I A V T S M N Y K H L	C F R Y L	2.09
330	Y E R G V K A I R	P S A L T	2.50
349	L	S	2.74
350	S T K A L V P Y F I	L	2.21
353	W R Q S I G C	F	2.79
354	R T K G A V N	D G H	2.09
356	K E M N T Y	R	2.15
358	K E F T N I	Q H K R N	2.44
360	K	Q	2.21
361	K	Y	2.36
363	M	R	2.93
367	I	K	2.90
368	N	K	2.42
369	L	T A I K V	2.01

**Significant rate shifting sites (RSS):**			**U value**

8	M	M D K T N I L	19.66
25	V N K G A	T Y D V S E K N Q P G I	16.86
315	E K T A S Q P I S D R L Y G N	E P F L T	19.99
316	I Q D E N P A S G H L K V T R	S E D G Q R	29.63
322	I P S V T N A K E Q L C Y H G	S F N T P L	45.64
366	I V L D	L R V I P	18.26

The most significant CSS (Z > 2) and RSS (U > 15) are listed, with the possible amino acids for each of the two sub-families at each position of the alignment. × refers to the IUPAC code, representing any amino acid.

### II) Analysis of *cir *gene expression during *plasmodium chabaudi *infection

#### a) RNA sequencing analysis

**Figure 4:**
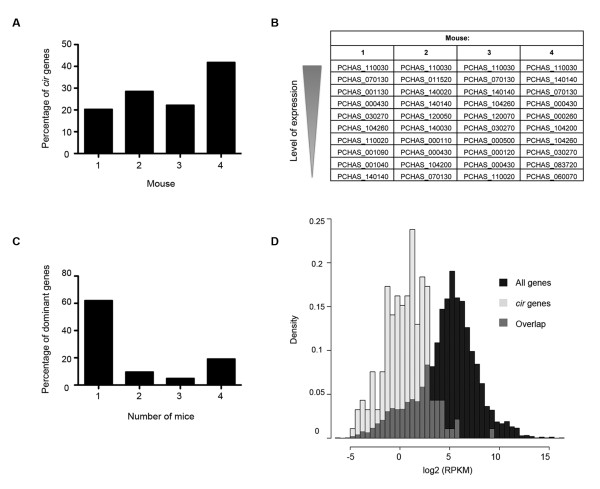
***cir *gene expression during infection**. The percentage of *cir *genes for which transcripts were detected by RNA sequencing is shown for the four *P. chabaudi *infected BALB/c mice, A). The ten dominant *cir*s were tabulated from each infected mouse, ranked according to expression level, B). The number of *P. chabaudi *infected BALB/c mice for which a dominant *cir *transcript was detected is plotted, C). The densities of all *P. chabaudi *transcripts and *cir *transcripts are plotted against the log_2 _RPKM, a measure of transcript abundance, coloured in black and pale grey respectively, D). Regions of overlap are dark grey.

To establish whether the different subgroups of CIR might encode for proteins with different biological functions, we determined the patterns of *cir *gene expression during an infection. For this, mRNA was purified from *Plasmodium chabaudi *AS iRBCs at the late trophozoite stage from four BALB/c mice and sequenced using Illumina/Solexa technology [Array Express accession number: E-ERAD-25 and ENA Study Accession Number: ERP000983]. Results obtained from intergenic regions allowed the determination of a threshold of detection, above which a gene was considered significantly expressed in each sample (Additional file 10). Accordingly, the expression of up to 40% of *cir *genes could be detected in each sample analyzed (Figure 4a).

In addition, mRNA was also sequenced from parasites passaged in two C57BL/6 mice. Both the BALB/c and C57BL/6 inbred strains of mice are commonly used hosts for *P. chabaudi *infection (for example: [68-71]), thus it was important to determine whether either host genetic background could influence *cir *expression. While in the C57BL/6 samples only 17% and 12% of the *cir *repertoire were expressed above background, these lower values were at least partly the result of a higher RPKM cutoff due to relatively high expression of annotated introns and thus probably do not reflect differences in *cir *expression between BALB/c and C57BL/6 mice.

A list of the ten most highly expressed *cir *genes was established for each sample (Figure 4b). As their expression was most highly detected in a population of parasites (either due to higher levels of expression in selected iRBCs or expression in a higher number of iRBCs), these will henceforth be referred to as "dominant *cir*s". When we compared the lists of dominant *cir*s thus established, it was evident that most of these genes (> 60%) were different between the samples analyzed (Figure 4c). This indicates that the relative level of *cir *transcripts may vary from one mouse to another; which could reflect antigenic variation, immune selection of iRBCs or sampling differences upon infection of the mice. Whilst the overall level of *cir *expression was significantly lower than for other parasite genes (Kolmogorov-Smirnov test; D = 0.6742, P-value < 2.2e-16, Figure 4d), the dominant *cir *transcripts were found amongst the most highly expressed parasite genes.

Surprisingly, the same gene (PCHAS_110030) was expressed more than ten fold higher than any other *cir *genes in all the BALB/c samples analyzed. Similarly, three other *cir*s (PCHAS_140140, PCHAS_070130, and PCHAS_000430) were also dominant in these four samples. Furthermore, all of these genes, except PCHAS_140140, were dominant in at least one of the two C57BL/6 mice studied (Additional file 9). This indicates that some *cir *genes are consistently highly represented in a population of *Plasmodium chabaudi AS *parasites at the late trophozoite stage during the peak of parasitemia (8 days post infection).

**Figure 5:**
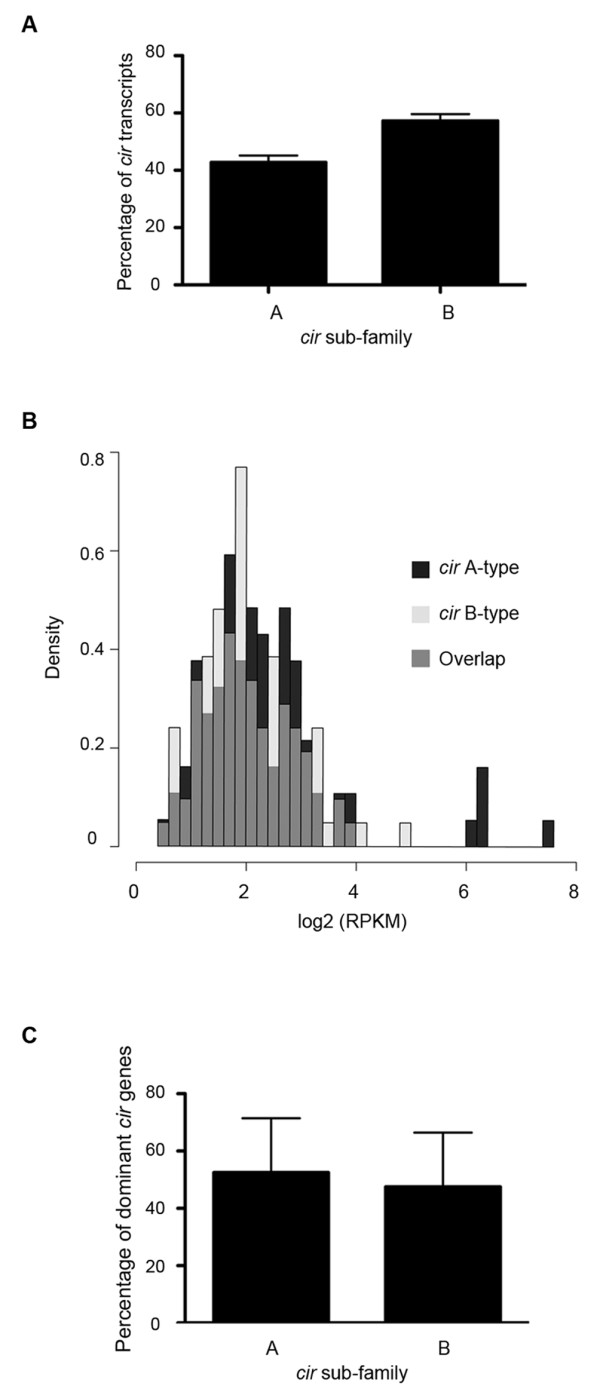
**Comparison of A- and B-type *cir *expression during *P. chabaudi *infection**. The percentage of *cir *transcripts belonging to sub-families A and B is shown, A). The mean of the four BALB/c mice is plotted; error bars represent the standard error of the mean. The density of *cir *transcripts is plotted against the log_2 _RPKM, B). Transcripts belonging to *cir *sub-families A and B are coloured in black and pale grey, respectively. Regions of overlap are coloured dark grey. The percentage of dominant *cir *transcripts belonging to sub-families A and B is shown, C). The mean of the four BALB/c mice is plotted; error bars represent the standard error of the mean.

To evaluate whether there was a relationship between CIR sub-families and functions, a comparison of their expression patterns was undertaken. In our analysis, more of the expressed *cir*s belonged to sub-family B (on average, 57.2% ± 4.7% were B-type *cir*s and 42.3% ± 4.7% were A-types; Figure 5a). A similar result was obtained in both analyzed C57BL/6 mice (58.33% ± 8.41% of *cir*s belonged to sub-family B and 41.66% ± 8.41% were A-type *cir*s, data not shown). When the expression levels of *cir *genes from each sub-family were compared, no statistically significant differences could be detected (K-S test, P = 0.46). It thus seems that the overall level of expression between *cir *genes belonging to sub-families A and B was similar.

However, the most highly expressed *cir*s were all members of sub-family A (Figure 5b). Indeed, PCHAS_110030, the most highly expressed *cir *gene in the six samples analyzed, belonged to this sub-family. We investigated whether this was also the case for the other dominant genes identified previously, Figure 4b. However, in each sample analyzed, about half of the dominant *cir *genes identified belonged to each sub-family (on average, 52.5% ± 18.9% of the dominant genes were A-type *cir*s and 47.5% ± 18.9% were members of sub-family B, Figure 5c, indicated by black dots in Additional file 3). This observation was confirmed in the C57BL/6 mice (on average, 50% ± 14.14% of the dominant *cir*s belonged to sub-family A and 50% ± 14.14% were B-type *cir*s).

#### b) Microarray analysis

Timing of transcription could be an alternative mechanism by which CIRs of different subgroups mediate different functions, therefore we investigated the transcriptional pattern of these genes throughout the intra-erythrocytic developmental cycle (IDC) in *P. chabaudi*. For this purpose parasites were collected at 2-hour intervals from mice with a synchronous infection of *P. chabaudi *for a total of 24 hrs, representing one complete IDC of the parasite. The RNA was extracted and *cir *transcription was determined using the pan-rodent *Plasmodium *spp microarray, which has been described previously [55].

**Figure 6:**
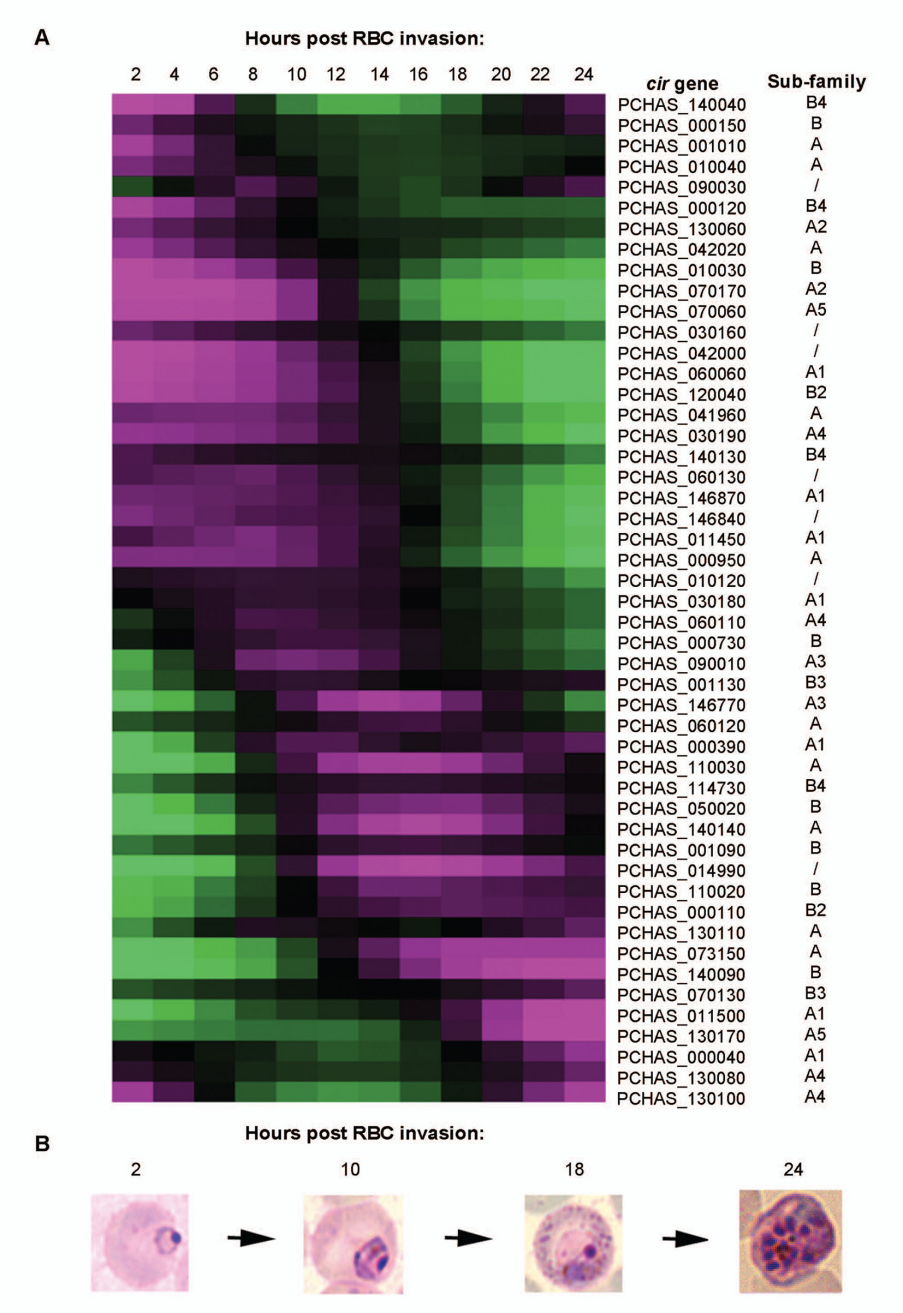
**Transcriptional profile of 49 detected *cir *genes throughout the IDC**. 49 *cir *transcripts could be detected by microarray, which are listed along with sub-family classifications, next to their pattern of expression throughout the IDC, A). Black shading indicates no change in *cir *expression for the iRBC population, whilst magenta and green shading indicate higher and lower levels of *cir *transcripts, respectively. Unclassified *cir *genes are denoted by/. Representative parasite stages from four of the time-points throughout the 24 hr IDC are shown, B).

Figure 6 shows the detected *cir *expression patterns throughout the IDC, where each time-point represents parasites collected from a single mouse [accession number in GEA: GSE33333]. Of the total 96 unique *cir *genes represented on the array, 49 showed clear transcriptional activation throughout the IDC. Each *cir *showed a single peak of transcription in line with previous reports that in *Plasmodium*, genes are only activated once during the IDC [24,56]. Importantly, not all *cir *genes were activated at the same time but rather showed progressive transcriptional activation throughout the IDC with approximately one third of the analyzed *cir*s respectively showing peak transcriptional activity at the ring, trophozoite and schizont stages. Similar observations have also been made in *P. vivax*, where members of the *vir *gene family showed a comparable transcription pattern [24].

Differences in absolute levels of transcription of the two *cir *sub-families could not be quantified using this approach as two-channel hybridization only allows relative abundance comparison for each gene and not between different genes. However, many members of each sub-family could be detected at every time point. These data also strongly suggest that different *cir*s are indeed transcribed at different stages of parasite development in the erythrocyte in line with their products having different functions.

Interestingly, several of the dominant *cir *transcripts from the RNA sequencing experiments were also detected in this microarray timecourse, despite the fact that this material was obtained from separate *P. chabaudi *infections. These *cir*s were: PCHAS_110030, PCHAS_140140, PCHAS_110020, PCHAS_001090, PCHAS_070130, PCHAS_000110 and PCHAS_000120, which comprised approximately a third of the dominant *cir*s identified by RNA sequencing analysis. In addition, as each time point represents the material from a single mouse, the progression of *cir *transcription appeared to be conserved in the mice that were infected with a single starting parasite population and would suggest that limited switching occurs within a single cycle of asexual development. Importantly, the peak timing of expression of these *cirs *detected by microarray is consistent with the parasite stage used for the RNA sequencing analysis, further supporting the notion that the timing of expression of different CIR is conserved in different independent infections.

## Discussion

In total, 196 *cir *genes have been identified and annotated in the *P. chabaudi *genome. The *cir *genes distribute into two major groups, according to sequence similarity. Many *cir*s from both sub-families are transcribed during *P. chabaudi *infection, although dominant *cir *transcripts are present. Differences between the sub-families, such as sub-family specific amino acid motifs and altered residue conservation, particularly within the predicted TM domain, indicate that the two sub-families could be functionally divergent. Recombination of *cir *genes may have occurred within the *cir *repertoire, consistent with that observed in other *Plasmodium *multi-gene families including *var *and *sicavar *(for example: [63,64]).

Similar to the VIR and YIR gene families [2,20,22,23], we confirm that there are also sub-groups within the CIR repertoire in agreement with Ebbinghaus and Krucken [30]. As each of the major groups defined here represented almost half of the CIRs, we defined these as the sub-families: A, comprising the more divergent CIRs; and B, containing more conserved CIRs. These CIR sub-families could each be further sub-divided into smaller clades. Despite the different analysis of CIRs recently described [30], the same groups could be identified within our network (as clade A1 and sub-family B), indicating that the sequences in each of these groups are indeed highly similar to each other. While the overall conservation of PIRs across species is relatively low, some evidence exists for sub-families being shared between *P. yoelii *and *P. berghei *[23], as well as *P. chabaudi, P. yoelii *and *P. berghei *[30] suggesting common functions.

The extensive sequence variation often seen in multi-gene families like *var, rif *and *stevor *in *P. falciparum*, or the *pir*s in *P. vivax *and rodent malaria parasites, is thought to reflect that these genes are under immune pressure (eg. [2,29,72-74]). In our analysis however, we have identified 14 conserved amino acid motifs, suggesting that some parts of the CIR sequences are under constraints important for the biological functions of these proteins. While some motifs were found in the majority of CIRs, motifs unique to each of the two sub-families were also detected. This divergence of conserved amino acid sequence motifs in the different sub-groups presents strong evidence that the different CIR sub-types may indeed carry out distinct but related functions in the parasite, as suggested also for PIR and RIFIN sequences [18]. Since the CIR A sub-family contained the most divergent CIRs, these proteins may be exposed to stronger selective pressure, and it is possible that their diverse nature enables immune evasion *in vivo*.

In line with the functional diversification of CIR subtypes A and B, some CIRs belonging to sub-type A possessed a unique amino acid motif (motif 8) at the beginning of the predicted TM domain. This, in addition to different conservation of proline residues and other amino acids within the predicted TM domains of each sub-family, suggests that CIR sub-family A proteins may have a functionally different TM domain than the B-type CIRs, perhaps altering which membrane the proteins are able to span.

Janssen and colleagues have predicted that *rif *and *stevor *could be ancestral to the *pir *genes due to similarities in terms of size, amino acid motifs and conservation of the first intron [18]. The observations that the CIR repertoire could be separated into two major sub-types namely thanks to an insertion within CIR sub-type A, a section of which contained two highly conserved cysteine residues [similar to RIFIN sub-type A, [16,17]], is strong evidence that the CIR family displays further parallels to the RIFINs. The variation we observed in the number and position of cysteine residues in the two CIR sub-families could significantly impact on the overall protein tertiary structure, and thereby function. As predicted for the A and B type RIFINs [17,49], we found high proportions of rate and conservation shifting sites between the two major CIR sub-families, providing further support for the idea of functional divergence.

Transcriptional differences in both in amount as well as timing can indicate functional differences between related genes. Until now, only Northern blot, degenerate PCR and restriction fragment length polymorphism (RFLP) analyses have been used to give indications of *cir *gene expression during *P. chabaudi *infection [29,30]. Here, we have used both mRNA sequencing as well as microarray studies to obtain a detailed picture of *cir *transcription *in vivo*. The expression of up to 40% of the *cir *genes could be detected during an infection. Different *cir*s were transcribed at different times during the IDC of the parasite, with distinct *cir*s being transcribed in ring, trophozoite and schizont stages. The timing of transcription for a specific *cir *appeared to be conserved from one animal to another (as seen in the microarray experiments) as well as across different experiments (as seen with the good overlap between the genes transcribed in the trophozoite stage as detected by both RNA sequencing and microarray), suggesting that CIRs may carry out distinct ring-, trophozoite-or schizont-specific functions. This is further supported by the observation that *pir *transcription follows a similar pattern in both *P. vivax *and *P. yoelii*, where 59% of the *vir*s and 42% of the *yir*s were expressed respectively [4,24].

Whilst most *cir *genes were transcribed at lower levels than other parasite genes, this most likely reflects differences in the proportion of parasites that actually express a particular *cir*, resulting in populations of iRBCs each expressing different *cir *genes. This would be consistent with observations made for *vir*s, *yir*s and *var*s [1,4,75], that individual iRBCs transcribe only one or a small number of these genes. It is therefore of particular interest that three *cir *genes were represented at relatively high levels in all six samples analyzed by RNA sequencing (PCHAS_110030, PCHAS_070130, and PCHAS_000430), with PCHAS_110030 being detected at a level more than 10 fold higher than any other *cir *in all the samples analyzed. In a classic model of antigenic variation, it would be expected that a single or relatively small number of a gene family would be transcribed in any population, with other members of the gene family being transcribed at much lower levels. This is exactly what we have detected during *P. chabaudi *infection.

While our data suggest that *cir*s belonging to sub-family A are more highly transcribed than those belonging to sub-family B, these differences are relatively small. At this stage there it is not clear whether there is a preference for the transcription of A or B sub-families. What is evident though, is that both A and B sub-family members were transcribed at the peak of a blood-stage *P. chabaudi *infection. Considering that the sequence analysis carried out here strongly suggests functional differences between the two sub-types, the expression of both sub-families may indicate that A and B-type CIRs provide complementary but non-overlapping roles at different stages during infection.

## Conclusions

We have here presented a thorough analysis of the *cir *repertoire, highlighting similarities between these genes and other multi-gene families. The two CIR sub-families have been predicted to have different functions, as shown for the RIFINs of *P. falciparum*. Whilst these function(s) remain unknown, the large number of *cir *genes and their expression throughout the IDC indicates that CIR proteins are likely to play key roles in the biology of the parasite. These may include antigenic variation and immune evasion. Our application of RNA sequencing during *P. chabaudi *infection has enabled the detection of dominant *cir *transcripts for the first time, supporting such roles. Further application of these methodologies may elucidate the functions of CIR proteins and help to clarify the roles that members of the CIR sub-families play during infection.

## Competing interests

The authors declare that they have no competing interests.

## Authors' contributions

JL^(1st author) ^carried out the *cir *gene annotation in collaboration with UB, AP and MB; carried out the bioinformatic analyses, supervised by AJ; prepared RNA samples for RNAseq and drafted the manuscript in collaboration with TB. RNAseq data were analyzed by AJR, TDO and TB. Microarray analyses were performed by YXY and PP. JL^(last author) ^conceived of and supervised the study, and edited the manuscript with DC, AJ, AJR, AP, MB and PP. All authors read and approved the final manuscript.

## Supplementary Material

Additional file 1 **Alignment of 183 CIR sequences**. Click here for file

Additional file 2 **CIR alignment conservation**. Click here for file

Additional file 3 **CIR network showing bootstrap values**. Click here for file

Additional file 4 **Maximum likelihood tree of CIR sequences**. Click here for file

Additional file 5 **Sub-families identified from the alignment of 183 CIRs**. Click here for file

Additional file 6 **Detection of phylogenetic incompatibilities between *cir *genes**. Click here for file

Additional file 7 **Identification of similarities between the CIR and RIFIN repertoires**. Click here for file

Additional file 8 **Raw microarray data**. Click here for file

Additional file 9 **Raw RNA sequencing data**. Click here for file

Additional file 10 ***cir *gene expression threshold of detection determination**. Click here for file

Additional file 11 **Legends to Additional Files 1-10**. Click here for file
