# Supplementary material for: Characterization and gene expression analysis of the cir multi-gene family of plasmodium chabaudi chabaudi (AS)
Source: BMC Genomics. 2012 Mar 29;13:125. doi: 10.1186/1471-2164-13-125 (PMC3384456; doi:10.1186/1471-2164-13-125)
Supplement: Additional file 1 — Alignment of 183 CIR sequences. [file 1471-2164-13-125-S1.PDF]

Supplementary data 1 (CIR alignment)

a) CIR amino acid sequence alignment:

```
               ....|....| ....|....| ....|....| ....|....| ....|....| ....|....| ....|....| ....|....| ....|....| ....|....|
                       5         15         25         35         45         55         65         75         85         95
PCHAS_000950   -------MAL NLCNALKDI- EEFLPNS--L SSESKDVNRN LYKTYCPIDQ RGKQECRTDG ERIGAMFKLL LQQL-FVNNE GDLELENKND EYSEYAILWL
PCHAS_001010   -------MTL NLCSAFKGI- GELLPDS--L SPESDDDNHS LYKIYCPDSK TGNPECKTDG QRISAMFRYL LEYL-FVNND DDLKSENQNN EYSEYAILWL
PCHAS_140090   -------MNN -LCGLINVI- DTITSVN-VN NSILFIGNNE TIKECCTNDT GKKEECDSYI KITNSAIIAV LNYF--KNIS VADGDDLEDD KIAEYAILWL
PCHAS_000730   -------MSE NLCKLINSI- DKSITVN-VN NSLAFIEFDN ILTEYCTEEG GGKKECFSYE VLISSIFIKL LKIF------ -MNLDDLEND KHAQYAILWF
PCHAS_070160   -------MNN -LCTIFKGI- DDILVVK-SN NPDYYRHSFS SLNSYCPSSD GKNQECNNYE EITGSAFISL LIYL------ ---KNYVEND KLSQYAILWL
PCHAS_030020   -------MSK KLCGLIIGI- DNGIIVK-LN GSNVQIKNDT TFNDYCPEGM KTNGECDSNI QNVNFAIIKL LTYF-----K SVVDDILEDN KLSEYAILWL
PCHAS_000360   -------MSK KLCGLINVI- DHGVKLT-MN GTNVQIKNDA TFNDYCPEGM KTNGECDSNI QNVNSAIIKL LTYF-----K SVVDDILEDN KLSEYAILWL
PCHAS_110020   -------MDN -LCEAIDQI- DEKITLD-TN GSKQLFHYNT GSYPYCINGE GGTQECFSYE VIVSSLFIKM VNHF------ -MKGDDLKNN KLAEYAILWL
PCHAS_050060   -------MDN -LCKLVNAI- DKGFSVD-VN NLKAIIEFDN IQNQYCT-GD NGGGECFPYE AIVSSYFIKM LNYF------ -MNDKDLNMK KFGEYVILSL
PCHAS_010030   -------MSK EVCSIINTI- DDKVSVN-AN GLIAMVDFNE IHSKYCD--S EEGGFCYSYE AIVSSVFTKI LKHF----ES CNDDDNLENA KLAQYAILWL
PCHAS_030030   -------MDN -LCKVINRL- DRKITVK-VD NLIAMIEFDK IQSKYCT-AK DEEGTCYSYE AIISSYFITL LNHF-----K SCNDNNLEND KLAQYAVLWL
PCHAS_000350   -------MSE KLCKLFSAI- DNGVTVT-VN NSKAMIKFDN IFTEYCYEED GGKKECFSYE VLVTSYFIGL LNYF-----M KVDDLK--HN KNAEYAILWL
PCHAS_011530   -------MDT -LCQLFKGF- DENLVVK-PN NSEFDSDNFP SFNNFCPTDG GKKQKCNSYE EMVISAFLTL IINF----AS INDGGNIEND KIAQYAILWL
PCHAS_073160   ---------- --CEFINTI- DVLLDVK-WD GSKISFKYNS SLDNYCSMGD EKQEDCNNYE EMISSAFLTL LHHF---KSV NAGDENLEDD KLAQYALLWL
PCHAS_070090   -------MLK DLCGAIDKI- DTKISLD-AN DPNLNSDDYM ALNKYCPVNN GNEQKCKNYE EIIISAFMTF LVSF----IS SNGNEYLDRD NFAEYTILWL
PCHAS_000580   -MAESSYDIE KVCEAINKI- DSHLSVN-PD DSTLNSEDFP PLINYCPVNN DSEHNCKNYE ELVSSAFITM LVSF--ISSN HGEYLD--RD KFAEYAILWL
PCHAS_140130   -------MTE DMCRSFISVW TDFPDQL--D GGNYKFSDDG YCEALFT--- --DNKCNTDF DKINAVCFWL FKKI-MWDSS SSSIKEKINT DIIHYIMTWL
PCHAS_140040   -------MTE NMCRGFKTIW DEFPNSV-GK DGDNQSNINN YCETLFN--- ---KKIYDDI DIVNAVSFWL FEQN-FGGGS SLTKNVNSNT NIFHYIMIWL
PCHAS_000740   -------MAD FMCRMFYNVW DDFPDQL--D NGNYKFNDDD YFDNLFT--- --DKNYGNDI DKVNAISFWL FEKN-LWDSS SSSINAKSNT DIVHYIVIWL
PCHAS_100030   -------MAE NMCRSFYNVW TDFLDQL--N EGDYKFNDDE YSTNLFT--- ---NKSDNDI DKVNAVSFWL FEQN-LWDCS SSSINAKSNT DIVYYIVIWL
PCHAS_001120   -------MAE LMCQRFNNVW TDFLDQL--D EGKYKFTDDG YSDNLFT--- ---NKSDDDL NKINAVSFWL FEQN-LWDCS SSSINEKRNT DIVYYIVIWL
PCHAS_011520   -------MTE NMCRRFKTVW GDFPDQL--D NGNYNFKDDT YFKDFFT--- --NKSCDNDI DKVNAISFWL FIQN-FGDGS SLTKDVNSNT NIFHYIMIWL
PCHAS_070030   -------MAE LMCQRFNNVW NDFPDQL--D NGNYKFNDDE YFKNFFT--- --NKCCDNDI DKVNAISFWL FKQN-LWDSS SSSINAKSNT DIVHYIVIWL
PCHAS_073190   -------MAE NMCRRFKTVW GDFPDQL--D NGNYNFKDDT YFKDFFT--- --NKSCDNDI DKVNAISFWL FKQN-FGDSS SSSINAKSNT DIVYYIVIWL
PCHAS_000570   -------MAE LMCRKFNNVW TDFPESV-GE DGNCQSITSD YWETLFN--- --NKKCGNYI DQVNAVSFWL LVQN-FGDGS KFTKNVISDT NIFHYIMIWL
PCHAS_114730   -------MAE NMCQRFKTVW DDFPEGV-GE DGNSQIKIKE YWDILFK--- --NKNYDNYI DKVNAVSFWL FVQN-FGDNF SFTKNVDSNT NIFHYIMIWL
PCHAS_010020   -------MSE NMCKYFKLVW DDFPDTV--R DGKYQFNDHN IYSTYCD--- NNTDKCQTDI DKINAGYLSF FKTL-FGDSE SIKNHEKNNM NISQYIIIWL
PCHAS_000280   -------MNE DMCKIFEMMW EDFPDTL-GN DGNYQFKNED IYSKYCD--- SNCDNCRTDL DKINAACFVL FKIF-FEDSE SFMKNAKCNM NIAQYIIIWL
PCHAS_000490   -------MNE DMCKIFEMMW EDFPDTL-GN DGNYQFKNED IYSKYCD--- SNCDNCRTDL DKINAACFVL FKIF-FEDSE SFMKNAKCNM NIAQYIIIWL
PCHAS_000030   -------MNE DMCKFFEVVW TDFPDTL-DD DGNYQFKSDD TYQTYCI--- --NETCNNDI DKINAWCLCL LNLI-FGNFE SFTGYAKSNM NNVQYILAWL
PCHAS_000090   -------MNE DMCKFFEVVW TDFPDTL-DD DGNYQFKSDD TYQTYCI--- --NETCNNDI DKINAWCLCL LNLI-FGNFE SFTGYAKSNM NNVQYILAWL
PCHAS_000270   -------MNE DMCKFFEVVW TDFPDTL-DD DGNYQFKSDD TYQTYCI--- --NETCNNDI DKINAWCLCL LNLI-FGNFE SFTGYAKSNM NNVQYILAWL
PCHAS_000120   -------MNE DMCKFFEVVW TDFPDTL-DD DGNYQFKSDD TYQTYCI--- --NETCNSDI DKINAWCLCL LNLI-FGSFE SFTDHAKSNM NNVQYILAWL
PCHAS_001090   -------MFM NLCDAINGI- DDLIKAE-VK GEEIYIENNR ILNANCPYIT EEGGQCIGYI AAVISAFIGM LKTF-----E NSDIDVSENG EFAQYAILWL
PCHAS_011510   -MAQSNHTIK DVYDDIFKI- NDYFYET-EQ GQLMLDKTHG SIEDYCYGNP PEKGNCNDYL KMASSGVIHL LKNY------ --KKYGLGHD KLAEYAILFL
PCHAS_070050   -MTNTSDNLK DVYRKFATI- DDYFYVT-DD GQVRVDTQHK SIIQYCNKNN SVTYDCNDYF QLVSCGFIYL LKTL------ -KDNYKLEDD KLAEYVILWL
PCHAS_000720   -MTNPSYDIE KVYRDIYAI- EDYFYVT-ND DQIRVDRKHK SIIQYCNKNN SVTYDCNDYF QLVSCGFIYL LKTL------ -KDNYKLEDD KLAEYVILWL
PCHAS_070020   -MENSSYDIG DVYRDIYAI- EDYFYVM-DD GEIRVDIAHE SILQYCNKNN SVTYDCNDYF QLTSCGFIYL LKTL------ -KDNYKLEDD KLAEYVILWL
PCHAS_104260   -MENSSYDIG DVYRDIYAI- EDYFYVM-DD GQVRVDRQHQ SIIQYCNKNN SGPYNCNDYF QLTSCGFIYL LKTL------ -KDNYKLEDD KLAEYVILWL
PCHAS_140020   -MENSSYDIG DVYRDIYAI- EDYFYVM-DD GEFRVDRQHE SILKYCNKNT SGTYKCNDYI EMTSCSVIYL LKTL------ -KDNYKLEDD KLAEYVILWL
PCHAS_030060   -MAESSYKIE DVYSTIKTI- SNYFYED-EK DGTKTQVTNE EIHNYCDEDR SDSNKCLDYF EMTSSGVIHL LKDL------ -KKKCNLDDD KLAEYAILWL
PCHAS_000750   -MTESSDNLK DLYSAIKTI- SNYFEGGNNG QLTVNKKYAD LIDKYCDGDK SEKGNCNEHF KIISSGVINL LDNL------ --KKYKLEDD KLADYAILWL
```

Supplementary data 1 (CIR alignment)

PCHAS_000680	-MTKSSYDIE	K-YKEFVTI-	NSYFGEEKKQ	NGEISLKCKE	EIHNYCHGNT	SEKGNCNEYF	KMTSSGVIYL	LENL------	--KKYGLDDD	KLADYAILWL
PCHAS_000420	-MRNPSYKIE	DVYKEFVTI-	SNYFDED-KN	DGTKTKVTNE	AIHNYCHKDR	SDSNKCFDYY	EMTSSGVIYL	LENL------	-KKKCNLDDD	KLAEYAILWL
PCHAS_000100	-MRNPSYKIE	DVYNDIFKI-	SNYFDED-EN	DGTKTKVTNK	AIHNYCHDDR	QEKDKCNDYY	EMTSSGVIHL	INNL------	-KGKNVLDYD	KLAEYAILWL
PCHAS_000310	-MRNPSYKIE	DVYKEFATI-	DGYFYVD-ED	DGSITQVTNK	AIHNYCDDRQ	ENGDKCSGYY	EMTSSGVIYL	LENL------	-KKKCNLDDD	KLAEYAILWL
PCHAS_040040	-MPNPSYNIE	DVYKEFATI-	DGYFYVD-ED	DGSKTKVNNQ	AIHNYCDDRQ	EKDKCCSGYY	EMTSSGVIYL	LENL------	-KKKCNLDDD	KLAEYAILWL
PCHAS_120060	-MRNPSYKIE	DVYKEFVTI-	SNYFNED-EN	DGAKTKVINE	AIHNYCYDKQ	LEKDKCSGYY	EMTSSGVIHL	INNL------	-KGKNVLDYD	KLAEYAILWL
PCHAS_104230	-MRNPSYKIE	DVYKEFATI-	DGYFYVD-ED	DGAKTKVINK	AIHNYCDDKK	REKDKCSGYY	EMTSSGVIHL	INNL------	-KDKNVLDYD	KLAEYAILWL
PCHAS_030040	-MRNPSYKIE	DVYKEFVTI-	NGYFDDD-ED	DGTQTKVNNE	AIHNYCDDKP	KNNDKCSGYY	EMISSGVIYL	LENL------	-KTKCNLDDD	KLAEYAILWL
PCHAS_000340	-MPNPSYNIE	DVYKEFATI-	DGYFYVD-QD	DGSITHVTNK	AIHNYCDDRS	RN-CKCCGYY	EMTSSGVIYL	LENL-----K	KKCNLD--DD	KLAEYAILWL
PCHAS_001110	-------MSE	EVCEAFKKV-	DDCLQIG-MV	STGDICYIDE	VLNEYCPNIE	GQNGKCDTNN	EKISAGFIWL	LITF--EDLC	EGQCSDNENE	KYAEYAILWL
PCHAS_120040	-------MSE	EVYKEIKKV-	DDSLPIG-II	STGGSSSTDR	VLIDYCP-KK	NGERQCETIC	DKISAGFIWL	LISF--ENLC	EGKCSDDENE	KYAEYGILWL
PCHAS_000130	-MEESSDNLK	DVCNFFNKIV	DDCLPTG-II	STGVSCSNDS	VLNEYCPKRE	GGNRQCDTNN	DKISAGFIWL	LISF--ENLC	DSQCSDDENE	KYAEYGILWL
PCHAS_000400	MAESSYKIED	VVCEAFKKV-	DDCLQIG-MK	STGGSCSIDY	AFTDYCPKIE	GQNVNCEANN	EKMSAGFIWL	LITF--ENLC	VGQCSDNENE	KYAEYAILWL
PCHAS_040020	-MAESSYDIE	KVCEAFKKV-	DDCLQIG-MK	STGGSCSIDY	AFTDYCPKIE	GQNVNCEANN	EKMSAGFIWL	LITF--ENLC	VGQCSDNENE	KYAEYAILWL
PCHAS_000110	-------MSK	EVCEEINIM-	GKYIVVK-QK	DSGVDIEFDP	KLNDYCPKNN	GINEQCETND	EKISAGFIWL	LVMF--EHLC	DEECPQNEKD	RYVEYAILWL
PCHAS_120050	-MENSSDNLK	YVCKDIKKI-	DDCLQID--T	IYTGVCSDDT	LYSDYCPMKN	GKKGQCETNN	DKISAGFIWL	LVMF-EHICD	DDECSQNEKD	QYAGYAILWL
PCHAS_104250	-------MFQ	EVCEEINNM-	GKYIVVK-QE	GSGVNIEFDN	YLSDYCPKNN	GRNGQCETND	EKITAGFIWL	VVMF--EYLC	EEKYPQNEKY	QYVEYAILWL
PCHAS_000320	-------MSK	GVCEEINNM-	GKYIVVK-QE	GSGVNIEFDN	KLNDYCPKNN	GRNGQCETND	EKISAGFIWL	LVMF--EHWC	DEECPQNEKD	RYVEYAILWL
PCHAS_000410	-------MSK	GVCEEINNM-	GKYIVVK-QK	DSGVDIEFDN	ILNDYCPKNN	GRNGQCETND	EKISAGFIWL	LVMF--EHWC	DEECPQNEKD	RYVEYAILWL
PCHAS_140030	-------MNE	NMCRILEGVW	GDFPDTL-DG	AGNYQFKNDE	INATYCK---	DNIENCKTDL	DKINVGCFVL	FEIF-FKDSD	SLMENAKNNI	NIAGYILAWL
PCHAS_114740	--MGLMYLCK	YLCKILSFIW	EEFPDTL-GD	DGNYQLKSDG	AYKTFCI---	--NETCDSDL	DKINAWVLSL	FEIF-FKNSD	SLMESAKSNI	NIAAYILAWL
PCHAS_040030	-------MIK	EVCGTISII-	DKFIWVE-QK	GVVEVIQYYN	LVNAYCPKST	PKKNECHSYD	EMISSAVLFF	LKWL---ETS	YDYKDDLKND	KLAEYAILWL
PCHAS_070130	-------MAS	AVCNAIKAI-	DNFIVVK-EG	NIGVNISFKE	ILNPYCTKSL	VNKEECQSYN	EMVSSAFILL	LKFL---NLV	DVYDGDLTND	RLAEYAILWL
PCHAS_073180	-------MAF	GVCGAIKAI-	DKFFSVK-QK	NSVDVIEHYN	LINTYCPKNN	PRKNECHSYE	EMVSSAVIYL	LKYF---EFD	YDYKDDFKND	KIAEYAILWL
PCHAS_000300	-------MAS	GVCGAINGI-	ERLIDVK-EE	DSRVSFKCNV	VLDAYCPKST	SRKNECHSYA	EMVSSSVLFL	LKSL---ENN	YHYEDDLKNA	KLAEYAILWL
PCHAS_120070	-------MAS	GVCNAIKAI-	EKFIVVK-EE	DSGVYFTPNH	ALKAYCNAKE	GGTGVCFSYA	EMVSSSVLFL	LKSL---ETS	YDYGDYLKND	KLAEYAILWL
PCHAS_001130	-------MAY	GVCNAIKAI-	EKLITVK-EE	DSRVYFTPNH	ALKAYCNVKE	DGIGVCFSYA	EMVSSAVLFF	LKWL---ETS	YDYGDYLKND	KLAEYAILWL
PCHAS_100040	-------MAS	AVCGAINTI-	ERLIDVK-EK	DSRVYFTPNH	ALKAYCNVKE	RGTGVCFSYA	EIVSSAVLFL	LKLL---EID	YHYEDDLKNA	KLAEYAILWL
PCHAS_000560	-------MAY	GVCGAINVI-	EKLIVVN-EK	DLGVYFSPNQ	LLKAYCTRVN	DQTGICFSYA	EIVSSAVLFL	LKLL---EID	YHYEDDLKNA	KLAEYAILWL
PCHAS_050020	-------MNK	DLCDVIKGI-	DDLIEVK-RN	TEAIEIIRST	LFDTYCPRKD	GQEGHCIGYS	ETVISAFIHL	QETL----KI	NYSPEELERD	KLAQYAILWL
PCHAS_000020	-------MNK	DLCDVIKGI-	DDLIEVE-VK	AEGIETIRDE	LFNTYCPRQQ	GQDGHCIGYS	ETVISAFIYL	QETL----KS	NYSPEELESD	KLAQYAILWL
PCHAS_000290	-------MNK	DLCDVIKGI-	DDLIEVE-VK	AEGIETIRDE	LFNTYCPRQQ	GQDGYCIGYS	ETVISAFIHL	QETL----KN	NDSQKKLDRD	KLAQYAILWL
PCHAS_060020	-------MSK	EVCKAFKDI-	DKLIKVE-SS	GEDVFIENNK	ILNAYCPYVN	GEGGQCIGYI	EIVNSGFISL	LEKF----NN	FDNGRNLESE	RRFQYAILWL
PCHAS_001040	-------MSK	EGCTYINFV-	NKLIKSN-TT	GTEGKIENHK	SLNTNCP---	--NEKCDTDS	QKLSSAFILL	IKFF------	-SIDDNLEND	KHAEYAILWL
PCHAS_114750	-------MSN	EVCMYINKV-	DKLIKSN-AA	GTEGKIENHS	RLNANCP---	--NKNCDTDA	HKLSSAFILL	LKYF------	-EVADDLKDD	KCAEYAILWL
PCHAS_000140	-------MSN	EVCMYINKV-	DKLIKSN-AA	GTEGKIENHS	RLNANCP---	--NKNCDTDA	HKLSSAFILL	LKYF------	-EVADDLKDD	KCAEYAILWL
PCHAS_000070	-------MSN	EVCMYINMV-	DKLIKSN-AA	GTEGKIENHN	RLNANCP---	--NKNCDTDA	HKLSSAFILL	LKYF------	-EVADDLKDD	KCAEYAILWL
PCHAS_030080	-------MFK	EVCEHIHKI-	DTLIKLE-VT	DV-SYVEYDD	MLKSYCPKKN	YNTIECFGYD	EIVSAAFIFF	LENF----NS	SNDEEKFEGD	KLAEYAILWL
PCHAS_073200	-------MSK	GVCEAINSV-	NELINVK-ME	TEGTYIYYND	ILKANCPKAV	LKTNECHGYE	EIVGSTFIAL	LTLF---KSN	DDEVDALEDD	KLIEYAILWL
PCHAS_120020	-------MSK	KVCEAIKSA-	NELFNVE-KD	GSNTNINYDK	SLKAYCPPNH	LNNQECVHYE	QVVASAFIAL	LTLF--KNDD	DDEEDVLEDD	KLAEYAILWL
PCHAS_000060	-------MSK	KVCEAIKSA-	NELFNVE-KD	GSNTNINYNE	SLKAYCPPKN	LTKQECTNYE	QIVSSAFIAL	LTLF-RNFDD	DGDEDVLEDD	KLAEYAILWL
PCHAS_130020	-------MSK	KVCEAIKSA-	NDLFKVE-KN	DLGSFIKYDD	SLKAYCPPNH	LNKQECTYYE	QVVASAFIAL	LTLF--KNDD	DDEEDVLEDD	KLAEYAILWL
PCHAS_000150	-------MSK	KVCEAIKSA-	NDLFKVE-KK	GLETNIIYDD	SLKAYCPPKN	LNNKECVHYE	AIVSSAFIAL	LTLFKNGDDD	DEEEDVLEDD	KLAEYAILWL
PCHAS_000500	-------MIS	EVCGVIKEV-	DKCLSKT-IL	STGDECLDEL	GYTAYCPAKN	GKLGECRTNG	GKLSAWFIWL	LEMF--KTLT	NVDDFKDING	QYVEYAILWL
PCHAS_001100	-------MLE	EVCGTIKKI-	DDCLSRD-IL	STGDKCSDAE	LYTVYCSKKE	GVIGKCETNG	GRISAGFIWL	LVMF----DV	LCGGECSEKD	QYDEYAILWL
PCHAS_042010	-------MHH	EVCKAFRKA-	DYAFKDK-KP	DLDVINSQNG	PYVQDCPDNK	SKSHRCKNDL	EGMYVLCLHL	FHDL-FNLPQ	ETLKRENDDN	QYVEYIMMWL
PCHAS_000040	-------MLE	EVCGAINQI-	NKVLSDDILT	LGQHFPDDEL	GYTAYCPAKE	GEKGKCVTNG	DRISAGFIWL	LEMF--KALD	DVENLKDIND	QYVEYAILWL

Supplementary data 1 (CIR alignment)

```
PCHAS_000390   -------MIE V-CGVINQI- NKVLSDDILT LGQYFPDDEL GYTAYCPAKE GEKGKCVTNG DRISAGFIWL LEMF--KALD GVENLKDIND QYVEYAILWL
PCHAS_030180   -------MSK EVCEAIKFA- DEIIVFD--K KIKNYTFKND IFKVYCPQRK GPNRKCDSGG KILGAVFVAL LNFF---GSV EDYEENLKND SLSEYAILWL
PCHAS_130070   -------MSK EVCEAIKFA- DEIIVFD--K KIKNYTFKDE IFKVYCPQKR GPNRKCDSGG KILGAGFIAL LKTL---DGV DDYEENLKND RLSEYAILWL
PCHAS_011500   -------MSK QVCEAIKEI- DENVIFN--S VSEKYEFKDK MYGAYCANRK GGKGECDSNG KILGSAFISL LEIL---DGV DDYEEKLKND RLSEYAILWL
PCHAS_146850   -------MSK EVCKVINDI- EKNILFD--S GSQKYEFKND IFKAYCPRKR GGKRQCDSNG KILGAAFIAL LKNL-DSVED YEENLK--KD ILSEYAILWL
PCHAS_070100   -------MSK EVCEAIKGI- DENVVFD--P VSQKYAFTDE IYGVYCSTRK GEKGQCDSNG KLLGSAFIAL LNIF---DSF GDEEEKLENN KLGEYAILWL
PCHAS_060050   -------MSK ELCDAIDFT- DKNVVFD--Q KSKNYTFKDD IFKVFCSGGK GGKGQC-NSA QKLSSGFMGL LEYF-----K SIEDENLDGD KLAQYAILWF
PCHAS_030110   -------MSK GVCEQINFV- DENIVFD--Q KNKKYTYKDK IFETYCSSRN GEKGQCDNDG ELLSSAFIAL LKNF-----E NVDENKLESD KLAQYAILWF
PCHAS_130050   -------MSK ELCKAIDEI- DKNVVFN--T VSQKYTLEDK IYGAYCPSEN GEKGQCDSDE ELLGSAFIAL LKFF-----K NIDNGHLEDD KLAQYAILWF
PCHAS_030090   -------MSE ELCGAIKFV- DENVVFD--K NTQNYTFKDE IFKAFCSSGK DGKGKCDTNR EFLNSGIMGL LEYV-----K SIDEEELDGD KLAQYAILWF
PCHAS_146870   -------MSK ELCGGIDGI- EDFIVFD--S GSQNYKFKDD ILNAYCT--- --NNNCDSDG KKLGSAFIAL LKYF-----N SIDNVNLEDD KLAQYATLWF
PCHAS_130030   -------MSE ELCGGIDDI- EDFIVFD--S ESESYRFNDD ILKVYCSKEN GVKGECDSDS LKLSSAFIAL LEYF-----K SIEDKNLDDD KRAQYAILWF
PCHAS_040110   -------MSK ELCQGINFA- DKNVVFD--Q KSESYKFNDD IFKVYCP--- --NNNCDSNG LKIGSAFTAL LEYF-----K NIDNENSEDD KLTQYAILWF
PCHAS_042030   -------MSE ELCEGIKFA- DENVVFD--S ESQNYTFKDE IFKVFCSNGK GEKGQCDKES LKVSSGFMGL LEYF-----K SIDEEGLDGD KLAQYAILWF
PCHAS_060140   -------MSE ELCDGIKFA- DENVVFD--S ESQKYTFKDK IFDAYCPSEN GGKGQCDSDG LKVGSAFTAL LEYF-----K KIDGENPEGD KLAQYAILWF
PCHAS_030210   -------MSE ELCKAIDEI- EDFVVFD--S ESKKYTFKDK ILEAYCSSGK GENGQCDTES LKLSSGFMGL LEYF-----K SIEDENLDDD KRAQYAILWF
PCHAS_060060   -------MTE ELCEAIKFA- DENVVFN--S ESQDYTFKDD IFKAFCS--L SGKGECASDE LKVGSGFMGL LEYV-----K SIDEEELDGD KLAQYAILWF
PCHAS_050070   -------MSK GVCEQIDQI- EKYITFD--- SGSQNYKFND ILKAYCP--- --NKNCDTDE KILGSAFTAL VENL-----K SIDDEKPEDI KLGQYAILWL
PCHAS_040050   -------MSK GVCDAINEI- DKNVVLD--S ASQNYKFDEE IYGAYCP--- --NKICDTNE KLLGSAFMSL IELF---KNV DDYDDNFKND KLSQYAILWF
PCHAS_001050   -------MSK GVCDAINEI- DKNVVLD--S ASQNYKFVDQ IYGGHCH--- --NKNCDTDE KILGSAFMSL LELF---KDV NDYEDNLKNY KLAQYAILWF
PCHAS_083760   -------MSK ELCEKINFA- DENVVFD--P ESQNYKFNDE ILKAYCP--- --NKNCDSES LKLSSSFVGL LENF-----R SIDNEKSEDD KLSQYAILWF
PCHAS_140070   -------MSK ELCGAIKFA- DENVVFD--S KSERYSFKDD ILKTYCP--- --NQICDGDG KLLGSAFTAL LYYF-----K SIDEEKLEDD KLAQYAILWF
PCHAS_000180   -------MSE ELCDAINAI- DQNVVVD--S KSQKYKFENQ IYGAFCP--- --NKNCDTDE KTLGSAFMSL LKLF-----K SIDENKLESD KLSQYAILWF
PCHAS_001060   -------MSE ELCKAIDEI- DKNVVFN--T VSQTYTLEDK IYGAYCPSGN GEKGQCDSDD LKLGSAFMAL LNNF-----K SIDEKKLESD KLSQYAILWF
PCHAS_011450   -------MSE ELCEKINQI- EEYITFD--S TSQKYMVKNE ILNSYCPSEN GGKGQCGSNE ELLGSAFMAL LVNF-----K GIEDEKLEDD KIYQYAVLWL
PCHAS_060030   -------MSE ELCKQIDQI- EEYITFD--S ESQNYTLSDG ILKAYCT--- --NQKCDSDD LKLGSAFMAL LENF-----N SNDGEDPESD KLYQYAILWL
PCHAS_120030   -------MSE ELCGAIKFA- DENVVFD--S TSQNYNFNDG ILKAYCPKDK DGNGQCESDD LKLGSAFMAL LNNF-----K SIDGENPEGD KLYQYAILWL
PCHAS_030120   -------MSK ELCKAINEA- DEKIVLD--L ESKDYMFKDK ILEAFCPKKN GGKRECDSNG LKVASAFIAL LKYF---KSI DDYEDNLKND KLAEYAILWL
PCHAS_114640   -------MSS GVCEAINEV- DKYIVLD--P ESKDYTFKDN ILEAYCPKKK GGKRECDSNG LKVGSAFIAL LKLF--KSVD DEDKLE--SD KLAEYAVLWL
PCHAS_130350   -------MSY HVCEMFNSL- YSQLPDD-DN EEKRQNGEKL LYESFCPKIG ETYQKCSSEF EKISAGFVYL LGQL-FGNVN SEGEHEDQKD HYVYYGFLWI
PCHAS_030150   YTIKFYYNKV FQCQMFLEI- NRQLPDE-TN DLGEYIYNDE VYKAYCPNEL TGDHTCNSNI ERINAGCILL LNML-FNIDD DILESENQDT SFAQYIILWL
PCHAS_060120   -------MSA HVCEIFGNLY MQLPDGD--D EVNLGNGASL LYESYCPTNN GQHGTCNTDY DKIGAGFGYL LTQL-FNNVE SEEEHENQKG NYVHYGLMWI
PCHAS_041960   -------MSA HVCRIFQSLY NELPDDG--D EPKRENGAVV LYETYCPIND SGNQQCNSNL DKIIAGFGYL LPQL-FGNVD NEEDHEDQED NYVYYALLWV
PCHAS_130110   -------MSA HVCEMFDSL- YKQLPDN-GD EANQSDDTIL LYKTYCPING DEPGKCNSNL DKISAGLGYL LPQL-FGNVE SEEDHEEQEG NYVYYGLLWT
PCHAS_041950   -------MSY KACNIFRDV- DNQFNGK-SI NVEQFNTNSG LYAQYCP-VK QGSKRCVTDY EKLNAISGHA FMEL---TKN NKINLYSGHD PSIDFLVMGW
PCHAS_060130   -------MGM K-CETFLDV- DKLFINY-KA NEEQFNTSSG LYYQYCP-VK NGLRTCGTDY EKLSAIFGYA LMEL---AKN PKMDLYSEYD PSIAFLVMGW
PCHAS_130120   -------MGM KVCETFLDV- DNLFINY-EA NEEQLNGGYG SYKKYCP-IK RGVRKCETNY EKLRAISEYG FMEL---AKN DKVNLGSEYD PSADFVVMGW
PCHAS_070170   -------MEI E-YNLFLEV- DELFDDK-SV DV-VQFNANR KVAAYCP-EK NGYRSCNDDY QRINAIGSHL HMEL--DTKD PNIYGGGNNK RFMEYLIMWL
PCHAS_100060   -------MEI E-SNLFLGV- EELFDGK-SV DV-GKFNANY QVPTYCP-EK KGYRSCNNDY QRINAIGSHL HMEL--DTKD TNIYGGGNNK RFMEYLIMWL
PCHAS_146770   -------MAN KACGYLLDA- DEYFNNE-VV DE-NKFNKND SLKNRCPYEN KVSRPCQNNY ERINALAVYL YQNL-NSTST SLSGKGHGND RHIEFFMMWL
PCHAS_146790   -------MAS RACKFLLEA- DEYFNNG-IV DE-DKFKKNT ELHSYCPYDN GKRRPCKNNY ERINALGVHL YQKL-NKTTK NLNGTGHHEN RHIEFFMIWL
PCHAS_060160   -------MAN KACSYLIEA- DEYFNNG-VV DE-NKFNKNS SLQDRCPYEN RSPRPCKNNY ERINAVGAYL YQNL-SKTGK NISGTGHNGD RHIEFFMMWL
PCHAS_000260   -------MAI RACKFLLEA- DEYFNNG-VV DE-TKFNKNS SLENRCPYEN KRARPCKNNY ERINALAVYL YQNL-TKTTK NLNGTGHHEN RHIEFFMIWL
PCHAS_130280   -------MAN KACSYFIEA- DEYFNNG-IV DE-NKFNKND SLKYRCPYEN RTLRPCQNNY ERVNAVGAYL YNNL-LNVGS SFKGIGNNDN RHVEFFMMWL
PCHAS_083720   -------MAS RACKFLLEA- DEYFNNG-VV DE-IKFNKND SLENRCPYEN KKVRPCKNNY ERINALAVYL YQNL-TKTTK NLNGTGHHEN RHIEFFMIWL
PCHAS_090010   -------MSS KLCKLLLDV- DDLFTDG-TV DV-NKFNKLD SYKKYCPYEN GKLRDCKNNY ERINALGEYL YQKL-PKNSD EFKGEGINNN LYIEFFMMWL
PCHAS_114600   -------MSS KLCKLLLDV- DEYFNNG-IV DE-GKFDKNK SLQSKCP-QK GQENKCTTNY ERINALGEYL YQKM-PKNSN EFKGEGINNN LDIEFFMMWL
```

Supplementary data 1 (CIR alignment)

PCHAS_146860	-------MNY	SACDLFFEV-	DELIDDK-SV	DVDSFNDLSS	YFNEYCP-EK	NGSKICSTDY	ERMIAVGAYL	FMEF---INE	NNIDLNSDKE	YHSEYFIMWI
PCHAS_130090	-------MSY	KTCDIFREV-	DELFDGK-SI	DVDNFNAFSS	LYNEYCP-VK	NGSKSCDTEY	ERIAAVAGYI	FMNF---ILD	NSINLHSDDD	RHLEYFIIWI
PCHAS_030170	-------MDT	ESYKLFREV-	DKLFDEK-SV	NV-GKFNTYQ	DVADYCP-EK	NGYKSCDNDY	ERINAIGGHL	IKQL--YFKN	SSINGGNNNN	RFIEYFIMWL
PCHAS_130060	-------MDT	ESYKLFLEV-	DKLFDDQ-SV	KV-ANFNAYH	NVSDYCP-EK	DGYKSCDNDY	QRINAIGAYL	ITQL--YLKN	SSINGGNNDK	RFIEYFIMWL
PCHAS_030200	-------MSY	NACKLFSEL-	DELFNSN-SV	DEDSFNGLSV	LYNEYCPQDK	NGDKKCITDY	DRISAVAGYL	FMYF---ITE	NGIDINNNND	YYIQYFVMWI
PCHAS_060080	-------MSY	NACNLFHGL-	DELFEEG-FG	DVKELDSLSI	LFNDYCP-ER	GESKRCDTDY	ERISAVAGYL	FMNF---IAD	NNIDINNDNN	YYIQYFVMWI
PCHAS_060110	-------MDT	KLCNLFINV-	DKLFTKG-NV	NE-TLFNTSN	LYKKYCP---	--NGKCNTNY	DRIGALCEYL	LAEL-PKLNN	KPNGSKDNVN	QYYEYVFMWL
PCHAS_030140	-------MET	KLCKLFIDV-	DKLFTKG-NI	NV-NKFNNSD	SYKKFCP---	--KGGCNTNY	DRLGALCGYL	LAEL-PKNYD	KQKGGNNNGN	LDYEYIYMWL
PCHAS_130100	-------MFP	FDCKLFIDV-	DKLFTKG-NI	NE--QIFNK-	SYKQFCP---	--KGVCNNNY	DRIGALCEYL	LAEL-PKNDD	KLKGGNNNGN	RDYEYIYMWL
PCHAS_041990	-------MDT	KLCKLFVDV-	DKLFTKG-NV	NE-TLFNTSN	LYKKYCP---	--NGKCNTNY	DRIGALCEYL	LAEL-PKLNN	KPNGSKDNVN	QYYEYVFMWL
PCHAS_130080	-------MDK	NVCKLFVDT-	DEVFNHG-TV	NE-SIFNTSN	LYKKYCP---	--NGNCNTNY	DRISALCDYL	LTEL-QKYDK	KQNDSNNDVN	QNYEYVFMWL
PCHAS_030190	-------MDK	NVCKLFVDV-	DKLFTKG-SV	NE--TLFN-D	PYKNFCP---	--KGGCNTNY	DRLGALCGYL	LAEL-PKLNN	NPKGSEDNAH	QNYEFIFMWL
PCHAS_060090	-------MDK	NVCKLFVDV-	DKLFTKG-SV	DE--TLFN-D	PYKNFCP---	--KGGCNTNY	DRLGALCGYL	LAEL-PKLNN	NPKGSEDNAN	QNYEFIFMWL
PCHAS_041970	-------MNK	NVCKLFIDV-	DKSFKKG-TG	NV---NTFDN	TYTKYCP---	--EEGCNTNY	DKISALCEYL	LEEL-PKIDD	KQKGSEHNVY	RDYEYIFMWL
PCHAS_041980	-------MDK	NVCKLFIDV-	DKLFTKG-SV	NE---TLFND	SYKNFCP---	--KGGCNTNY	DRIGALCEYL	LEEL-SKNDD	KQKGDNNNVN	QNYEYVFMWL
PCHAS_042020	-------MYR	DVCKIFQEV-	DEVFKDN-KP	DL-NKIFEFD	KYRRYCPWDI	TGVNNCQHDV	AGIDAVYSYL	FKEI-HQLPL	DQQKYENNDN	QYTEYMLIWL
PCHAS_060070	-------MSK	EVCKIFKDA-	DNFFKGG-NP	DLDKINKEIG	ENIRDCLGNK	SDTYTCKTDF	DGINALYCHV	VTEL-HKIPI	ETQEHENNDY	QYIEYAIIWI
PCHAS_011330	-------MDN	EACKLFRMV-	NANFNKE-IV	DL-DKFKQFT	VCHRYCPGKK	RQLRDCNNNY	ERINAIGGYL	RSQL-SGVKK	EIQTRENNDN	QYIEYMLIWL
PCHAS_000220	-------MDN	EACKLFRMV-	NANFKNG-VV	DA-DKFKQFT	ECHRYCPGRK	KQRRDCNNNY	ERINAIGGYL	RVKL-SSIKN	DIQTRENNDN	QYIEYMLIWL
PCHAS_042070	-------MAI	NACKLLLEV-	DGYFKNE-NV	DE-EKFNKNN	SLVYRCPYED	RKLRPCENNY	ERINALGVYL	YEKL-GKIAN	DLKGKGNNDN	RHIEIFIMWL
PCHAS_110030	-------MAD	KACTLLREV-	DAYFNNE-NV	NE-EKFNNSG	LFTYRCP-RK	GKEYICTTNN	ERINTLGVYL	YENL-NKISK	DFKGEGNEAN	RHIEIFMMWL
PCHAS_140140	-------MAI	EACKILRDV-	DGYFKDE-IV	DE-SKFNNSG	LFTYKCPKQN	RRFRPCENNN	ERVNTLGVYL	YEKL-NGIAN	KLNGEENNAN	RHVEIFMMWL
PCHAS_010040	-------MAG	KACKLLRDV-	DAYFNNG-IV	DE-NKFNSSG	SFIYKCPYEN	RKPRGCKNNN	ERINALGGDL	YDKL-ARIAN	DFKGEGDNGN	RHIEIFMMWL
PCHAS_073150	-------MAS	EACKILRDV-	DGYFKDE-IV	DE-SKFNNSG	LFTYKCP-RK	GKESKCTTNN	ERINTLGAYL	YTKL-FKISN	ILKGDGNNAN	RHIEIFMMWL
PCHAS_073130	-------MGY	QMCETFIVA-	DQVINGENAN	ITMDDISKNP	GFKQYCP---	--NQKCETRR	KRISALSTYL	FMQL------	---RTMKSAG	QYDEYFLMWL
PCHAS_040060	-------MTT	YMCETFLEAD	KIINRENDAR	MKMEDIDKKS	PYYEFCP---	--NRKCVTDV	QRIGVMTTHV	FLKV------	----KADKNN	EYGEYFLMWL
PCHAS_000430	-------MDP	NMCETFLEAD	KIINGENGAR	MKMEEIDKKS	SYYEFCP---	--NKKCLTDV	QRIGVMTTHV	F---------	-LKGKADKNN	EYGEYFLMWL
PCHAS_104200	-------MDP	NMCETFLEAD	KIINGENGAR	MKMEDIRKSQ	SFNGFCP---	--NNKCVTDE	QCIGAMTMYV	FSKV------	----GADKNN	EYGEYFLMWL
PCHAS_114720	-------MDP	NMCETFLEAD	KIINGENGAR	MKMEDIRKSP	SFNGFCP---	--NNKCVTDE	QCIGAMTMYV	FSKV------	----GADKNN	EYGEYFLMWL
PCHAS_000770	-------MDK	HMCEFLIEA-	DGYFNGK-NV	NT-EKINEDP	TIKGYCR---	--NGGCKTNE	DSINALTAYI	IKEF------	KKSIKSDEYN	KYDECFFMWI
PCHAS_130220	-------MDK	HMCEFLIEA-	DSYFNGK-NV	KM-NEINKHA	TIKGYCY---	--NDDCKTNE	DSINALTAYI	IKEF------	KTSINQHDYN	KYDECFLMWL
PCHAS_070040	-------MDT	EMCKLFREA-	DSYFNGK-DV	DT-TKFNEHK	TIKSYCR---	-DDGGCKTNE	ERINALIAYI	IMDF------	KRSIEKNEYN	DYDEYLLMWI
PCHAS_011490	-------MDP	QMCEFLIKA-	DKYFAGN-KV	DI-KEINKNK	SIKSYCH---	--NSDCKTNE	DSINALAAYI	IMIF------	KRSIKTNEYS	HYDECLLMWL
PCHAS_070060	-------MDS	KMCEFLIKA-	DKYFAGN-KV	DI-KEINKNK	SIKSYCH---	--NSDCKTNE	DSINALAAYI	IMIF------	KRSIKTNEYS	HYDECLLMWL
PCHAS_030070	-------MDH	KMCKYLNIA-	DSYFKGE-NV	NT-KIINKHS	TIKYYCY---	--NGVCKTNE	AGINALAAYI	FKQF------	KVSIEANEYN	KYDEYLLMWL
PCHAS_137110	-------MDH	KMCKYLNIA-	DSYFKGK-NA	DM-KIINKDP	AIKYYCY---	--NGVCETNE	AGINALVAYI	FNQF----KR	SIEANE--YN	KYDEYFLMWL
PCHAS_114700	-------MTT	RLCEFLIEA-	DGYFNGK-DI	DM-KEINKNT	KIIGYCS---	--NGGCKKNE	EHINALTLYI	YMGF-----K	NSMKRQSEYN	KYDECLLMWL
PCHAS_137030	-------MTT	KLCEFLIEA-	DGYFNGK-DV	DM-KEINKNR	KIIGYCS---	--NGGCKKNE	EHINALALYI	FKEF-----K	NSIKRQTKYN	DYDECFLMWL
PCHAS_011480	-------MRK	HMCKLLLEG-	DSYFNGK-DL	DT-NEIIKKT	SIKGYCR---	--NDRCKKNE	ARINALTLYI	HMEF-----K	NLIKKKSEYS	NYDEWLLMWI
PCHAS_050040	-------MNH	ELCGLLIEA-	DGYFNGK-DV	DA-QRINDEP	TIKGYCS---	--NDGCKTNE	ARINALAAYI	LMLF------	KKSIRKDEYN	DYDECFLMWL
PCHAS_000470	-------MNM	RRCKLLLEG-	DSYFNGK-DV	NT-NEFNKHS	TIKAYCR---	--NDDCKTNE	ERINALTAYI	FKEY-----K	GKTSRRTKYN	DYDECFLMWL
PCHAS_000170	-------MNH	ELCGLLIEA-	DGYFNGK-DV	DA-QRINDEP	TIKGYCS---	--NDGCKTNE	AYINALAAYI	FMLF------	KRSIQKDEYS	EYDECFLMWL
PCHAS_130170	-------MSK	HLCMLLLEG-	DSYFNDE-NV	DT-EKINKDI	TIKGYCR---	--NGSCKTNE	ESIDALAAYI	FKKF-----K	DSIKVKQRYN	NYDECLLMWI
PCHAS_000660	-------MDS	KMCNLFREA-	DGYFNDE-NV	DT-KKINKNT	KIKGYCS---	--NNGCKTNE	DYINALSAYI	YKEF-----K	NLIKRNQKHN	DYDEYLLMWI
PCHAS_040080	-------MNT	KMCKLLLEG-	DGYFNDE-NV	DT-KKINKNT	IIKGYCS---	--NNGCKTNE	DYINALSAYI	YKEF-----K	NLIKRNQKHN	DYDEYLLMWI
PCHAS_070070	-------MQN	HMCNLLLEG-	DSYFNDE-NV	DT-QKFNKHS	TIKAYCR---	--NGGCKTNE	ERINALNAYI	FKTF-----K	DSIVIKKKYN	HYDECLLMWV
PCHAS_137090	-------MSK	HMCKLLLEG-	DSYFNGK-NV	NT-EKINEDP	TIKGYCN---	--NGVCKTNE	ESISALTAYI	FMKF-----K	DSIVIKRKYN	DYDECILMWL

Supplementary data 1 (CIR alignment)

```
               ....|....| ....|....| ....|....| ....|....| ....|....| ....|....| ....|....| ....|....| ....|....| ....|....|
                   105        115        125        135        145        155        165        175        185        195
PCHAS_000950   SNIIRHFNYK HTPHVQDFYT TFIKEG---- --------NW YNDFRDKINK KNDIMKLQIN QIYNLYELLN ILCKAITKYD ENPSNSSECS NFANKWEERS
PCHAS_001010   SNIIRHFNYK HTPHVRDFYA TFIKDS---- --------DL YKDFHGKINE KNEIMKIQIG QMYNLYELLN ILCNAITKYE ENPSNCPECS KFTNKWEEKT
PCHAS_140090   NHKLNQYPEY GVSNLDDFHN TYIKDI---- KETTD--IEG YNAYMDFIEK KQDMMNMDIK DMSKFYASLK SLCDMQTKID ANKSNCASYL EDSQKFVDEY
PCHAS_000730   CHKLNKNSQN GINNLNDVYN KYIKGNKEFF EKIND--AGA YNSYEEFINK KQDMVNVNIK DMSKFYEALQ ILCKMYTKID ESKSNCTKCL DDAQKFADEY
PCHAS_070160   CYELNKK--N EISNLNEFYN KYIKAIEEYV KGSHA--AEA YNSCMNIINK KQYLMSVDIK EMSKIYEALK FLCKLYTGCN DKEKNYSNYS QDANDFVKEF
PCHAS_030020   CYKINLMSYD GIRNLNDFHN KYIKDKESDI QKMVD--VEA YNIYMDFINK NQDLMNMDIN GISNFYAPLK SLCNMYNKFD AKNNSCAKCS EDAKEFVEKY
PCHAS_000360   CYKLNLMSYE GISNINDFHN KYIKDKESDI QKIVD--VEA YNIYKDLINK KGDLVNMDIK IMSKFYETLK FLCKLYTGCN EKETNYPNCS QDANAFVKEF
PCHAS_110020   CYKLNKKKEN EISNLNDFHN KYIKGNENHI DKING--AGS YNSYKNIINN KKYLNTIDIK EMSKFYVPLK SLCKLYTEYS KKKKNYTTCS QDAQDFAKHF
PCHAS_050060   FYKLNQKKEY EISNLNDFQK KYIKDNENFI DKING--DGS YNSYLDIINK NPYLMSIDIK EMTKLYVPLK SICKLYTECD GKKKSYTNCL QDAQDFAKHF
PCHAS_010030   CHKLNKNSQN VVSNLKDIYD VYIKDYEKDI EKISG--ADA YNSCKDIINK KIYSMRIDIK EMSKLYEALK TLCKLYTECN GKKQNCTKCL QHAEEFVKSF
PCHAS_030030   CHKLNKNSQN GVSNLKDIYD VYIKDNKKDI EKMSG---EA YNSCKDIINK KIYSMPIDIK EMSRLYEALK ALCKLYTECD EKKEKYTSCS RDAQDFANEF
PCHAS_000350   CHKLNKNSQN KVSNIKYIYD AYIKENEKDI EKISG--AEA YNSCKDIINK KIYSMSFDIK EMSRLYEALK ALCKLYTECN EKKKSYTNCS KDAQDFAKHF
PCHAS_011530   CYKLNQRSEN GTSDLNDFHN KYINGIETHI LKLPN--VNA YNSYKDII-K KQDMKVMDIK DISKLYEPLE NLCKLYTGCD EKKGNYTNCS KDAHDFASNF
PCHAS_073160   CYEINKNKNN EISNLNDFHN KYIKNIEKYI MKIPN--LGA YNSYKGIIDK QQNSMPIGVK EMFKLYEALK ILCDMYTECD KKKQNYINCL QDANDFAKHF
PCHAS_070090   CYQLNQKTKI SIDNLNEFYN NYIKGIEKYS NKLDA---DN YNSCMDIINK KQYLMNMDIK EMSKLYEALK KLCKLYNECN EKNNNYLNCS QDAQDFANNF
PCHAS_000580   CYQLNKKTVN TIDNLNDYYN TYIKGIEKYS DKLED--AEN YNSCKDIINK KQYLMNMDIN KMSKLYEALK ILCDMYTECN GKEESYTDCS KDAQDFVQKF
PCHAS_140130   IYMLRLKDDD KTGNVTKFNN KCINYDMNYI NSIKD--TNA YKIYKDLINS KLYLMNNDIK NISMFYDAFK SLCNVYNEFN EDDPVCSTYL VKAKEFVKKY
PCHAS_140040   IYTLKLKSDD KTDNVMKFYD TCINGGDNYI NSIKNTNINA YNIYKDPINS KLLSMNKGIN DISKFYDAFK SLCNMYNGFN PDSPNCTQCL DDAKKFAEKY
PCHAS_000740   IYMLRLKGDG QNGNVMKFHN TCINPAQGYT NSIKDTNTNA YNIYKNLIDS KLLLMNEGIK DISKIYDAFK SLCNMYNGFD DDDSDCTKNL NDAKKFVEKY
PCHAS_100030   IHMLRLKNDG QIDNVINFYN TCIQPTQEYI NSIKDTNSNA YNIYKKLIDN KLYLMNKDIK DISIFYDALK SLCNMYNKFD DDDSDCTQCL DDAKNFAKKY
PCHAS_001120   IHMLRLKGDG QIDNVIKFYN TCIQPTREYI NSIKDTNANA YNIYKKLIDN KLYLMNRDIK DISKFYDAFK SLCNMYNGFN PDDSDCTQCL DDTKKFAKKY
PCHAS_011520   IYTLKLKSDG NV---MEFYN TCINGDDNYI KSIKD--TNG YNIYKDPIKS KLLSMNTGIE DISKFYDALK SLCKMYNEFD RDKPDCTQCL DDANKFAEEY
PCHAS_070030   IYMLRLKGDG QNGNVMKFYN KCINDDGNYI KSIKDTNTNA YKSYMDFISR KLFLMNKDIK DISIFYDAFK SLCNMYNEFD GDKPDCTQCL DDAKKFAKKY
PCHAS_073190   IYMLRLKGDD NV---TEFYN KCINPAQGYT NSIKDTNTNA YKSYMNLINT KLFLMNGGIN DISKFYDAFK SLCNMYNEFD GDSPYCTQCL DDAKKFAKKY
PCHAS_000570   IYTLKLKSDD KIDKVMEFYN TCINPTSEYI NYIKD--TNA YNTYKDLINS KLYFMNTDIK DISNFYDAFK SLCNMYNEFA EDDSRCTQNL EDAKKFVEKY
PCHAS_114730   IYTLKLNNGD KV---MEFYS KCINPAEGYT NSIKDTNTNA YNIYKDPINS KLLSMNKGIN DISIFYDALK SLCKMYNDFA DDDSDCTKNL GDAKKFVEKY
PCHAS_010020   SYMLNQINDK TIKNLNDFYD QYVNKRDDYK TSIND--VSE YNNYKQIIDK RKDFFNMDMK IISKFYNTLK LLCNMYNEIN DEVTNCTKYY GTAIKITDEY
PCHAS_000280   SYILSLKENN NISNLKDFYD LYINSNDKYN DKITG--VKD YTSYKDLIDK NNYFLSMNMK IVSKFYTSFK SLCSMYNEIE GNKSNSTKCL EEAKNFVDEY
PCHAS_000490   SYILSLKENN NISNLKDFYD LYINSNDKYN DKITG--VKD YTSYKDLIDK NNYFLSMNMK IVSKFYTSFK SLCSMYNEIE GNKSNSTKCL EEAKNFVDEY
PCHAS_000030   SYILSRKPNE EISNLKKFYE KYIESGEGYE TSIDD-VSDY YSSYKNLIDK KNDFMNMDVN YISTFYEAFK ILCSMYNDIN SDPPDCTKHF TKTKEFVSKY
PCHAS_000090   SYILSRKPNE EISNLKKFYE KYIESGEGYE TSIDD-VSDY YSSYKNLIDK KNDFMNMDVN YISTFYEAFK ILCSMYNDIN SDPPDCTKHF TKTKEFVSKY
PCHAS_000270   SYILSRKPNE EISNLKKFYE KYIESGEGYE TSIDD-VSDY YSSYKNLIDK KNDFMNMDVN YISTFYEAFK ILCSMYNDIN SDPPDCTKNF TKTKEFVSKY
PCHAS_000120   SYILTRKPNE EISSLKKFYE KYIENGEEYE TPIDD-VSDY YSSYKNLIDK KNDFMNMDVN YISTFYELFK ILCSMYNDIN NDMPDCSKYF TKAKEFSDKY
PCHAS_001090   SYKLKK-NKK ITDEISDIYG ELKKNN---- ----NK-ISE YYNYKEQIN- --NLMNMDFN FISKFYEALQ ILCDISTQFD DDTTNCENCL GDAKKFVAEY
PCHAS_011510   SYKLNQKSEY SDMKLSDFYT KYIVNN-NSY NENING--DD GLTYKEIINR NNDLMN--IK EISKFNYPFG MLLSLYYLVH LKNLDCKNCS KNANEFVQNF
PCHAS_070050   SYKLNRHSKC SGTNLNDFYT NHIEKN-EYY NKKIKD--DD TTTYKDIIDK KKDLMD--IK EISKFSSLFS ILFYLYNVFH DKSLNCQQNL NLAKNFADIF
PCHAS_000720   SYKLSRIRQH NFAKLNDFST KYIVNN-ECY NEKIKD--GD TTTYKDIIDK KKDLMDMNII EISKFNGLFS ILFYLYYLFH GECLDCEKNS SLANNFADIF
PCHAS_070020   SYKLNTVKDK CATNLNEIYT SYIETN-NYY NKNIKD--GD TTTYKDIIDK KKDLMNMDIK EISKFNGLFS ILFYLYYLFH GERLDCQQIW ELAKNFADIL
PCHAS_104260   SYKLNRHSKC SATNLNEFYT NHIKTN-KCY NEKIKD--GD TTTYKDIIDK KKDLMD--IK EISKFSSLFS ILSYLYYLFH GECLDCEKIW KLAKNFADIL
PCHAS_140020   SYKLNRHSKC SATNLNEFYT NHIKTN-KCY NEKIKD--GD TTTYKDIIDK KKDLMD--IK EISKFSSLFS ILSYLYYLFH GECLDCEKIW KLAKNFADIL
PCHAS_030060   IYKLKIKKNN TVTNLNDFYN NYIEKN-KCY KDKIN---GD GSTYKDIINK KKDLMDMNIN EIFKLEAPFN ILYYLYHEIS DQKSFCKEYS KYAANFADQF
PCHAS_000750   SYILNLYLKD KLTNLSEFYT NYIEKN-KCY NEKING---D GSTYKEIIDK KKDLMDMNIS EIFKLEAPFN ILYYLYHQIY DQTSFCSEYS KYATKFSDQF
PCHAS_000680   SYKLKIKENN IVNKLSDFYN SYIETN-NDY NKNING--ND GPTYKEIIDK KKDLMDMNIS EIFKLEAPFN ILYFLYYEIS DVAVDCKKNL SIA-NNFAKE
```

Supplementary data 1 (CIR alignment)

```
PCHAS_000420    SYKLKIKENT IIKKLSDFYD SYIETN-EYY KDKING--DD GLTYKEIIDK KKDLMDMNIS EIFKLDAPFN ILYFLYYAYN YDYWDCAKNS NYANSFVNQF
PCHAS_000100    SYKLKIKENP IIKKLSVFYD SYIERN-EYY NKNING---D NLTYKEIIDK KKDLMDMDIN EIFKLEAPFN ILYYLYHQIY DKNSFCSEYS DYANQFVQKF
PCHAS_000310    SYKLKIKENP IIKKLSVFYD SYIERN-EYY NKNING---D NLTYKEIIDK KKDLMDMDIN EIFKLEAPFN ILYYLYHQIY DKNSFCSEYS DYANSFVNQF
PCHAS_040040    SYKLKIKENN MIKKLSDFYN SYIERN-EYY NKNING--GD GLTYKAIIDK KKDLMDMNIS EIFKLEAPFN ILYYLYYKIS DKHTDCEKNL NDAKKFVDKI
PCHAS_120060    SYKLKIKENN RIKKLSDFYN SYIETN-DCY NKNING---G NLTYKAIIDK KKDLMDMDIN EIFKLEAPFN ILYYLYDQIS DEKSFCTKNL DYAKTFAEKY
PCHAS_104230    SYKLKIKENE KIKKLSVFYD SYIKTN-DCY NKNING--GD GLTYKAIIDK KKDLMDIDIN EIFKLEAPFN ILYYLYNVIH DEHPDCEKNL DSAKNFAEKY
PCHAS_030040    SYKLKIKDNP IIKKLSDFYK SYIDTN-NYY NENING--GD GLTYKAIIDK KKDLMDMNIN DIFKLEAPFN ILYYLYDQIS DEHPDCEKNL DYANDFAEKY
PCHAS_000340    SYKLKIKDNP IIKKLSDFYK SYIDTN-NYY NENING--GD GLTYKAIIDK KKDLMDMNIN DIFKLEAPFN ILYYLYNVIH DEHPDCEKNL DYAKNFAEKY
PCHAS_001110    NYKLNQNSED GINTLNDFYT KYIEKN-THY NQKISK-AND NKTYKNIIDK KKNLMNMDIN DIFRFYDAFE SLCSMYNELG DENPNCGKCS QKADEFVNKY
PCHAS_120040    GYKINQILNG EFTTLKDFYT KHIKNN---- --------KD DVDYKDHLDN KINSMDINIK DTSNIYEAFE ILCNMYTVYN ENDKKCTNCS QNAEKFVQKI
PCHAS_000130    GYRLNQILDE EITTLKDFYT KYIKDN---- --------KD DVDYKDRLDD KINSMDINIK DISNIYEAFG ILCEMYTAHN ENDKECTNCS QYADEFVQKI
PCHAS_000400    NYKLNQISNE ETTTLKDFYT KYIKDN---- --------KE HVDYKDHLDN KIYSMNIDSE KIYNIYEAFG ILCKIYTAHN ENDKKCTNCS QNAEEFVQKI
PCHAS_040020    NYKLNQISNE ETTTLKDFYT KYIKDN---- --------KE HVDYKDHLDN KIYSMNIDSE KIYNIYEAFG ILCKIYTAHN ENDKKCTNCS QNAEEFVQKI
PCHAS_000110    SYMLNQISNE GIPTLKDFYT NNIEKN-TKY TRHVSS--AN DSNYKGIVDK KINLMNINKN IISKFYDVLK SLCSMHTECI GCKSNCTDCL EKAKDFADKY
PCHAS_120050    SYILNQMPNE GIHTLKNFYT NHIETN-TNY TSHVSS--AS DSNYKGIVDK KIDLMNMNKS IIPKFYDIFK SLCNMYNELD KNESNYTNCL KDAQNFVDEY
PCHAS_104250    SYMLNQISNE GTPTLKDFYA NNIEKN-KKY TNNVTS--AS DSNYKEIIDK KINLMNANKN IIPKFYDIFK SLCKMYNELD QNEPNYTNCL ADAQNFVNEY
PCHAS_000320    SYMLNQISNE GISTLKNFYT NHIETN-TNY TNNVTR--AS DSNYKEIIDK KINLMNANKN IISKFYDIFK SLCNMYNELD ENKPNYTKCL ADAQKFVDEY
PCHAS_000410    SYMLNQISNE GISTLKNFYT NHIETN-TNY TNNVTS--AS DSNYKEIIDK KINLMNANKN IIPKFYDVFK SLCSMYSELD DDEPDYTNCL KNAQNFVDEY
PCHAS_140030    SYILTRKTND GINNLNDFYN MYIKDK-EKY TAKING-VTG YTSYQNLIDK YKELMTIDIQ DMSKLYPPLK SLCDLYNGCI ICQSDCQNCL EKANDFAKKY
PCHAS_114740    SHILSLKENE GIKNLNGFYE KYIKDK-EKY NNHIPG-VHG YTSYQNIIDK YKELMTDDIE YMLQFYGPLK SLCEMYNACN LNRSKCTNCL EKAKDFADKY
PCHAS_040030    SYKINENTQT IIKSLNDFYT KHIEKN-ANY SEKITK-SGD NKTYKDIINK KQNLMNMNIN DISQFYDALK LLCSMYNELS DDNPNCEKCS KDAKEFVNKY
PCHAS_070130    SYKLNQNPQK GINTLNDFYT RNIGKN-THY TSTTDD-IDV YKSYKDFIDK NNDLMTVNIK EISQFYAPFK SLCGMYIECN GKKTNHTKCL EKANEFVENF
PCHAS_073180    SYKLKQNPQN RINRLNDFYT KHIEKN-AQY NTKITT-VSD NKTYRNIIDK KKDLMDMDIN DIFKFYAPFK SLCDMYNELD KENQECENYF RKANELVENF
PCHAS_000300    SYKLNKYPQN KITTLNDFYT EHIEKN-KYY NVKIT---KS SKTYKDIIDR KHDLMNIGIK EISQFYDAFK SLCDMYNELD KENQECANCS KKANEFVANF
PCHAS_120070    SYKLNKYPQK GINTLNDFYT EHIEKN-EYY NVKIT---KS SKTYKDIIDR KHDLMNIGIK EISQFYDAFK SLCDMYTELD KKNQDCENYF RKANKLVENF
PCHAS_001130    SYKLNKYPQN KITTLNDFYT KHIEKN-EYY NVEIT---KS SKTYKDIIYR KHDLMNIGIK EISQFYDAFK SLCDMYNELD EENQDCENYF RKANKLVANF
PCHAS_100040    SYKLNKYSQK GITKLNDFYT QHIEKN-KYY NVEITK-SSD KKTYKDIIDR KHDLMNIGIK EISQFYDAFK SLCDMYTELD KENQDCEYYF RKANELVENF
PCHAS_000560    SYELNKYSQK GITKLNDFYT QHIEKN-EYY NVKIT---KS SKTYKDIIDR KHDLMNIGIK EISQFYDAFK SLCDMYNELD KENQDCENYF RKANEFVKNF
PCHAS_050020    SYKMNQHPSQ KF-GTNDIYN NFKQYG---- --------YW NRKHNNYIEQ IKKY--VDIK DMTKLHEAFI LLCNMYTEID ENKSNCTKCS QKASEFVKKF
PCHAS_000020    SYKINQHPNK KF-GTNDIYN NFKKYN---- --------YW NLNHNNYIEQ IKKY--VDIK DMTKLHEAFI LLCKMYTEFN DETKNCTKCS QKASEFVKSF
PCHAS_000290    SYKINQHPNQ TF-GTNDIYN NFKQYG---- --------YW NRKHNNYIEQ IKKY--VDIK DMTKLHEAFI LLCNMYTEID ENKSNCTKCS QKASEFVKKF
PCHAS_060020    GYKLNKYSNI KLTILNDFYT KIKINS--KY IMHITG-NET YKNYKDHIDK KPDLMNMNIK TIFKFYEALQ ILCNMHNEDK EKNTDCTKCS QDAEKFVESF
PCHAS_001040    CYKIQQYQNG EIIALKDFIT EHIETN-TNY NWKTKK-GIG NKSYKEFIDK KQSLINMDVK IMSKFHEALQ ILCNMYNENK EKNINCTKCS QNSEEFLKIF
PCHAS_114750    SYKIQQYWNI ETTTLKNFIT EHIETN-TNY NEKIKQ-GKG NKNYKEFIDI KQSLINMDVK IISKFHEALQ ILCNMYNEDN AKNIDCAKCS SKASEFVEKY
PCHAS_000140    SYKIQQYWNI ETTTLKNFIT EHIETN-TNY NEKIKQ-GKG NKNYKEFIDI KQSLINMDVK IISKFHEALQ ILCNMYNEDN AKNIDCAKCS SKASEFVEKY
PCHAS_000070    SYKIQQYWNI ETTTLKNFIT EHIETN-TNY NEKIKQ-GKG NKNYKEFIDI KQSLINMDVK IISKFHEALQ ILCNMYNEDN AKNIDCAKCS SKASEFVEKY
PCHAS_030080    SYKLNQNPQK GITTVYDLYT KHIEKN-TNY NQKIID-DSD DKISKKHINK KLELMSMEIN VISKFYEALQ ILCNMYDEDK EKNPDCTKCS QYAEEFVKSF
PCHAS_073200    SYKLNQKQNI KFKNLKDFYD KYIKDN-GNI YNNDITGVDA YNSYKDIINE KINMKTINIK EMSIFYDLLK LLCDIYTEFD SNNSDCTKCS QKANEFVENY
PCHAS_120020    CYKINQ-ETH KFSNLNKFYN KYIKGI-EKH FSEENGA-KA YNSYKDLINN KICSNTIDIS NMSKFYEACE LLCKMYYECN GSSKDCTACL EKAKEFSEHF
PCHAS_000060    CYKINQ-EKH KLNNLSAFYK KYIMDI-EKD FMEENGA-NA YKSYKDIINN IICSKTIDIS NMSKFYEGFE LLCKMYYECN GSSKDCTACL EKAKEFSEHF
PCHAS_130020    CYKINQ-ETH KFSNLNKFYN KYIMNN-EKN IKESNG-AEA YKSYKDIINN KICSNTINIK ETSKLYEAFE ILCKMYNELD DDAKNCTKCL EDANKFVSKY
PCHAS_000150    CYKIDQ-GTY KYSNLSAFYK KYIMDI-EKD FMEENGA-NA YKIYKDIINK QICLKTINIK AMSKLYEALE ILCKMYSELG DDAKNCTKCL DDANKFVSKY
PCHAS_000500    NSKNNLINSD TYISTTTIYD ILEINS---- -------SYW YNEFRDKIEK KRNAMNFGDY YMHKLYNLLK EICSTINKYN EDKSYPTEYL KHANKCVNTY
PCHAS_001100    SSKYNLISPE YDVNITGIYD ILERNN---- -------PIW YNKYRDRIGK KRNVMDFGDF HMGNLYNLLK EMCILITKYN EDRSYQDVYL KYANNCANIY
PCHAS_042010    GDRLFNIESY SSSTLIDFYN NYLIKL---- --------PK FNSYKSLIEK KKHLKDSHIV YMRRFYLLFQ EVCYISLKYS KNNLNMDKMK RDYTFLHNKY
PCHAS_000040    SSKNILINPD MLVSVTGIYD ILERNN---- -------PIG YKEFRNKIEK KKQLMNFGDY HMGKLYELLK EMCTLITKYD QDRSFPDSYL NHANKCANLY
PCHAS_000390    CFKNKLINPD MYVSITGIYD ILERNN---- -------PIW YNEFRNKIEK KKRLMNFDDY HMGKLYELLK EMCTLITKYN VDSSHPDAYL NYA-NKCANT
```

Supplementary data 1 (CIR alignment)

```
PCHAS_030180   NSKIDRY-EE RLIGVDAVYD MLTRND---- ---------W FGEHYNSIKK KSDMMKLFYT YLTRLYELLK EICNTITKCN -NSPNTEECE KHAKKCVQLY
PCHAS_130070   NSKIDQY-EE RLIGVYAVYD MLTRND---- ---------W FGEHYNSIRE KTDMMKIFYT YLNRLYGLLK EICNTINTCK -DSSNTNACL NHAKKCVQLY
PCHAS_011500   CYKNNQNPNR LI-GVDDIYN MLTRNP---- ---------W FGKYYDSIKE KRGMMKIYFS YLNKLYALLK GICDAINKCD -YPSNADECI KYANKCANLY
PCHAS_146850   GYKNDQNPNR LI-GVDNVYD MLTRND---- ---------W FREHYNSIKG KKNIMKIYFS YLNNLYTLLK KICDTINKCN -DPSNNKECI DYANKCVKLY
PCHAS_070100   SYKLYQYKER SI-EVDDVYD ILTGND---- ---------W FQEHYKSIEE KKDMMKFHLI YLNNLYTLLK EICNTITNCS -DSSNSSECQ ESAKKCAASY
PCHAS_060050   SYKISQNPKI EI-AKNTMYD VLTQNE---- ---------W FNEHSESIEK IKDMMNFHFI HLKNLYEFLK GICETINKCN -GSSNTNECI ESAKKCADSY
PCHAS_030110   SSKIKENTKA GH-GINDIYN TFIKNN---- --------SW FSEHSKSIEK KKDMMNFHSI YLTNLYKFLK GICETINICN -DPSKTSECI KSANKCADSY
PCHAS_130050   SSKIKENTKA GY-GINDIYN TFIKNN---- --------SW FSELSESIEK KKDMMNIHLI CLKNLYALLK GICETINKCN -ESSNSSECI KAGKECASLY
PCHAS_030090   NYKISQDQNI EI-IRGTMYE ILKQND---- ---------W FGEHSESIEK IKDMMGIHSE YLKRLYRLLK GICETINTCN -GSSDTNKCI ESAKKCVDSY
PCHAS_146870   SYKISQNPNI EI-AKNTMHD ILTQNE---- ---------W FNEHSESIKK IKDIMGIHFL YLNKLYEFLK GICETINKCN -GSSDSNECM ESAKNCKSLY
PCHAS_130030   SYKISQNTNI EI-IRGTMYD ILKKND---- ---------W FGEHSDSIDN RKDTMEIHYL YLKNLYNFLK GICETINKCK -VSSNSSGCK ESAEKCSYLY
PCHAS_040110   NSKLSQKPDI EI-ERYTMYN ILKQYN---- ---------W FGEDGKSIEK KKDIMGIHYL YLKNLYDFLK GICETITNCN -GHSKTSECI ESAKKCSGLY
PCHAS_042030   NYKISQNQNI EI-IRGTLYN ILTQNN---- ---------W FSEYSESIEN RKDTMKIHYL YLKNLYDFLK GICETINKCK -DSPTSNECK KYGEKCSDLY
PCHAS_060140   NSKITQNSTI EI-EKYTMYN ILKQNN---- ---------W FGEHSEPIET KKEIMGIHYL YLKHLYDFLK GICETINKCK -DSSHSSDCQ ESAEKCSGLY
PCHAS_030210   SYKIRQNANI EI-EKNTMYD ILRQNG---- ---------W LSEHIESIEK IKDIMGIHFE YLNRLYRFLK GICETITNCN -DPSKTSDCQ ESAKKCSELY
PCHAS_060060   SYKISQNKNI EL-IRGTMYG ILKQNG---- ---------W FGEHSDSIEN RKDTMEIHFE YLNRLYRFLK GICETITNCK -DSSTSSKCQ DSAKKCSGLY
PCHAS_050070   SYKIRENTNI KL-IRNTIYD ILTQNE---- ---------W FSEYRQYTDK NEDIMGFHFL YFKRLYDLLK GICETINKCS -NSSNNEECI NSAKKCDEFY
PCHAS_040050   NSKIRGTAEM EA-EINNIYN VLTENG---- ---------W ISEYRQYTNK NEDIMKFHFL YLKKFYKFLK GICETITNCS -GPSKTEECI KSAKKCNEFY
PCHAS_001050   NSKIRGTAKM EA-EIKNIYE ILIEND---- ---------W ISEYRQYTDK NENELKFHFL YLKKFYALLK GICETITNCS -DSSKTEECI KSAKKCNEFY
PCHAS_083760   SSKIKENTKI DI-EKNTMYD IFTQNE---- ---------W FSEHSESINN KKDIMGLHFI YLNNLYKFLK GICDTINKCK -SSSNPTECQ ESAEKCGELY
PCHAS_140070   NYKITQNTKI E-DGINDIYN ILIKNN---- --------SW FSEHRQYTDK NENVMKIYFS YLNNLYALLK EICNTIDKCS -DPSKTSECQ ESGKKCASLY
PCHAS_000180   NSKVKVNEVF E-PVTKNIYN ILNEHN---- ---------L LSEYREYTDK NEDVMKIYYV FLKNLYEFLK GICDTINKCK -GSSTSNECQ ESGKKCVELY
PCHAS_001060   NSKVKVNEGF E-PETDNIYN ILNEHN---- ---------L LSEYREYTDK NEDVMKIYYA FLKNLYTLLK EICNTINKCK -GSSTSTECQ NSGEKCVELY
PCHAS_011450   SYKIRENPNI EF-IRSTIPG ILEKNQ---- ---------W YKELSKSADD KENTMGFHFI YLTNLYKFLK GICETINKCK -GSPTSSECK QYGEKCSGLY
PCHAS_060030   SYKIKQNPKI EF-IRTTIDD ILKKNE---- ---------W YNELNIPADD KKNMIRFHYI YFTNLYNFLK GICETINKCK -SSSSNDECI ESAKKCGELY
PCHAS_120030   SYKIKQNPKI DF-IRTTIDD ILKQNE---- ---------W YNELGITVDD KKSTIRFHYI YMNNLYNFLK GICETINKCK -VSSNSNDCQ ESAKKCVDLY
PCHAS_030120   NYKIRQNKKI EA-GINDIYD MITTNE---- ---------W FTEHRQDTDK KKDMMKFFYT YLNNLYALLK GICDTINKCS -ESSSTNECI KSAERCVSLY
PCHAS_114640   SYKIKQ-NKK ILFGVSNIYE MITHNE---- ---------W YTDYREYIDK KKNIMGLYDS YLINLYKLLK KICDTINKCN -DPSKTKECI ENAKMCATLY
PCHAS_130350   SYKLQQSNRK SSKPIGLYDF FNNHVV-NG- ------DWYE VVEEHVQPKI SLLNNNINID LMSDIYYILK EMCKKFSNKN DNFDYLEDFM NYYKRCGENI
PCHAS_030150   NHKLQQLPKS ELIDLNNFYD KYIKDN---- --------EW YASFISEIDT QKELMNVDAT MLSDLYYIFK EMCNKFSNTE NHEDFAYFYN NNNNNFVKTY
PCHAS_060120   SYKLQQLNEK NSQSIGLNDF FSNYII-NG- ------EWYD DIEEYVEPKM FLLNNDINIE HMSDIYKIFK QMCKIFSNDA DDIDDLDFTG YYEKSCGENI
PCHAS_041960   SYKLQQLNKN NDKPIGLNEF FNKHIA-KG- ------DWYE EFKEQIESKI HLVDKEIDID LMSDVYYIFK QMCGGLSNQE DDFNYLEDFS TYYKICAEKL
PCHAS_130110   SYKLQQLPPS ELIDLNQFYD NYIKNN---- --------VW FESYLSDIDT KKNLMSVDTK TLSDLYHIFK EMCKIFYNNG DDDYNLDDFT GYYINCAKNY
PCHAS_041950   CHRLYKISKD YKLPLKNSLE EYLGKS---- --------IG SFNYRGILFS KKHLMDSNIA IMNMLYLLFQ KICETINIYE AHNAKPHEYI NKGLECHAIY
PCHAS_060130   CHRLYKISKG HNLPLKHSLE KYLGKS---- --------TG NINYRGILNN KTYLMNSSIE IMNMFYLLFQ QLCEIINTYE TNKLQPYQYI NSA-AQFYII
PCHAS_130120   CHRLYKISKD HNLSLNQLYG NNLGKS---- --------RG DFNYKGILNN KIYLMNSNVA IMNMLYLLFQ KICETINTYE THNAQPHEHI NKGIEYHFMY
PCHAS_070170   SHILYKKLEN NTITLKSAHE KHLKEN---- --------FG NYNYWNLLAK KYYLTDSNIA VMNVLYLLFQ QICDTIKEYR KENVLGHEYA NKA-FQSYII
PCHAS_100060   SHILYKKLEN NTITLKSAHE KHLKEN---- --------FG NYNYWNLLGK KFYLTDSNIA VMNVLYFLFQ QICDTIKEYN KENVLGHEYA NKA-VQSYIM
PCHAS_146770   SDKLFKIEND YKATLEESYD KHLKNS---- --------MG NFNYWRVLNS KQIYKKATIR KMSAMYTLLT YICKLITEYN KHNKSKNVSE NDRTQCRNYY
PCHAS_146790   GDKLFKLEKN YKTTMEESYK KYLDNH---- --------IG NYKYWNVINS KRLYKDATIR KMNELYNLLS YICKLITEYN KNPKKNRDTL GNYAQCRNYY
PCHAS_060160   SDKLFKIEND YKATLEESYN NHLKNY---- --------MG NFNYWRVLNN KKVYKDATIW KMSAMYTLLT YICKLITEYN KNVKNDRDTL VKSSQSVNYY
PCHAS_000260   GDKLFKLEKN YKATMEESYK KHLDKH---- --------TG NYIYWNVINS KKLYKDATIR KMNELYNLLS YICKLITEYN KNIKNNRDLL GNYAQCQNYY
PCHAS_130280   GDKLFKIDND YKITMEESYE KNLKNS---- --------MG NFNYWNVLRS KNIYKNATIR KMNAMYTLLT YICKLVTEYN KNNKNKNISD NDRTQCRNYY
PCHAS_083720   GDKLFKLEKN YKATMEESYK KHLDKH---- --------TG NYIYWNVINS KKLYKDATIR KMNELYNLLS YICKLITEYN KNIKNNRDLL GNYAQCQNYY
PCHAS_090010   GDKIFKVGKD YKSTLEESYE KNLKNI---- --------TG KFEYWDALSS KEVYKDATIV RMYIFYNLLN SICKTINELN KNLNNDAESL KNYTKCLYLY
PCHAS_114600   GDKIYKVDKD YKSTLEESYE KHLKNI---- --------TG KFEYWDALNS KQVYKNATIV RMHIFYNLLN SICKTINELD KNLNNDAAIL KKYTHCLDLY
PCHAS_146860   SHLLYKMETN NLISLENSYE NNLGKS---- --------IG DFNFLNLLHN KKYLMNANIV IMNVFYHLFK EICRTIKIYQ TENTLSHEYI NSATQIYFIY
```

Supplementary data 1 (CIR alignment)

PCHAS_130090	SNILYKIAPT	HLESLGESYE	KHLSKS----	--------VG	NFVFWNLLYN	KRHLFDGNIS	ILSIFYILFK	QMCETFDIYN	KRGISGHEYA	INAAQSYIIY
PCHAS_030170	SHILYRKLED	HTITLKSAYK	KHLKDN----	--------FG	EFRHWYLLQD	KKYLTNSNMA	IMNLIYLFFQ	QICDTIKIYK	KPHVLGHEYA	SKAFECYFIY
PCHAS_130060	GHILYRMTED	HAITLKSGYD	KYLKDN----	--------FG	EFRHWYLLQD	KKYLTNSNMA	IMNLLYLLFQ	QFFEMIKKYR	TPHVLGHEYG	NMALECYFIY
PCHAS_030200	SNKLYEIATN	NSKYLNQSYE	INLDKS----	--------IG	NFDFLNSRDD	TKELKDANIT	IMNILYLLFK	EICKAVWMDQ	GKSTEMYKHT	TQITQCFIIY
PCHAS_060080	SNKLHEIATN	NYKYLNQPYE	NNLDKS----	--------IG	NFDFLNSRDD	TKELKDANIT	IMNILYLLFK	EICKTVWMDQ	GKNTKIYEHT	TQITQCFLIY
PCHAS_060110	ADKFRKANNE	GFFTLDEYYE	EFLVNH----	--------ND	NFNYWYELDN	KMHLKDSNII	LMVEFYYLFT	NICNMLLESE	KSELDLNKIK	VFDNNCFRKY
PCHAS_030140	ADKFLKVNND	YSFSLNDYYE	ELIVKP----	--------GD	NFSWWEKLDD	KMLWKDSNII	LMARFYYLLM	DICNALLENE	KSEFDLKRIE	GIDKKCHITY
PCHAS_130100	ADKFLKVNDD	YSFSLNDYYE	ELIVKH----	--------GG	NFGWWEKLDD	KMFWKDSNII	LMARFYYLLM	DICNALLENE	KSELDLKNIE	ENDKKCYLNY
PCHAS_041990	ADKFRKANNE	GFFTLDEYYE	EFLVNH----	--------ND	NFNYWYELDN	KMHLKDSNII	LMVEFYYLFT	NICNMLLESE	KSELDLNKIK	VFDHNCFRKY
PCHAS_130080	ADKFLKISPN	RSFSLYDYYE	KVIVKR----	--------GE	NFNCWGKLDN	KKDLKDSNLS	IMTLFYQIFM	NICNAIMKNE	ISRLELNKFM	DIDHSYHQLY
PCHAS_030190	ADKFLKISPD	RSFSLNDYYE	KVIVKQ----	--------GE	NFNCWEKLDN	KKDFKDSNLS	IMSLFYQLFM	NICSALMKNE	ISNFELKKFK	DIDYDYYQIF
PCHAS_060090	ADKFLKISPD	RSFSLNDYYE	KVIVKQ----	--------GE	NFNCWEKLDN	KKDFKDSNLS	IMSLFYQLFM	NICSALMKNE	ISNFELKKFK	DIDYDYYQIF
PCHAS_041970	ADKFLKITPD	HSYTLNEYYD	EVIANN----	--------DG	NFNCWGKFDN	KKHFKYSNIS	IMSVFYQLFM	NICNAVVEND	ISKLETKKFM	MIDHNYCQIY
PCHAS_041980	AAKFLNITHD	VSFSLNDYYE	KFIVNQ----	--------GG	HFNCWGKLDN	KEYLKDSNLS	IMSVFYQLFM	NICKAVVENE	ISKLETKKFM	MIDYNYYQIY
PCHAS_042020	GYRLFQTPSY	SSSTLVDYYN	NYILKS----	--------HL	PFKNDYLIDK	KKHLLHANFE	LINKLYKLLN	YLCNIITEPN	-IYTESTKIK	SNISMFQDEF
PCHAS_060070	GYKLFQTGSY	NSPNLIDFYN	NHLTKS----	--------IL	PSGLNYILEK	KKHLLYANLE	LINELYILFN	DMCNIITDPN	-IYTESDKIK	SNVSKFQDTF
PCHAS_011330	SNILLQISKD	HNSILEASYK	TYLEKH----	--------MG	NYNYWNILGN	KRYLKDGNIS	YMSQFYKLFE	QICNIINEYN	TNGPTSKNLG	KISIECSKTY
PCHAS_000220	SNILLQISKD	HNSILEDSYK	AYLEKH----	--------MG	NYNYWNILGN	KQYLKVSNIS	YMSQFYKLFD	QICNTINEYN	TNGAGSKNLR	NISMECSKTY
PCHAS_042070	SDKLYKLEEN	KIRTLDQFYE	KHLKDY----	--------TG	NFKYWNVIDS	RKVYKGANTW	YLSELYSLLN	DICSIVIEYN	KNKNKKNKIE	KASSQCHKKF
PCHAS_110030	SGKLYKLENN	KSTTLEESYK	KYLENN----	--------MP	SFNYWNVLGS	KREYKIANVW	YMSRLYSLLE	CICSIVIEYG	KSKRN-KQIE	QISQQCYQKF
PCHAS_140140	GDKSYKLDKN	KTATLEDSYK	DNLEKY----	--------TG	NFKYWNVIDS	KRVYKRANIW	YMSELYKLLK	SICSIVIEYN	KNKSK-KKIE	QISSECYQKF
PCHAS_010040	SDKLYKLEKN	KGVTLEESYK	NYLDAH----	--------TG	NFKYWNIVDS	KRVYKKANVW	YMSELYGLLK	YICDLVNEYN	-KKPNKEKIE	GISRKCQQKF
PCHAS_073150	SDKLYKLEKN	NAATLEEYYN	KHLKEA----	--------MG	NYNYWNLLGG	KQEYKRANVW	YMSELYSLLN	DICSIVIEYG	-KNKRSKKIE	HISPQCYKKF
PCHAS_073130	GDKLFKMHNK	SKGKGRNNNI	TLYSAY-EKY	LNNH----RG	YLDYWALLNN	ISGLKEANLE	HMHKFYRLLN	DICKTIVYYI	HKDSKNNNLI	MNSTECSNQY
PCHAS_040060	SDKLFKMYQK	DKRKGENNRI	TLDEAY-KKY	LDKD----MG	DYKYWNRLDN	VKGLKDANLS	HMNEFYKLLS	HICKTIIYYK	IKDAKPTNLH	QNSTNSSNQY
PCHAS_000430	SDKLFNMYQK	DKRKGENNRI	TLDEAY-KKY	LDKD----IG	DYKYWNLLGN	INGLKEANLS	HMNEFYKLLK	HICKTIMHHK	IKPTESASLI	QNSTNSSNQY
PCHAS_104200	SDKLFKMHEE	GKKKGQSNIT	TLDEAY-KSY	LDKN----IG	NNKYWDALDN	VKGLKDANLS	HMNEFYKLLK	HICKTIMHHK	NKPTESENIL	QNSTNSYNQY
PCHAS_114720	SDKLFKMHED	GKKKSQSNIT	TLDEAY-KSY	LDKN----IG	NNKYWDALDN	VKGLKDANLS	HMNEFYKLLK	HICKTIMHHK	IKPTEYTSHL	ENSTNSSNQY
PCHAS_000770	SDKLFKMYYE	SKGKDAKKGF	VYNITL-NQA	YDKYLKKHEV	KLNYWTLLDI	IPGLKEANLK	YMSEFYKLLS	NICKTIKEYN	DKGAESKKLS	KYSGSCLNQY
PCHAS_130220	SDKLLKMYYD	SKGKDVKKGF	VYLITL-NQA	YDKYLKKNKV	KLDYWDLFGH	INGLKEANLK	YMSEFYKLLN	NICKTIADYK	DNGAGSKQLS	KYSKNCLNQY
PCHAS_070040	SDKLFNMHKE	GKGKHIKKGY	MDTFTL-KQA	YEEYLEKHKG	IFGYWYLLDM	IKDLKEAYLK	YMSEFYKLLN	NICITIKEYN	DKGAKSSQLS	KYSKNCLNQY
PCHAS_011490	SDKLYKMHLK	SIGQKDTAEY	MDGTTL-NQA	YDNYLKNYKV	GLGYWDLLDM	IMGLKEANLK	YMAEFYKLLN	NICKIITDYN	DNGSESKKLS	KYSENCLNQY
PCHAS_070060	SDKLYKMHLK	SIGQKDTAEY	MDGTTL-NQA	YDNYLKNYKV	GLGYWDLLDM	IMGLKEANLK	YMAEFYKLLN	NICKIITDYN	DNGSESTKLS	KYSENCLNQY
PCHAS_030070	SDKLFKIHDK	SEEK----D-	-KKITL-NQA	YEEYLEKHKG	IFDYWIFLNM	QQGLKEANLR	YMSEFYKLLD	KICKTITDYE	KKRDEITNHI	TNSTECSNQY
PCHAS_137110	SDKLFKI-HD	ESK---DKDN	--EITL-NQA	YDTYLKNHKV	NFNYWNFFYN	IESLKESNLW	YMSEFYKLLD	KICKTITDYE	KNRDEITNLI	TNSTECSNQY
PCHAS_114700	SDKLFKMDDE	GKEKNSKKPN	IHTITL-NQA	YEKYLKNHKV	KLDYWALLNI	MPGLKNANLK	YMSEFYKLLN	HICKVITYYN	EKGAKSRKLS	KYFVDCRRQY
PCHAS_137030	SDKLFKMDDE	GKEKNSKKPN	IHTITL-NQA	YEKYLKNHKV	KLDYWALLNI	MPGLKNANLK	YMSEFYKLLN	HICKVITYYN	EKGAKSKKLS	KYFVDCRRQY
PCHAS_011480	SDKLFKIHKE	AKNIGEGRM-	-DGTTL-NQA	YDTYLKDHKV	GLDYWVLLDM	IKGLKEANLK	YMSEFYKLLN	IICKTIVDYK	DNGTESKKLS	KNSISCRRQY
PCHAS_050040	SDKLFKIHSK	SENK------	NKQITL-NQA	YDMYLKKHKA	KLDHWILFDN	IQGLKNANLK	YMSEFYKLLN	KICITITDYS	ENGTESKNII	MNSTECSNQY
PCHAS_000470	SDKLYKMHIE	SIGQKYENNY	MDGTTL-NEA	YEKYLKNYKV	KLDYWILFDM	IKDLKEANLK	YMSEFYKLLN	KICKIITDYN	-NSAQTKQLS	KYSVDSRRQY
PCHAS_000170	SDKLFKIHKE	AKNIQRG--Y	MDATTL-NQA	YKEYLEKHKQ	RLDYWNILNM	QQGLKEANLK	YMSEFYKLLN	HICKIITYDE	TKDAKSREFS	INSIGCSRQY
PCHAS_130170	SDKLFKMHLK	SIDKKDVNNY	MDGTTL-NEA	YKNYLEKYKG	IFDYWAFLDM	IKGLKEANLK	YMSEYYKLLN	LICKIITGYY	-NGTQTKQFY	KYPADCSHQY
PCHAS_000660	SDKLLKIHKK	GKGKKIGIGR	MDDFTL-KRA	YEEYLKNHRQ	RLDYWSLLDM	NPGLKEANLW	YMSGFYKLLN	LICKIITGYY	-NGPKNKQFY	KYPADCSHQY
PCHAS_040080	SDKLLKIHKK	GKGKKIGIGR	MDDFTL-KRA	YEEYLKNHKQ	RLDYWYLLDM	IKDLKEANLW	YMSEYYKLLN	LICKLITDYY	-NGVQTKQLY	KYPADCSHQY
PCHAS_070070	SDKLFKMHLE	SKGIKDKPDY	MDGITL-NKA	YKNYLEKHKG	ILDYWTHLDM	IPSLKEANLK	YMSEFYKLLN	LICKIITGYN	-NGSQNKKLY	KYPSDCSFQY
PCHAS_137090	SDKLFKMDDE	SK---DPNRN	IYTITL-NQA	YEKHLKKYKV	KLDYWTLFDI	IGDLKNANLK	YISEFYKLLS	NICKAIADYE	KNGAKSKKLS	KNSADCSYQY

Supplementary data 1 (CIR alignment)

```
                  ....|....| ....|....| ....|....| ....|....| ....|....| ....|....| ....|....| ....|....| ....|....| ....|....|
                       205        215        225        235        245        255        265        275        285        295
PCHAS_000950      KELVNKVFED EHYCNALLNL KKAYEKFTNG --------NN T--NAFPKIN EI-------- ------EKMN NCKKLCEKAN RSWKVIHVEV KDVVDAKKVS
PCHAS_001010      KELVNKFFED ENYCNVLLTL KKAYENFRSG -------SNN T--STLPILN EI-------- ------EGIS NCNKLCEKAN RSWKLIHVEV KDVVEEKKVS
PCHAS_140090      EKLNENDTKG VSYKKILSTL FNDYDRLRKK ---------- C--NNFQSFP SM-------- ---KITQSYV QD-------- ---------- ----------
PCHAS_000730      EKINSNITGN DSYSQMLSNL FVDYDNLKNK ---------- C--KNSSSLP TI-------- ---KKPQNFE QR-------- ---------- ----------
PCHAS_070160      QKLND-ITEN DSYSQILYTL FNDYNSFKND --CAK-HCSK C--IDIPTLP DI-------- ---NLPQNFV QDKVES---- ---------- ----------
PCHAS_030020      NQFNE-VIGN QSYRKILTIL FNDYDNLKNK ---------- C--KDSSSLP TI-------- ---EQIQYSV KGSEKG---- ---------- ----------
PCHAS_000360      QKLND-ITGN NSYSQILYSL STDYKNLKND --CAK-KCTG C--KDIPTLP NI-------- ---KTPQNSI ECSAQIRVQD KGESPGQG-- ----------
PCHAS_110020      EDLNQ-ITEN ISYREILSSL SIDYDDFKNE --CAK-NCSY C--NDIPTLS EI-------- ---------- ---------- ---------- ----------
PCHAS_050060      EGLNQ-ITGN SSYREILYNL FNDYDDFKNS --RAK-NCSS C--NDLPTLP KI-------- ---------- ---------- ---------- ----------
PCHAS_010030      GTLNN-HKKG SFYSQILSTL SKDYNKLKEE --CDN-GQSK K----FPSLP PI-------- ---KPTKSYT HTYGETDIQF ---------- ----------
PCHAS_030030      NKLND-ITEN NLYSQILYAL FNDYNSFKNG --CAK-NCSS C--NDVPTLS EI-------- ---------- ---------- ---------- ----------
PCHAS_000350      EGLNQ-ITGN SSYREILYNL FNDYDHLKTD --CAE-KCTD C--KDIPTLS EV-------- ---------- ---------- ---------- ----------
PCHAS_011530      ENLNQ-IIGN NSYREILLSL STDYNKFKNG --CGK-KCID C--KDMPTLS EI-------- ---KTPQSF- ---------- ---------- ----------
PCHAS_073160      EELNQ-ITGN NSYREILSSL LTNYNNFKSD --CNK-KCSK C--SDIPTLT NI-------- ---KAPQSF- ---------- ---------- ----------
PCHAS_070090      ESLNQ-ITGN NLYREILFSL SNDYDDFKNY --CAK-NCSF C--NNLPTLS EI-------- ---------- ---------- ---------- ----------
PCHAS_000580      ENLNN-ITGN DSYREILFSL STNYNNFKND --CAK-KCSK C--SEIPTLT DI-------- ---KVPKSYV QDQVES---- ---------- ----------
PCHAS_140130      NELNNAITKD SLYYQVLSTL SNDYDNFIKK ------RGAV C--VDFPPLS AY-------- ---------- ---------- ---------- ----------
PCHAS_140040      KILLNNDIEG SLYCQILSIL STDYYNFISQ --CYE-KKVG C--DKFPSLP DC-------- ---------- ---------- ---------- ----------
PCHAS_000740      KILLNNDIEG SLYCQILSIL STDYYNFISQ --CYE-KKVG C--DKFPSLP DC-------- ---------- ---------- ---------- ----------
PCHAS_100030      EILFNNDTEG SLYSQILSIL STDYYNFINL --CYE-KKEG C--GRFPCLP PY-------- ---------- ---------- ---------- ----------
PCHAS_001120      EILFNDDTED SLYSQILSIL STDYHNFINQ --CYE-KKKG C--GRFPSLP DC-------- ---------- ---------- ---------- ----------
PCHAS_011520      EILFNNDTEG SLYSQILSIL STDYYNFINL --CYE-KKEG C--GRFPLLT VY-------- ---------- ---------- ---------- ----------
PCHAS_070030      EILFNKDTED SLYSQILSIL STDYYNFINL --CYE-KKEG C--YRFPLLP VY-------- ---------- ---------- ---------- ----------
PCHAS_073190      ETLFNDDTEG SLYSQILSIL STDYYNFINQ --CYE-IKEG C--GRFPCLP PY-------- ---------- ---------- ---------- ----------
PCHAS_000570      EILLNNGIED SLYSQILSIL STDYYNFINQ --CYK-KKVG C--GYFPSLP VY-------- ---------- ---------- ---------- ----------
PCHAS_114730      EMLRNNGIEG SLYSQILSIL STDYYNFVKK --CYE-TKEG C--GRFPCIP PY-------- ---------- ---------- ---------- ----------
PCHAS_010020      QNLLNDGADG SSYRQMLSTL STDYDNFKKY --CDE-KCTG C--SSIPLLP TT-------- ---KTTHFPA HI-------- ---------- ----------
PCHAS_000280      EKHNE-INKD SLYSQILSTL LTDYNNFKSY --FVE-KCSH C--RDISAFP VI-------- ---------- ---------- ---------- ----------
PCHAS_000490      EKHNE-INKD SLYSQILSTL LTDYNNFKSY --FVE-KCSH C--RDISAFP VI-------- ---------- ---------- ---------- ----------
PCHAS_000030      QNLLNNNTKD SPYSQILSTL STDYTNFKSY --CAE-KCNG C--DNIPPLS YI-------- ----EPNPFV QN-------- ---------- ----------
PCHAS_000090      QNLLNNNTKD SPYSQILSTL STDYTNFKSY --CAE-KCNG C--DNIPPLS YI-------- ----EPNPFV QN-------- ---------- ----------
PCHAS_000270      QNLLNNNTKD SPYSQILSTL STDYTNFKSY --CAE-KCNG C--DNIPPLS YI-------- ----EANPFV QN-------- ---------- ----------
PCHAS_000120      QNLLNN-NKD SSYSQILSTL STDYINFKSY --CDE-KCGV C--KEIPSNL DA-------- ---VTSQNSM GSSGLSHVQD REENPVVI-- ----------
PCHAS_001090      EKLNG-NTEN SSYRKVLYSL STEYNNLKNK ---------- C--SVFQPLS DI-------- ---KTTQSFV EGSLES---- ---------- ----------
PCHAS_011510      EDLNK-DEEN SSYNKLLSTL SNDYDNLKNI ------FKNK C--PNLQPLP EL-------- ---TPKKISV ETSTPSHVQD NTHSSLQG-- ----------
PCHAS_070050      EDLIK-NKEK SLYTQILSTL SDDYKNFIKI --YHN-KDKS C---NFQTLP EL-------- ---TPQKISV EN-------- ---------- ----------
PCHAS_000720      KDLIK-NKEN SPFSQILSTL LDDYNNLINK ------YGNK C--SIPQSIS QL-------- ---TPQKISV ESSLQ----- ---------- ----------
PCHAS_070020      EELNE-NKGK SLHTQILSTL LDDYNNLINK ------YGNK C--SIFQSFP EL-------- ---TPKNKPV ESSLQT---- ---------- ----------
PCHAS_104260      EELNE-NKEK SLHTQILSTL LDDYNNLINK ------YGNK C--SISQSIS QL-------- ---TAQNKPV ESSLQT---- ---------- ----------
PCHAS_140020      EELNE-IKEK SLHTQILSTL LDDYNNLINK ------YGNK C--SIFQSFP EL-------- ---TPKNKHV ESSLQT---- ---------- ----------
PCHAS_030060      KNLNE-HIID SSFSQILSTL LNDHNNLINK -YCKG--SCS -----FSSIS QL-------- ---TPQKKTV DNSVKDSGET LGDT------ ----------
PCHAS_000750      KELNN-NIED SSFSQILSTL LNDHNNLINK ------YCKE S--CSFSSIS QL-------- ---PPKKKPV EGSAKGGEQT SLPI------ ----------
PCHAS_000680      FEKLNNNKEN SLYTQILSTL SNDYKNLKKK ---------- C--PQFQSIS EI-------- ---KRSQSSA ENSVKVSEQP TVLI------ ----------
```

Supplementary data 1 (CIR alignment)

```
PCHAS_000420   NDLNN-NKEN SSYNKLLSIL SDDYNNLQKK ---------- C--TNFPFLP VY-------- ---------- ---------- ---------- ----------
PCHAS_000100   EDLNN-NKEN SSYNKLLSIL SDDYNNLQKK ---------- C--TNFPSLP VY-------- ---------- ---------- ---------- ----------
PCHAS_000310   NDLNN-NKEN SSYNKLLSIL SDDYNNLQKK ---------- C--TNFPFLP VY-------- ---------- ---------- ---------- ----------
PCHAS_040040   KDLNN-NKEN SPFSQILYTL SDDYNNLQKK ---------- C--TNFPSLP VY-------- ---------- ---------- ---------- ----------
PCHAS_120060   EILFNNDTED SPFSQILYTL SDDYNNLQKK ---------- C--TNFPSLP VY-------- ---------- ---------- ---------- ----------
PCHAS_104230   EVLLNNGIDD SLYSQILSIL STDYNNLRKK ---------- C--TNFPSLP VY-------- ---------- ---------- ---------- ----------
PCHAS_030040   EVLLNNGIDD SLYSQILSIL STDYNNLQKK ---------- C--TNFPSLP VY-------- ---------- ---------- ---------- ----------
PCHAS_000340   EILLNNGIDD SLYSQILSIL STDYNNLQKK ---------- C--TNFPSLP VY-------- ---------- ---------- ---------- ----------
PCHAS_001110   DKLNE-ITGN NSYKKILTTL FDDYGNLKKK --CDD---DP S--SDFPTLS PI-------- ---KTTQSF- ---------- ---------- ----------
PCHAS_120040   EEFNK-ITVN NSYSKMLSIL SNDYNCLKDH --YAN-NCSE C--SNIPDFP EI-------N PPKGSPHNSL EKSGENRAQD NVDGSVHSSL QGHAGNSQHV
PCHAS_000130   EELNK-IARN DSYSKILSTL SNDYNYLKNH --YAT-NCSG C--SNIPNFP EI-------- ---KTPHFSV ETSTPSHVQD NPDSSLQG-- ----------
PCHAS_000400   EKLNK-ITKN ESYSKILSTL SDDYNCLKDH --YDN-NCNG C--TNIPNFP EI-------- ---KTSQISV EGSTPSHVQD NPDSSLQG-- ----------
PCHAS_040020   EKLNK-ITKN ESYSKILSTL SDDYNCLKDH --YDN-NCNG C--TNIPNFP EI-------- ---KTSQISV EGSTPSHVQD NPDSSLQG-- ----------
PCHAS_000110   NKLNE-TNQS DSYKNVLYSL STDYNNLKKY --LIE-NCTD C--NDTSSFP EI-------- ---KAPQGSF QI-------- ---------- ----------
PCHAS_120050   QKFLNDDTDD SSYKQILPIL SNGYDNLIKK --CNN-GQHS N----FPPLP TT-------- ---KTTQLSA HI-------- ---------- ----------
PCHAS_104250   QKFLNDDTNG SSYKQILPIL SNGYDNFKKK --CNN-TQSS N----FPLLP TT-------- ---KTTQNVV EI-------- ---------- ----------
PCHAS_000320   QKFLNDDTNG SSYKQILPIL SNGYDNFKKK --C---NNTQ S--SNFPLLP TT-------- ---KTTQNVV EI-------- ---------- ----------
PCHAS_000410   QTFLNNDIDD SSYKQILPIL SNCYDNFKKK --CNN-TQSS N----FPLLP TT-------- ---KTTQNVV EI-------- ---------- ----------
PCHAS_140030   NELNE-NNQS NSYKNVLYSL STDYNNLKKY --RSE-VCGS C---YDASFP DI-------- ---EAPQGSF QI-------- ---------- ----------
PCHAS_114740   NKLNG-KNES DPYKNVLYSL STDYDNFKKY --CAE-NCTD C--NDTSSFP EI-------- ---KAPQGSF QI-------- ---------- ----------
PCHAS_040030   NELNN-ITRN ISYSQILSNL FNDYNSFKSY --RAE-KCSK C--SNIPSLP DI-------- ---KTAQFSG QDNAEILVDI ---------- ----------
PCHAS_070130   EKLNG-ITGN SSYRHILYTL SADYNSLKSD --CAE-KCTD C--KDIPILS EI-------- ---------- ---------- ---------- ----------
PCHAS_073180   EKLNG-ITGN NSYRKILYTL STDYDDFKNY --FVE-KCSG Y--TNLPTLS KI-------- ---------- ---------- ---------- ----------
PCHAS_000300   EKLNG-ITGN SSYRKILYTL STDYDDLKND --FAE-KCSG Y--SDIPTLS AI-------- ---------- ---------- ---------- ----------
PCHAS_120070   EKLNG-ITGN NSYRKILYTL STDYDDFKNY --FVE-KCSG Y--TNLPTLS KI-------- ---------- ---------- ---------- ----------
PCHAS_001130   EKLNG-INEN SSYRKILYTL STDYDDFKNY --FVE-KCSG Y--TDLPTLS KI-------- ---------- ---------- ---------- ----------
PCHAS_100040   EKLNG-INES SSYRKILYTL STDYDDFKNY --FVK-KCSG Y--TDLPPLS KI-------- ---------- ---------- ---------- ----------
PCHAS_000560   EKLNG-INEN SSYRKILYTL STDYDDFKNY --FVE-KCSD Y--TYLPTLS KI-------- ---------- ---------- ---------- ----------
PCHAS_050020   EILND-HIKD SPYSQILSTL SNDYENLKKN --CND-AQSS N----FPPLP PI-------- ---KSTKKSA QYSLESSGLT SMQS------ ----------
PCHAS_000020   KELND-HIDG SSYSQILLTL SKDYDNLKNK --CDN---GQ S--SNFPSLP PI-------- ---KPTKSST QNNIEASVQL ---------- ----------
PCHAS_000290   EILND-HIKD SPYSQILLTL SKDYDNFKNC --CNK-KKGE S--CDFPSLP QI-------- ---SPKKSFA QNSLESPGHT SGHN------ ----------
PCHAS_060020   KELND-HIEG SSYSQILSTL SNDYDNFKKT --CCN-GQSK N----LPSLP QI-------- ---KPTKISS QKSLEGSGKG SAQKSLESPG HTYGQN----
PCHAS_001040   KELNE-VTKD SPYYQVWCTL STDYDNLKNE --CIK-NCKE C--TELPTLP EI-------- ---KESKKYV QDYAELSAQG ---------- ----------
PCHAS_114750   KDLND-VTKD SPYYQVWCTL STDYDNLKNE --CIK-NCKE C--TELPTLP EI-------- ---KESKKYV QDYAELSAQG ---------- ----------
PCHAS_000140   KDLND-VTKD SPYYQVWCTL STDYDNLKNE --CIK-NCKE C--TELPTLP EI-------- ---KESKKYV QDYAELSAQG ---------- ----------
PCHAS_000070   KDLNN-VTKD SPYYQVWSTL STDYNNLKKK --CIN----D Q--SSFPTLP EI-------- ---KEAKKSI QGYAESSGQF ---------- ----------
PCHAS_030080   KKLNE-VAKD SPYYQVWLTL SNDYDNLKKK -------CGN CQSINLPTLP SI-------- ---KLKKISP PNSVESYGKT PIDNSVHI-- ----------
PCHAS_073200   KKLNG-IDKD SPYYQVLYIL SNDYNNLKDK ---------- C--SEFQSLP PI-------- ---KIAHSSV KGHPDNSVHI ---------- ----------
PCHAS_120020   EKLNR-ITED DPYYKVLFTL SDDYVNLKNK --CDN---GQ S--SNFPSLP KI-------- ---TPKKRSV QNFAQS---- ---------- ----------
PCHAS_000060   EKLNR-ITED DPYYKVLCTL SNDYEKLKNK --YDN---AE S--SKFPSLP PI-------- ---KPTKSST RNTVEASGQL ---------- ----------
PCHAS_130020   EDLNK-ITKN NHYYQVWNIL STGYDNFKKK --CVN-DQSS F--PSLPPLP TI-------- -------EQI QLPAKCSEEL SIQPTVLS-- ----------
PCHAS_000150   EDLSK-ITKD GYYYKVLHTL STGYDNFKKK --CDN----- -YQSSFPLLS TI-------- -------DQI EFPAKCSEEQ SVQSTVIS-- ----------
PCHAS_000500   KDLVTNGKMC TSYCDVLSTL KNSYDKFREE --IDD-DPEH K-------LP EF-------- ----IEEGIQ NCKNLCKSNE QKLENEKARS DILETVMDIP
PCHAS_001100   KNLVTNGKVC DSYCEVLSTL KNGYDKFREE --KNE-STPN F------QLP KF-------- ----IEDGVE TCKSLCKSND QKLEAENLIA EESEIFTTHQ
PCHAS_042010   TSLFNHINTC DSYLSLLNNL KAAFEQYKKS --LVL-SVSN S---PRKRIN E--------- ---GSVLGFD -CKE-CKQVH IKADEKSPKL GLKALQHEKS
PCHAS_000040   QNLVTESKMC DSYCYVLSTL KDAYDKFRRE ---NE-FDSD Y------QLP EF-------- ---TLPEGIK SCEKLCEKKN QELKVESSKI DVSETVTPTE
PCHAS_000390   YKDLVSKVKN CDSYCNVLST LKNAYDKFRE E--KIMHDPE C------KLP EF-------- ---NVEE-IE SCESLCKKKS QEPAIKNPIA KESEIDTPPK
```

Supplementary data 1 (CIR alignment)

PCHAS_030180	QGFVKEYGYC	NPYCRILSNL	KKDYENFRKN	----------	---YTPNNLP	EL--------	---NLPNGIP	SCESLCKNKE	QETNTKKPIT	EVSEIATPQK
PCHAS_130070	QGFVKEHDYC	NPYCRILSNL	KKDYENFRKN	----------	---YTTNNLP	EL--------	---NLPNGIT	SCESLCKNKE	QEANAKKPIT	DVSEIDTLQK
PCHAS_011500	QEYAKTHEYC	NPYCNVLSNL	KSDYDKFRET	------NNNK	N---DLPELK	LL--------	------DGGE	SCESFCKSKR	QKLNAEKAKP	EDSKIVTSPT
PCHAS_146850	QEFAKTHEYC	HPYCSALSNL	KNDYEKFRKK	----------	---NNNKDLP	EM--------	---ALEDGAK	NCESLCRSKE	PKLDIEELET	EDSEMDIFTE
PCHAS_070100	RSCMISTEIC	DPYCRILSNL	KNDYDKIRGN	----------	-----NSKLP	EL--------	---KPPEGRK	SCEIFCEEKK	QKLKAEKAKV	EVSEMNIRTE
PCHAS_060050	RTCLNSGELC	NPYCSVLSNL	KKDYEKFRKN	----------	-----HNNLP	KL--------	---EPPKGRE	NCENYCKSLT	QILNAEKSAI	EQA-------
PCHAS_030110	RTCMNSGELC	SPYCSVLSNL	KKDYENFREA	------NINK	K------DLP	EL--------	---KPPEGRE	NCENYCKSLT	QILNGKESAI	GGT-------
PCHAS_130050	RTCLTSRGLC	NPYCSVLSNL	KKDYEKIREN	----------	-----NDKLP	EL--------	---TPPSGRE	SCENYCEILT	QKLNAEGSAI	EGT-------
PCHAS_030090	HSCIIQKEIC	NPYCRVLSNL	KKDYDKFREA	------NNNK	D------QLP	TF--------	---TIPEGAK	SCEETCAKKW	QEPEAKEPAI	DVSEIGTP--
PCHAS_146870	RQCIIKIEIC	NPYCSVLSNL	KKDYEKIREN	----------	-----YKNLP	EL--------	---ELTEGLS	DCYGECSKQE	ERHNAGVATQ	NRLGGGSE--
PCHAS_130030	RACLLHREIC	NPYCSVLTNL	KNDYDKLKAK	----------	------YNLP	EL--------	---TLPQGLS	DCNHECFKQE	ERYKARVAAE	NHLGYGSE--
PCHAS_040110	RACILHREIC	NPYCNVLTNL	KNDYDKIREN	----------	---NRDKKLP	EL--------	---TLPEGLY	DCKTECYKQE	EGHKARVAAL	NRSSDGS---
PCHAS_042030	RACILHREIC	NPYCSVLTNL	KNDYDKLKEK	----------	------YKLP	EL--------	---KLPEGLY	DCKTECHNQE	ERHKASVAVQ	NRSSDGSEID
PCHAS_060140	RACILHREIC	NPYCSVLTNL	KNDYDKIKEK	----------	------YNLP	EL--------	---KLPQGLS	DCNHECFKQE	EQYQARVGAQ	NHSSDGSEIG
PCHAS_030210	RACIVQREIC	NPYCSVLTNL	KNDYDKFRKN	----------	---NSDKDIP	EL--------	---ELTEGLS	ECNVECSKQE	KRHKGGGAAQ	NRSSDGSEID
PCHAS_060060	RACLMQREIC	NPYCSVLSNL	KKDYDKLREN	----------	-----NKNIP	EL--------	---ELTEGLS	DCYGECSKQE	KRYEEWLAAQ	KHLSGGSEIV
PCHAS_050070	RICILSKEIC	NPYCSVLSNL	KNDYDKLRKN	---------N	D------QLP	EL--------	---TLPKGVE	SCENLCNGKQ	KWEFAKLKFE	VSKIDTP---
PCHAS_040050	RACILSEEIC	NPYCNLLSNL	KKDYDKIREK	-------YKT	K------EIP	EL--------	---TLPVGVE	SCESLCNGKQ	QWKVEKLKNE	ELKTSTL---
PCHAS_001050	RACILSKEIC	NPYCNLLSNL	KNDYDKIKEK	----------	---YKTYELP	EL--------	---TIPDGVE	SCESLCNGKQ	QWEVEELKIK	ELKTSTL---
PCHAS_083760	RTCLITKEIC	NPYCSVLSNL	KKDYDKFRKN	----------	---HNDKNLP	EL--------	---KPPEGRE	SCESFCKSKQ	ESKVEEPTNE	VSEIVTP---
PCHAS_140070	RSCIISKEIC	NPYCSVLSNL	KKDYDKFRKN	----------	---HNDKNLP	EL--------	---KPPEGRE	SCESFCKSKQ	ESKVEEPTNE	VSEIVTP---
PCHAS_000180	NTCLIQKEIC	NPYCSVLSNL	KNDYDKLRKK	----------	-----YVKLP	EL--------	---KLKEGLL	ECNAECSKKN	IQYKDIVDAQ	NNSSGGSKIV
PCHAS_001060	NICHVTKEFC	NPYCRVLSNL	KNDYDNLRKN	----------	-----NDHLP	EL--------	---KPPPGVK	SCEETCAQKC	KEPEAKEPAT	EVSKIVTP--
PCHAS_011450	RACLMHKEIC	NPYCSVLSNL	KNDYEKIREK	----------	------YSLP	EL--------	---NLPQGLS	DCNAECSKKA	SARPNGLPG-	----------
PCHAS_060030	RSCLIAKEIC	NPYCNVLTNL	KNDYDKIRKK	----------	-----YSNLP	EL--------	---ELKEGLL	ECNEECSKKA	AT--------	----------
PCHAS_120030	RACIMQREVC	NPYCRVLSNL	KKDYEKIRGK	----------	-----YNNIP	EL--------	---KLTEGLF	ECNVECSKKN	IQYKDIVDAQ	NNSSGGSKIV
PCHAS_030120	QYCVNNKRNY	NLYCSVLSHL	KSDYDKFREN	---------H	N------NLP	EM--------	---KLPNGIE	SCDSLCEKKK	QESESKKQTS	EQPEQVTTSE
PCHAS_114640	QHCADNKRDC	SPYCNVLSNL	KSDYDKFRET	------NHNK	N------DLP	EL--------	---KLPDGGE	SCESYCKSKG	QKLNAEDQVT	EEQKSVTPTE
PCHAS_130350	LKKKSNDNID	EIYSDLYDML	KNVYNDYRKY	--YYE-KNPQ	D--NAPPELP	KI--------	---EEIKKNF	TPDLGASDLQ	DSTEGSPEPQ	DGDGNSEQQQ
PCHAS_030150	EKLVTQSTMC	SSYYTLLNDF	KSSYEYFKDG	--HKD-----	---INLPELS	CL--------	---KDIKEIS	KPNLEISDSQ	DSTDGNPESQ	DGGSENSNGT
PCHAS_060120	LKKNSNDTRD	SHCRALYDML	EHVYNDFKKD	-YVEV-MGPQ	T--NELPEIP	KI--------	---EEIKEII	ESKLDESDSQ	DDVPNSEHIN	STNDLEPQQN
PCHAS_041960	KKSSNNDISD	DQYLAIYDML	HHVYADFRKD	--YLK-NNPE	C--DKIPDLP	NF--------	---KEIEVTP	EPDLDEPDLD	ELDPQENTNE	DPETQDGDVD
PCHAS_130110	SKQCNNTSGN	-TNFVLYDML	KHVYDDFKKG	--YYK-NNPQ	A--NSFPEIP	EI--------	------EEIK	EPLISDSENL	DSLDSTDENI	ETQDGGSENS
PCHAS_041950	DNLSKSVNQC	GPYLKLLEHL	KTIYNAFIKD	-AIKK-NSHD	KICDQLIEFP	SI--------	--DTKKFGSE	FKRTECKKLH	QMLEKNVPML	KDESQEEHDE
PCHAS_060130	YNKFSNVNQC	GPYLRLLNHL	KTIYDEFIN-	AVIKYNNHDQ	SLRSRLIGIS	SI--------	--DKTKFVSE	FNSTGCKQVN	QMLEKKISRL	KNEAQEEQDE
PCHAS_130120	DMLSKSVNQC	DPYIRLLNHL	KTIYNEYIK-	AVIKDN-DHD	ESLRNQLIEL	SSI-------	--NSKLVGSE	FNSEGCKKLH	KKLTQTTPNI	IKMGIKMLED
PCHAS_070170	YNKIYNIKQC	GPYLELLDNL	KEIYNSFIKT	AKSQNSHGDN	ILNQLVELPQ	I---------	--GNINDKYN	FKSKGCEKLH	KKLENNNSKL	IKMGIRMLKG
PCHAS_100060	YSQIYKINKC	DPYLELLDHL	KTPYDNFIKT	AKSKNLHGDD	ILNQLHELPP	I---------	--NKKKFESS	FESPGCKKVH	KRLENNKSKI	IKMGIKMLKD
PCHAS_146770	RTIHNSVNGC	KPYLHLLDSL	KKIYENFILT	KIINN-KNLE	KLSRRIKSLK	TF---KDEDK	YFISDNEELS	FNTEACGKVK	LEDEELGKQI	ASKGSPSKPK
PCHAS_146790	KTTHNAVKSC	KPYLHLLDSL	KRIYEDFRWE	--KII-NNND	SLSNRIESLK	TF---GNEDH	YFVSDSEELS	FNNEGCGKVK	LEDEELGKKI	ASKDSQGKQK
PCHAS_060160	KTIHNSVNGC	KPYLHLLDSL	KKVYENFRST	RIINN-KSLD	KLSKRVEPFK	TF---GNEDK	YFVLDSEVLS	FDNEECGKVK	TQDEELGKQI	ASKGSPSKQQ
PCHAS_000260	KTTHNAVKDC	KPYLHLLDSL	KKVYEDFLWD	-KIIN-NSDS	SLSNRIKSLK	TF---GNKDE	YFVSDSEVLS	FDNEGCGKVK	SQDEEIGKKI	TLTNSQNKAQ
PCHAS_130280	RTTHSAVNGC	KPYLHLLDSL	KKMYENFIST	KLIDN-KSFE	KLTRRIEPLK	TF---KNENK	YFVSDNEALS	FDTEACGKVK	SQDEELGKQI	ALTKSQSISK
PCHAS_083720	KTTHKAVKDC	KPYLHLLDSL	KKVYEDFLWN	KIITN-NDSN	ILSNRIKPLK	TF---GNKDE	YFVSDSEVLS	FDNEGCGKVK	SQDEEIGKKI	ASTNSQSISK
PCHAS_090010	RNIYESVKEC	KSYTHLLDSL	KTIYEYIKSY	-KISN-NISI	EKVVLFDATP	SLTTSDYQNK	YFIPNYETLS	FSDEGCGRVK	SKDEKDGKKG	SLPDLQNTLQ
PCHAS_114600	RNIYESVKGC	KSYTHLLDSL	KTIYEYIKSY	------KITG	NKVLLFVSTP	SLTTSDHKNE	YFIHNYETLN	FGDEGCEKLK	LKDEKDGEKG	SLPDLQSIQG
PCHAS_146860	DEISKFVKQC	GPYIELMHHI	KAIYEEFRQT	--IKE-NIED	PVFSQLMELP	SI--------	--DKTKSGSE	FKSNKCNKVH	EKIIKKLPKL	IKKEKDKLNG

Supplementary data 1 (CIR alignment)

PCHAS_130090	NELSKFVNQC	GPYRGLLDHL	KTIYNDFRQT	-AIRE-NAHN	KIFDQILEFP	SI--------	--DKTKYGSE	FESLECKQVH	DKLIKNLPRL	VKKENGELNY
PCHAS_030170	DKISKLIKRC	GPYIELLDHL	RTIYNEFIKR	-VKRE-HHHG	DILDRLMELP	EI--------	--DKTKLGCK	FKTGRCIRLH	QKLEKSNSKI	IKLGIKMLKD
PCHAS_130060	GQISNLIKQC	DPYLELLDHL	RTMYNEFIKS	-VKRE-NLHK	DIHAQLMELP	PI--------	--EKNMFGRN	FKTKRCIRLH	QKLENKNSKL	IKLGIKMLKD
PCHAS_030200	TALSKFVNPC	SPYLELLDHL	KTLYDDYRDA	-AIQN-KIHG	KTPDLLRRFP	EI--------	--DKTTQKFN	FTSPECKKVH	EQLIKNAQKL	IKEEKEKEEG
PCHAS_060080	TELSKFVNPC	SPYLELLDHL	KTLYDDYRED	-AIQN-KIHG	RTPELLRRFP	EI--------	--DNTTKKFN	LKSPECKKVH	EQLIKNAQKL	IKEEKEKEEG
PCHAS_060110	YFINSKAFKC	NPYIELLTNL	KKKYDEYKSL	--IIK-KFPK	NANNFFSDFP	PI-------N	NSNNNHPEQQ	FESKGCELLH	VIYQRPGRKY	KPKKRQKMLK
PCHAS_030140	HFLKQRVSRC	SPYAQLLVDL	KKTYDEYKIL	--VNK-KIEQ	GKFSDFPSIS	NY--------	--KYSPPELE	FESSGCKRVH	LFFQKHGTKY	KPKSIMGVFK
PCHAS_130100	YFLNQRISRC	SPYAQLLVDL	KKTYDEYKIL	--VNK-KIQQ	GDKIKFSELP	PL-------S	NNKNNQPELT	FDNIGCKLVH	LFLQKHGTKY	KPKKILKVPK
PCHAS_041990	YFINSKAFKC	NPYIELLTNL	KKKYDEYKSL	--IIK-KFPK	NANNFFSDFP	PI-------N	NSNNNHPEQQ	FESKGCELLH	VIYQRPGRKY	KPKKRQKMLK
PCHAS_130080	ELINYQVSNC	DPYIQLFVNL	NKTYDEYRKL	--AIN-KFSK	DLKNILTFLP	II--------	-NNDDQKDLQ	FQSQGCKDLH	AMFEQSYKPR	PKRRQRMPKP
PCHAS_030190	DLINTQVSNC	DPYVQLLINF	KKTYDEYKGL	--VIN-KIPK	NPINNNDDQP	EL--------	---------L	FESNGCKELH	EFFQQIFQKH	KPKKQLRRSK
PCHAS_060090	DLINTQVSNC	DPYVQLLINF	KKTYDEYKGL	--VIN-KIPK	NPINNNDDQP	EL--------	---------L	FESNGCKQLH	EFFQQIFQKH	KPKKQLRRSK
PCHAS_041970	ELINSDFSDC	DPYVQLFINL	KNIYDKYRNL	--AIK-QIPK	DDSVNSLTRP	EI--------	NNKDNEPQLK	FQSNGCKELH	LRFYKLRIKR	KPKKKPRILK
PCHAS_041980	DLINSQFSSC	DPYVQLLINL	KKLYDEYRNL	--AIK-QIPK	DDTVNSLTCP	EI--------	NNNDNQPNLQ	FQSNGCKELH	DFFRQISRKR	KPKRPSKGSK
PCHAS_042020	TSLYDEVKEC	YPYFKLLKNF	KKTYDDYRNS	-IMYT-IGNN	SMSFLNLPLT	ELKIPNWSDE	YMDESLEIED	FMTSGCAKLH	SKYLQLEKKN	TSQDTQKEQP
PCHAS_060070	TSIYNETKEC	YPYFKLLKNF	KKIYDDYRNN	IIYTI-VSNK	FLTYLNLPFT	ELKIPDWNDE	FMEESLEIDD	FMTSGCAKLH	SKYIEHQQQN	ISQDLPKEQF
PCHAS_011330	TKIYNELNKC	GPYIKLLSNL	KTTYDNFRTA	VINAD-KTKK	LNKVIQELKP	EIKINSSTNE	FDDECKKAHS	NAEKNSLKDQ	PKDPPQGPPK	VPPKVPPHVP
PCHAS_000220	TKIYNEINRC	GPYVNLLKNL	KTTYDNFRTS	VISTD-KTKN	LNKVIQELKP	EINIDAITKE	FDNECKKVHS	NAEKNNLKDP	SKDPSKDPSK	DPPQGTPKVP
PCHAS_042070	KNIYNNVKDC	YSYVHLLKYL	KNIYDVIRND	-AIKE-IAAK	KLDASTISLM	DLTTSDWNQI	FLDESDQIID	FHTQKCVELY	SKTVKKQEEH	ESKIPPKAEP
PCHAS_110030	INIYKDVKEC	YSYFHLLKFL	KNIYDVIRND	-AINE-TVAK	KLNALTISLK	DLTPKDWDQR	FLDESDQTID	LYTKNCNALH	SEISKQAKKD	ISKNPSTEQS
PCHAS_140140	MNINKDIKDC	YSYFHLLKYL	KSIYDGIRND	--AIK-DADI	KLNDLTISLK	DLTTPEWNQR	FSDESDQIID	FHTQKCVELY	SELAEQVKKD	TPQNAPTEQS
PCHAS_010040	TSIYKDIKDC	YSYFHLLKFL	KNIYDGIRND	--AIKKDTNT	KLNTPTISLI	DLTPSNWDKR	FSDDSDKIVD	FHTQACINLY	SEFVQKVKKD	TPKNILEGQP
PCHAS_073150	MNIYKNIKDC	YSYFHLLKYL	KNIYDDIRND	--AIK-EAAN	KLDASTISLI	DLTPQGWDQR	FSDVSDQTID	LYTQNCVALH	SKIPQHAKKG	TSKDIPEAHP
PCHAS_073130	MFLYQNVFKC	NSYLHLLDNL	KKIYDSFRAA	--VKN-IDPK	S----AAYLQ	TLTTKENSDL	YFAKNFKTFE	FNGSGCQLQY	DDNIFETLEK	AKLHGNQTND
PCHAS_040060	MLLYQNFSKC	DSYLHLLDNL	KKTYEDFRTT	--TKN-GDPN	L-ASSLQTLT	TI---ENTDS	YLVKGFSTFD	FSNSKCQSEY	DDSILEKSKK	PEARTKQKDN
PCHAS_000430	MLLYQNVSEC	DSYLHLLDNL	KKTYEKFRST	--IKN-GDPN	L-ASSLQTLT	TI---ENTDS	YFVKGFSTFD	FSNSKCQSEY	DDEILEKWRK	DQARTKQKDN
PCHAS_104200	MLLYQNVSEC	DSYLHLLDNL	KKTYEKFRST	--IKN-GDPN	L-ASSLQTLT	TI---ENTGS	YLVEGFKNFD	FSDRKCQSEY	DDSILEKLEK	TKAQRKQKDN
PCHAS_114720	MLLYQNVSEC	DSYLHLLDNL	KKTYEKFRTT	--INN-GDSK	L-ASSLQTLT	TI---ENTDS	YFVEGFKNFD	FSDQKCQSKY	DDSILEKWKE	TQDRSEQKNN
PCHAS_000770	KTLYRNISEC	KSYLRLLNKL	KGIYDDFRNY	-VIQE-NSSK	N--NLETNLK	KLTTPNGIEM	DAVRSFISYT	FSKKKCNSLD	KKNTNPPKAG	KSPLQPSNQQ
PCHAS_130220	KILYNNISEC	RSYLRLLNKL	KGIYDDFRVS	-AIEE-SDSK	N--NLETKLQ	KLTREDGVEM	NAVRSFISYK	FSKKKCYSQK	KKPNPGPAPT	ITIQKESPGS
PCHAS_070040	RILYMNIYEC	KSYLHLLNKL	KGIYDDFRVS	-AIKE-TSSN	N--NLETILQ	KLTLGNGTEL	EAVRSFKTYD	FSGSECKLPK	KKNPKPKKTG	SPPLQPTKLE
PCHAS_011490	RTLYINIYEC	KSYLHLLNKL	KGIYDDFRNS	-AINK-NNSN	N--NLATNLQ	KLTKPDGEEM	NAVRSFISYK	FNKKICNSLH	KKATTSKPTN	PPGLPPEESH
PCHAS_070060	RTLYINIYEC	KSYLHLLNKL	KGIYDDFRNS	-AINK-NNSN	N--NLATNLQ	KLTKPDGEEM	NAVRSFISYK	FNKKICNSLH	KKTTTQKPTN	PPGLSSSSKE
PCHAS_030070	ISIYNDIHKC	QSYLNLLNKL	KGIYDDFRNY	-AIKE-TDSK	I--NLETNLK	KLTKPDGGEM	NAVKGFKSYN	FNNSKCKSLH	NKITMSKPTD	PPGLPRS---
PCHAS_137110	ISIYNDIPKC	QSYLDLLNKL	KGIYDDFRNY	-AIQD-TDSN	N--NLETDLK	KLTKPDGEEM	DAVKGFNSYN	FSNSKCKVKK	KSASLKKEDS	PSLTPSPEQP
PCHAS_114700	RTLYMNVSEC	KPYLHLLSKL	KGLYDDFRSY	-AIKN-TDSN	N--NLVTNLE	KLTLENGEEI	GATKNFTSYN	FSNQPCKAKK	KKKTDKPSLQ	SSNQLKDRQQ
PCHAS_137030	RTLYMNVSEC	KPYLHLLSKL	KGLYDDFRSY	-AIKN-TDSN	N--NLVTNLE	KLTLENGEEI	GATKNFTSYN	FSNQPCKAKK	KKKTDKPSLQ	SSNQLKDRQQ
PCHAS_011480	RTLYNNIFEC	KSYLDLLNKL	KGLYDDFRSS	-AIKK-NGSK	N--NLETILQ	KLTLGNGTEM	KAVRGFKTYD	FSGSECKFPK	KKKKPEKSSL	QPSNQLKDSK
PCHAS_050040	MILYNLLSEC	KSHLHLLNKL	KYIYDQFREP	--TMK-EIRE	Q--NLSIPLQ	TLTTIAGVEM	RSSKVFKSYN	FSNTKCKRKV	KKKTDKPSLQ	SSSKKESLPP
PCHAS_000470	RTLHMNIYEC	KSYLNLLNKL	KGIYDDFSSA	--IKK-TGSN	N--ELATKLK	KFTLEDGTEM	AAVRGFKTYN	ISNTKCKSLH	KKMPKPKKAV	KSSLQSSSKK
PCHAS_000170	KTLYNNISKC	QSYLDLLNKL	KGIYDDFSSA	--IMR-NRSN	N--NLATKLK	KLTKPDGKEM	DAVRGFKTYN	FSNSKCKFPP	PKKKIVSSKK	AEPPGPQTSS
PCHAS_130170	KNLYLNIYKC	KPYLDLLNKL	KGIYDDFSSF	--IKK-NSSN	S--KLAAKLQ	TLTPKDGKEM	KAVRGFKTYD	ISNTKCKFPQ	KKITNPKKAD	KSPLQSSSKE
PCHAS_000660	KNLYLNISEC	KPYLDLLNKL	KGIYDDFSSV	--IKK-YNPN	S--ELATKLK	KLTPENGKEM	DAVRGYKTYD	FSTEQCKFPK	KKKTKPKKQD	KSPLQLSNQL
PCHAS_040080	KNLYLNISEC	KPYLDLLNKL	KGIYDDFSSS	--IKK-NDSN	N--ELATKLK	KLTPKDGKEM	DAVRGFKTYN	FSGSQCKFPK	KK-IKPKKPN	PPRLPPSSKE
PCHAS_070070	KTLYLNISKC	KPYLDLLNKL	KGIYDDFSSV	--IKK-NNPN	S--ELATKLK	KLTPKDGKEM	EAVRGFKTYN	FSGSQCKFPP	KKNTKPKKAD	KSSLQPSPKE
PCHAS_137090	KTLYMNVSEC	KPYLDLLNKL	KGIYDDFRNY	-GIKN-NSSN	N--ELETKLK	KLIPKDGEEM	AAVRGFKLYN	FSNQPCKGKK	KKKTDKSKKA	DSPGLPSSSK

Supplementary data 1 (CIR alignment)

```
                 ....|....| ....|....| ....|....| ....|....| ....|....| ....|....| ....|....| ....|....| ....|....| ....|....|
                       305        315        325        335        345        355        365        375        385        395
PCHAS_000950     ENGEEGEEKT KESNPVETPG PVAIENGLIG IGGVALLIPI ISTVLYKYWF ARWRNKSERK KSVKKVINL- ---------- ---------- ----------
PCHAS_001010     GNGEGNVSQK HTTEIQVNPP KTEL--ELIG IGGIALLIPV ISAVLYKYWF VGWRKKSTRK KNTKKVINL- ---------- ---------- ----------
PCHAS_140090     ---------- ----SDVTSS SS-IGRKLIP VFLAF-AISV FLGIAYKYSL FGFDKRLQRK QLREKIKK-- ---------- ---------- ----------
PCHAS_000730     ---------- ----FEVTSS NS-IATKLIP TLLIF-AMSF FFGIAYKYSL FGFGKRSQKQ YLKKRLKK-- ---------- ---------- ----------
PCHAS_070160     ---------- ----SGVTSP SS-IAKTLIP ALLIF-AIPV FVGIAYKYSL FGIDKRLQRI HSRGKLRKI- ---------- ---------- ----------
PCHAS_030020     ---------- ----SEFASS SS-ITIKLIP VLLTF-TIPF FLRIAYKYSL FGFGKRSQKQ YLGKRLKK-- ---------- ---------- ----------
PCHAS_000360     ---------- ----SEAILS GS-VASKLIP ALLVF-AMPV FLGIAYKYSL FGFGKRSQKR YLRENIKK-- ---------- ---------- ----------
PCHAS_110020     ---------- ------KTPP SS-IASKLIP VLLTC-SISF FLGIAYKYSL FGFDKRRQRQ YLRENLKK-- ---------- ---------- ----------
PCHAS_050060     ---------- -------KTT PT----KLIT VLLIFAAIPI SLGIAYKYSL FGFDKRLQRH DIRNRIKKI- ---------- ---------- ----------
PCHAS_010030     ---------- ----SEVETS SS-IASKLIP GLLVF-TIPI FLGIAYKYSL FGFGKRSQKQ YLRKMLKK-- ---------- ---------- ----------
PCHAS_030030     ---------- ------KAPP SS-IPRKLIP VLLTF-SIPF FLGIAYKNSL FGFHKRVQRK HLRERLKK-- ---------- ---------- ----------
PCHAS_000350     ---------- ------KAPP SS-IAKTLIP VLLTF-AIPV FLGIAYKYSL FGFDKRVQRQ NLREKPKKI- ---------- ---------- ----------
PCHAS_011530     ---------- -----EHASS SS-IASKLIP GLLIF-AIPI LLGVAYKYSL FGFDKRLKRI HSREKIKKI- ---------- ---------- ----------
PCHAS_073160     ---------- -----EHASS SS-IASTLIP VLLAF-TIPF FLGVAYKYSL FGFDKRLQRQ YIREKVKKI- ---------- ---------- ----------
PCHAS_070090     ---------- ------KAPQ SS-IESKLIP ALLIF-AIHF SWELLI-YSL FGFDKQRHRQ YLREKLKKI- ---------- ---------- ----------
PCHAS_000580     ---------- ----SGVTSP SS-IASTLIP GLSTF-AIPA FLGIAYKYSL FGIDKLFQRQ YIRKKLKKI- ---------- ---------- ----------
PCHAS_140130     ---------- ----SQSSST KS----AIIS VTFIFVAIPI FLGIAYKYSL FGFDKRLHKQ YLREKVKKI- ---------- ---------- ----------
PCHAS_140040     ---------- ----SRCSST KN----ALIS ITFIFVAISI FLGFAYKHSL FGFGKRSQKQ YLREKRKKI- ---------- ---------- ----------
PCHAS_000740     ---------- ----SRCSST KN----ALIS ITFIFVAISI FLGFAYKHSL FGFGKRSQKQ YLREKRKKI- ---------- ---------- ----------
PCHAS_100030     ---------- ----SRCSLT KN-L------ ITFIFVAIPI FLGFAYKYSL FGFGKRSQKQ YLREKRKKI- ---------- ---------- ----------
PCHAS_001120     ---------- --------SR CS-I--KNAL ISITFIAIPI FLGVAYKYSL FGFGKRSQKQ YLREKRKKI- ---------- ---------- ----------
PCHAS_011520     ---------- --------PR SF-IKVTLIP ITFIFVVIPI FLEFAYKYSL FGFGKRSQKQ YLREKRKKA- ---------- ---------- ----------
PCHAS_070030     ---------- --------PR SF-IKVTLIP ITFIFVAIPI FLEFAYKYSL FGFGKRSQKQ YLREKRKKA- ---------- ---------- ----------
PCHAS_073190     ---------- ----SRCSLT KN-L------ ITFIFVAISI FLGFAYKHSL FGFGKRSQKQ YLREKRKKI- ---------- ---------- ----------
PCHAS_000570     ---------- --------PR SF-IKMTLIP ITFIFVAIPI FLEFAYKYSL FGFGKRSQKQ HLREKRKKI- ---------- ---------- ----------
PCHAS_114730     ---------- --------SR CP-IKNALIS ITFIFVAIPI FLEFAYKYSL FGFGKRSQKQ YLREKRKKA- ---------- ---------- ----------
PCHAS_010020     ---------- ----SEDAPS SS-ITSKLIP VLLAF-TIPV FLGIAYKYSL FGFDKRLQKH DLRKRLKK-- ---------- ---------- ----------
PCHAS_000280     ---------- ------KTTS SL-IASKLIP VLLMF-AIPI FLGIAYKYSL FGFDKRVQRQ TLRKKYK--- ---------- ---------- ----------
PCHAS_000490     ---------- ------KTTS SL-IASKLIP VLLMF-AIPI FLGIAYKYSL FGFDKRVQRQ TLRKKYK--- ---------- ---------- ----------
PCHAS_000030     ---------- ----SEDTSS SS-AASKLIP VISIFVAIPI LLGVAYKYSL FGFDKGLHRQ YLREKVKKI- ---------- ---------- ----------
PCHAS_000090     ---------- ----SEDTSS SS-AASKLIP VISIFVAIPI LLGVAYKYSL FGFDKGLHRQ YLREKVKKI- ---------- ---------- ----------
PCHAS_000270     ---------- ----SEDTSS SS-AASKLIP VISIFVAIPI LLGVAYKYSL FGFDKRLHRQ YLREKVKKI- ---------- ---------- ----------
PCHAS_000120     ---------- ----SEAISS GS-VSTKLIS VLLIFSAIPL FLGIAYKYSL FGIDKLFQRQ YLREKVKKI- ---------- ---------- ----------
PCHAS_001090     ---------- ----SEAISP SS-TASKLIA VLSIFVAMPI FLGIAYKYSL FGFGKRSQKQ YLRDKLKKI- ---------- ---------- ----------
PCHAS_011510     ---------- ----SEVTSS SS-ILNTVIP VLSTF-AIPV FLVVSYKYSL FGFDKLFQRQ YIRNKLKKV- ---------- ---------- ----------
PCHAS_070050     ---------- ----SEATTS SS-ILNTVIP VLSTFSVISL FLGVSYKYSL FGFGKRSQKR YLRENIKK-- ---------- ---------- ----------
PCHAS_000720     ---------- ----TSKGTS SP-ILNTVIP VLSTFSVISL FLGVAYKYSL FGFGKRSQKR YLRENIKK-- ---------- ---------- ----------
PCHAS_070020     ---------- ----SEGASS SS-MLNTVIP VLSTFSVISL FLGVAYKYSL FGFGKRSQKQ YLREKRKKI- ---------- ---------- ----------
PCHAS_104260     ---------- ----SEGASS SS-MLNTVIP VLSTFYVISL FLGVAYKYSL FGFGKRSQKQ YLREKRKKT- ---------- ---------- ----------
PCHAS_140020     ---------- ----SEGASS SS-MLNTVIP VLSTFSVISF FLGVAYKYSL FGFGKRSQKQ YLREKRKKI- ---------- ---------- ----------
PCHAS_030060     ---------- ----PDATPS SS-TLNTVIP VLSTFSVISL FLVVSYKYSL FGIDKLFQRQ YRRNKLKKT- ---------- ---------- ----------
PCHAS_000750     ---------- ----LEATPS SS-TLNTVIP ILSTF-AIPV FLGVAYKYSL FGFDKLFQRQ YIRNKLKKI- ---------- ---------- ----------
PCHAS_000680     ---------- ----PEATPS SS-ILNTVIP VLSTFSVISL FLGVSYKYSL FGFGKRSQKR YLRENIKK-- ---------- ---------- ----------
```

Supplementary data 1 (CIR alignment)

```
PCHAS_000420    ---------- --------PR SF-IKVTLIP ITFIFVAIPI FLELAYKYSL FGFGKRSQKQ YLREKRKKA- ---------- ---------- ----------
PCHAS_000100    ---------- --------PR SF-IKVTLIP ITFIFVAIPI FLEFAYKYSL FGFGKRSQKQ YLREKRKKA- ---------- ---------- ----------
PCHAS_000310    ---------- --------PR SF-IKVTLIP ITFIFVAIPI FLELAYKYSL FGFGKRSQKQ YLREKRKKA- ---------- ---------- ----------
PCHAS_040040    ---------- --------PR SF-IKVTLIP ITFIFVAIPI FLGFAYKHSL FGFGKRSQKQ YLREKRKKI- ---------- ---------- ----------
PCHAS_120060    ---------- --------PR SF-IKVTLIP ITFIFVAIPI FLGFAYKHSL FGFGKRSQKQ YLREKRKKI- ---------- ---------- ----------
PCHAS_104230    ---------- --------PR NF-IKVTLIP ITFIFVAIPI FLEFAYKYSL FGFGKRSQKQ YLREKRKKA- ---------- ---------- ----------
PCHAS_030040    ---------- --------PR GF-IKVTLIP ITFIFVAIPI FLEFAYKYSL FGFGKRSQKQ YLREKRKKA- ---------- ---------- ----------
PCHAS_000340    ---------- --------PR NF-IKVTLIP ITFIFVAIPI FLEFAYKYSL FGFGKRSQKQ YLREKRKKA- ---------- ---------- ----------
PCHAS_001110    ---------- -----EDASS GS-VASKLIP VLSIF-AISL FLGIAYKYSL FGFDKLFQRQ YIRKKLKKI- ---------- ---------- ----------
PCHAS_120040    ---------- ----SEFASS SS-VASKLIP VLLTF-AIPI FLGIAYKYSL FGIDKLFQRQ YIRKKLKKI- ---------- ---------- ----------
PCHAS_000130    ---------- ----SEVTSS SS-VASKLIP VLSIF-AISL FLGIAYKYSL FGFGKRVHRK HLREKLKK-- ---------- ---------- ----------
PCHAS_000400    ---------- ----SEVTSS SS-VASKLIP VLSIF-AISL FVGIAYKYSL FGIDKLFQRQ YRRNKLKKT- ---------- ---------- ----------
PCHAS_040020    ---------- ----SEVTSS SS-VASKLIP VLSIF-AISL FVGIAYKYSL FGIDKLFQRQ YIRKKLKKI- ---------- ---------- ----------
PCHAS_000110    ---------- ----FEATSP IS-IASILIP VLLTF-SITF FLGIAYKYSL FGFDKRLNRQ YLREKIKKI- ---------- ---------- ----------
PCHAS_120050    ---------- ----SEDTSS SS-IASKLIP TLLIF-AIPV FLGIAYKYSL FGFDKRFRRK YSREKLKNK- ---------- ---------- ----------
PCHAS_104250    ---------- ----SEATSS SS-VASKLIP VLSIF-AISL FLGIAYKYSL FGFDKQLQRQ YLREKLKKI- ---------- ---------- ----------
PCHAS_000320    ---------- ----SEATSS SS-VASKLIP VLSIF-AISL FLGIAYKYSL FGFDKQRHRQ YLREKIKKI- ---------- ---------- ----------
PCHAS_000410    ---------- ----SEATSS SS-VASKLIP VLSIF-AISL FLGIAYKYSL FGFDKQRHRQ YLREKLKKI- ---------- ---------- ----------
PCHAS_140030    ---------- ----FEATPS SS-IASKLIP ALLVF-AIPV FLGVSYKYSL FGFDKRFQRQ YLREKLKKI- ---------- ---------- ----------
PCHAS_114740    ---------- ----FEATSP IS-IASILIP VLLTF-SITF FLGIAYKYSL FGFDKRLQRQ YLREKLKKI- ---------- ---------- ----------
PCHAS_040030    ---------- ----SEDASS SS-IASKLIP ALLIC-SIPI LLGIAYKYSL FGFDKRLHRQ YLRDKVKKI- ---------- ---------- ----------
PCHAS_070130    ---------- ------KTPP FS-ITSKLIS VLLIFVAIPI SLGIAYKYSL FGFDKRLHRQ YLREKLKKI- ---------- ---------- ----------
PCHAS_073180    ---------- ------KTLQ FS-ITSKLIP VLLAF-AIPI FLGIAYKYSL LGFDKRLHIQ YLREKRKKI- ---------- ---------- ----------
PCHAS_000300    ---------- ------KTPL IL-IARKLIP VLLAF-AIPI FLGIAYKYSL FGFDKRLHIQ YLREKRKKI- ---------- ---------- ----------
PCHAS_120070    ---------- ------KTLQ FS-ITSKLIP VLLAF-AIPI FLGIAYKYSL LGFDKRLHIQ YLREKRKKI- ---------- ---------- ----------
PCHAS_001130    ---------- ------KTLQ FS-ITSKLIP VLLAF-AIPI FLGIAYKYSL LGFDKRLHIQ YLREKRKKI- ---------- ---------- ----------
PCHAS_100040    ---------- ------KTLQ FS-ITSKLIP VLLAF-AIPI FLGIAYKYSL LGFDKRLHIQ YLREKRKKI- ---------- ---------- ----------
PCHAS_000560    ---------- ------KTLQ FS-ITSKLIP VLLAF-AIPI FLGIAYKYSL LGFDKRLHIQ YLREKRKKI- ---------- ---------- ----------
PCHAS_050020    ---------- ----SEVTSQ NS-IASKLIP VLSIFVAIPI FLGISYKYSL FGFDKRRHRQ YLREKVKKI- ---------- ---------- ----------
PCHAS_000020    ---------- ----SEYKPS SS-VENALIP VLSIFVAIPV FLGIAYKYSL FGFDKQRHRQ YLREKIKKI- ---------- ---------- ----------
PCHAS_000290    ---------- ----SEDISS KS-MANKLIP GLLIFAAIPV FLGIAYKYSL FGFDKQRHRQ YLREKIKKI- ---------- ---------- ----------
PCHAS_060020    ---------- ----SEDISL KS-IANTLIP VLSIF-AVPI FLGIAYKYSL FGFDKRLHRK YLREKVKQI- ---------- ---------- ----------
PCHAS_001040    ---------- ----SEATSS SS-IASKLVP VLSIFVAIPI FLGFAYKHSL FGFDKRLNRQ YLREKVKKI- ---------- ---------- ----------
PCHAS_114750    ---------- ----SEATSS SS-IASKLVP VLSIFVAIPI FLGFAYKHSL FGFDKRLNRQ YLREKVKKI- ---------- ---------- ----------
PCHAS_000140    ---------- ----SEATSS SS-IASKLVP VLSIFVAIPI FLGFAYKHSL FGFDKRLNRQ YLREKVKKI- ---------- ---------- ----------
PCHAS_000070    ---------- ----SEDASS SL-IASKLIP VLLVFGAIPI FLGVAYKYSL FGLDKRLQRQ YLRDKVKKI- ---------- ---------- ----------
PCHAS_030080    ---------- ----SDVTTS NS-IASKLIP ILLIF-SIPI FLGIAYKYSL FGFDKRVQRQ NFREKLKKI- ---------- ---------- ----------
PCHAS_073200    ---------- ----SEDTSS SS-IASTLIP ILSIF-SIPI FLGIAYKYSL FGFDKRLQRQ YLREKLKKI- ---------- ---------- ----------
PCHAS_120020    ---------- ----SEVTTS SS-VASKLIP ALLVF-AIPI FLGISYKYSL FGFDKRRHRQ YLREKVKKI- ---------- ---------- ----------
PCHAS_000060    ---------- ----SEYKPS SS-VASKLIP ALLIC-SIPI FLGISYKYSL FGFDKRLHRQ YLREKLKKI- ---------- ---------- ----------
PCHAS_130020    ---------- ----PDAASS SS-IASKLIP ALLIF-AIPV FLGISYKYSL FGFDKRAHRQ YLREKIKKI- ---------- ---------- ----------
PCHAS_000150    ---------- ----PESAPS SP-IASKLIP ALLVF-AIPI FLGIAYKYSL FGFDKRLHRQ YLREKLKKI- ---------- ---------- ----------
PCHAS_000500    VSL------- ----SDEPPT EV-LGNKVTY IVVPFTLILI ILGILYKYLI HGQRKKLKRK KNANKIINL- ---------- ---------- ----------
PCHAS_001100    TSL-PDLSTT ELRITQEEPQ SN--GSKLPY IAVPFILIPI IFAISYKYLT LMWKKKMKSK KNARKIINL- ---------- ---------- ----------
PCHAS_042010    EPITKSPKSE SAQSLSEKSG SAGAPVGIIG IGIIGISIFI ILAIMYKYLS FGSRKNSKKK KITKKVINLV DGKKREKTFI NSIDREKKEK IIVNS-----
PCHAS_000040    TILSYQTSTE LSDSREESLP KIEVGNKLPY IAVPFILIPV IFGISYKYLT LMWKKKMKSK KNVRKIINL- ---------- ---------- ----------
PCHAS_000390    ISLPVRSTTE LTDNRENTLS VNEVGNKLPY IAVPFILIPV IFGISYKYLT LMWKKKMKSK KNVRKIINL- ---------- ---------- ----------
```

Supplementary data 1 (CIR alignment)

```
PCHAS_030180    NSLPLLDFLN KLKGKEGESP SSGLSNKVIY IVVAFISILI ILGISYKYLP LGGVKKLKRK KKMKKIINM- ---------- ---------- ----------
PCHAS_130070    NSLQLIDFLN KLKGKEGESP VISVSNKIIY IVVAFISILI ILGISYKYLS LVGVKKLKRK KKMKKIINM- ---------- ---------- ----------
PCHAS_011500    NSLSSLSRKD GEKNEYDSTT DKSVSGIIIY IVAGFISILI IFGISYK--- --INNKIIIK YKIEKIAK-- ---------- ---------- ----------
PCHAS_146850    DS-------- ----LSDQPS TEVIANKLPY IAVPFILVSI ILGISYKYLT HGWRKRSNAK KKMKTIINL- ---------- ---------- ----------
PCHAS_070100    GSLSSQSSTK LSDSLGNTLP VKEVTNKLPY IAIPFFLIPV ILGISYKYLT HGQRKKSNGK KKVKTIINL- ---------- ---------- ----------
PCHAS_060050    ---------- ----KQITTH NIGLGNKLLY IAIPFILIPI ILGISYKYLT PVWRKMMKRK TMKKIINSS- ---------- ---------- ----------
PCHAS_030110    ---------- ----KQVTTH KIGLGNKLPY IAIPFILIPI ILGISYKYIT YRQRKKLNSK KKYANDYKF- ---------- ---------- ----------
PCHAS_130050    ---------- ----EQVTTH KISLGNKLPY IAIPFILIPI ILGISYKYLT PLWRKMMKRK -TMKKIINL- ---------- ---------- ----------
PCHAS_030090    ---------- ----PGISLP GLPVGNKLPY IAVPLILIPI ILGISYKYLT PVWRKKSKRK -AMKKIINL- ---------- ---------- ----------
PCHAS_146870    ---------- ----KVLTLP NSSLGNKLPY IAVPLILIPI ILGISYKYLT PVWRKKTKRK -AMKKIINL- ---------- ---------- ----------
PCHAS_130030    ---------- ----KVPTLP NSSLGNKLPY IAVPLILIPI ILGISYKYLT PVWRKKMKRK -TMKKIINL- ---------- ---------- ----------
PCHAS_040110    ---------- ----EIDTTP GISLGNKLPY IAVPLILIPI ILGISYKYLT PVWRKKTKRK -AMKKIINL- ---------- ---------- ----------
PCHAS_042030    TS-------- ----TNASLP GPSVGNKLPY IAVPLILIPI ILGISYKYLT PVWRKKMKKK -NMKKIINL- ---------- ---------- ----------
PCHAS_060140    IL-------- ----TKANLP GQPIGNKLPY IAVPLILIPI ILGISYKYLT PVWRKKTKRK -AMKKIINL- ---------- ---------- ----------
PCHAS_030210    TF-------- ----TNASLP GPSVGNKIPY IAIPFILIPI ILGISYKYLT PVWRKKSKRK -AMKKIINL- ---------- ---------- ----------
PCHAS_060060    TP-------- ----TEDSLP GSSVGNKIPY IAVPLILIPI ILGISYKYLT PVWRKKTKRK -AMKKIINL- ---------- ---------- ----------
PCHAS_050070    ---------- ----TKVSLP VPSVANKLSY IAVPLILIPI ILGFSYKYLT PVWRKKAKRK -AMKKIINL- ---------- ---------- ----------
PCHAS_040050    ---------- ----TEVSLS VPSVANKLPY IAVPLILIPI ILGFSYKYLT HSRRKKSNAK KKMKAIINL- ---------- ---------- ----------
PCHAS_001050    ---------- ----TEVSLS VPSVANKLPY IAVPLILIPI ILGFSYKYLT PVWRKKLNAK KKVKAIINL- ---------- ---------- ----------
PCHAS_083760    ---------- ----TVVSLP DSSVGNKLPY IAVPLVLIPI ILGISYKYLT PVWRKKAKRK -AMKKIINL- ---------- ---------- ----------
PCHAS_140070    ---------- ----TVVSLA DPSVGNKLPY IAVPLILIPI ILGISYKYLT PVWRKKMKRK -TMKKIINL- ---------- ---------- ----------
PCHAS_000180    TP-------- ----AVVSLP SPPVGNKLPY IAVPLILIPI ILGISYKYLT PVWRKKTKRK -AMKKIINL- ---------- ---------- ----------
PCHAS_001060    ---------- ----AVVSLP DSSVGNKLPY IAVPLILIPI ILGISYKYLT PVWRKKMKRK -TMKKIINL- ---------- ---------- ----------
PCHAS_011450    ---------- ----LPVIPT SINNGNKLPY IAVPLVLIPI ILGISYKYLT PVWRKKMKKK -TMKKIINL- ---------- ---------- ----------
PCHAS_060030    ---------- ----RPNSLP GLPVGNKLPY IAVPLVLIPI ILGISYKYLT HGRRKKLNAK KKVKAIINL- ---------- ---------- ----------
PCHAS_120030    TP-------- ----AVVSLP SPPVGNKLPY IAIPFILIPI ILGISYKYLT PVWRKKAKRK -AMKKIINL- ---------- ---------- ----------
PCHAS_030120    DSL-PVRSQI KLADSREEPQ SS--GNKLPY IAVPFILIPI ILVISYKFLA PAWRKRMKKK -NMKKIINL- ---------- ---------- ----------
PCHAS_114640    NSLSGSQEGS VFGIEGDMHE SNSIGNKLPY IAVPFVLIPI ILGISYKYLI HGRGKKVKKK KNAKNIINL- ---------- ---------- ----------
PCHAS_130350    SSSEPPSEAP PSEAPPTEPA PSSLSISKSV FSVIVIATPI ILAIMYKYLY YGRRKKPKRK KNMKKVINS- ---------- ---------- ----------
PCHAS_030150    EN-------- ----SDQITP TNELINNKIK IGVIALSIPI ILVFIYKYFP WKRTKKPKKT KKMKRVVNL- ---------- ---------- ----------
PCHAS_060120    EETTILLDGF AVPYYEITTP DYEIHQNRIK IGVIALSIPI VLVFIYKYFP WKRTKKPKKT KKMKKVINLL DRKKTKKIDI HSIDDKKTIQ -TIINS----
PCHAS_041960    NIPIEEEVCI ENWAPESPNF ETELHQNRIK IGVIALSIPI ILVFIYKYFP WKRTKKPKKT KKMKKVVNLL DRKKTKTIGI NSIDDKKTMQ ITINSDDK--
PCHAS_130110    DFVE------ ----SDQVSP ECELDQNRIK IGVIALSIPV VLVFIYKYFP WKRTKKPKKT KKMKRVVNLL DRKKTKKIDI NSIDDKKTIQ -T--------
PCHAS_041950    FSTLPEDLSK QLKSLEEHPP SSSIGQIPNY VIAIVISIPI ILLIMYKYLS YWRTKKSKGK TKMKKVINL- ---------- ---------- ----------
PCHAS_060130    LNALEKEGDT MKSIDNTTQN H-LTK-KLIA FSVIGIAIPI TLAIMYKYLS PWRTKKSKRK TKMKKIINL- ---------- ---------- ----------
PCHAS_130120    DKKRDNTSQN HERDPINISD QSSISDKLIA FSVIGIVIPI TLAIMYKYLS PWRTKKSKRK TKMKKIINL- ---------- ---------- ----------
PCHAS_070170    NAKRVKKPGA PEPAAPPPAA APTNQDDIVG IGIIFISTSM VLAFMYKYLP FKSAKNSRKE KNMKRVINL- ---------- ---------- ----------
PCHAS_100060    DAKKQIQHGQ QGTSPTPPPG P-ISN-DILG IGIIFISISI VLAIMYKYLS FGSAKNSKKE KNMKRVINL- ---------- ---------- ----------
PCHAS_146770    GTITTNLPPA KPAPANPNPP RPPMSTDIVG IGIIGTSLFV FLAIMYKYIS FGSAKNSKKE KNMKRVINLV GGKKRERTFI NSGDDKKMMK IIINS-----
PCHAS_146790    DPTKSPQKPA PAKPAPAKPA TTPTQKDIVG IGIIGISISI FLAIMYKYLS FGSAKNSKKE KSMKRVINS- ---------- ---------- ----------
PCHAS_060160    GSGNDQSGKV PTPGKAGVPS PPPTKDDILG IGIIGAAIFI FLAFTYKYLS FGSAKNLKKE KSMKSVINLV SGKKREKRFI NSGDGKKTEI IINS------
PCHAS_000260    DPGNKPAPAK PAPTSPGMPP KQPIQNDILG IGIIGAAIFI FLAFMYKYLS FGSAKNSKKE KSMKSVINLV SGKKREKRFI NSGDGKKTEI IINS------
PCHAS_130280    GTGDAKPAPA HTAAGKPTPP PAPILQDILG IGIIGAAIFI FLAFTYKYLS FGSAKNSKKE KNMKRVINLV SGKKREKRFI NSGDGKKTEI IINS------
PCHAS_083720    GTGDAHTAAG KPTPPPAQPV PTPIQNDILG IGIIGAAIFI FLAFTYKYLS FGSAKNSKKE KNMKRVINLV SGKKREKRFI NSGNGKKTEI IINS------
PCHAS_090010    GPGKSQQGQQ QSASSGASTP SPPMSTDLVG ISVIGVSISI FLAIMYKYLS FGSAKNSKKA KNMKRVINS- ---------- ---------- ----------
PCHAS_114600    DKLPAKPAPA KPVPPPQPSP PSPMASDLVG ISVIGVSISI FLAIMYKYLS FGSAKNLKKE KNMKRVINS- ---------- ---------- ----------
PCHAS_146860    YEDSVELQEE NREQKVESKD SHSITDDIVG IGIIGISIFI VLAFMYKYLS FGSRNNSKKK KITKKVINLV DGKKMEQTFI KSVDREKKPK IIVNSDDNKK
```

Supplementary data 1 (CIR alignment)

PCHAS_130090	YEESKKDATP	SKAQQPKQQP	STSIGTNIIG	IGIIGISIFV	FLAFLYKYLP	FGSRKKSKKK	KITKKVINLV	DGRKMEKTFI	KSIDRGKKSN	IIINSGDNKK
PCHAS_030170	DAKRKFLSQL	ENSQQGTSPV	PAIKEKNIIG	IGIIGISIFA	FIAFMFKYLS	FGSRKKSKKK	KITKKVINLV	DGRKMEKTFI	KSIDREKKSN	IIINSGDNKK
PCHAS_130060	DAKRPAPQQT	QTHAATKSVP	TPGLIENIIG	IGIIGISIFV	FLAFMFKYLS	FGSRKKSKKK	KITKKVINLV	DGRKMEKTFI	KSVDREKKSN	IIINSGDNKK
PCHAS_030200	NGKAHNTNAQ	PGKKSTPATP	GKSTLNNIIG	IGIIGISIFV	FLAFMYKYLP	FGSRKKSKKK	KITKKVINLV	DGRKMEKTFI	KSIDREKKSN	IIINSGDNKK
PCHAS_060080	KGKTAQPGKK	STPATPGKPP	PGPISDNIIG	IGIIGISIFV	FLAFMFKYLP	FGSRKKSKKK	KITKKVINLV	DGRKMEKTFI	KSIDREKKSN	IIINSGDNKK
PCHAS_060110	PPPDPQKDTK	QSAKEKDQPP	VKDIGENIIG	IGIIGISIFV	FLAFMFKYLS	FGSRKKSKKK	KITKKVINLV	DGRKMEKTFI	NSIDREKKSK	IIINSGDNKK
PCHAS_030140	SSSNAKEETI	QSIKKESQPS	EPSVHGDIIG	IGIIGISIFV	FLAFMFKYLS	FGSRKKSKKK	KITKKVINLV	DGRKMEKTFI	KSIDREKKSN	IIINSGDNKK
PCHAS_130100	SSSNTKEETK	QSIKKENQPS	EPSVAPDIIG	IGIIGISIFV	FLAFMFKYLS	FGSRKKSKKK	KITKKVINLV	DGRKMEKTFI	NSIDREKKSK	IIINSGDNKK
PCHAS_041990	PPPDPQKDAK	QSTIKEKQSP	EPSLINKVAI	MALMAVSIPI	VLIIMYKYLY	YGCGKTSKKK	KMVKKIINSQ	DGKRRKKKVI	SPIDGKSNLK	TVINSIGGEN
PCHAS_130080	KSNDNSTEKH	NEPQKETQPP	ESSLIIKVAI	MALMAVSIPI	ALVIMYKYLY	YGCGKTSKKK	KMVKKIINSH	NGKRTVKKII	NPIDGKRNLK	TVINPIDGKN
PCHAS_030190	YSSNSKKDAQ	QSTKKENQPP	ESSLVDKVSI	MALMVVSIPI	VLAIMYKYLY	YGWGKTSKKK	KMVKKIINSH	NGKRKVKKII	NPIDGKRTLK	TVINPIDGKN
PCHAS_060090	YSSNSKKDAQ	QSTKKENQPL	ESSLKVKVSI	MALMAVSIPI	ALVIMYKYLY	YGWGKTSKKK	KMVKKIINSH	NGKRKIKKII	NPIDGKRTLK	TVINPIDGKN
PCHAS_041970	SPSNQTLSKS	PENIQEGSPN	NNPVPKKAVV	ITLIVVSIPI	VLAIMYKYLY	YRCGKKPKKK	KKVKKIINS-	----------	--IDGKNTEK	TVINLIGGKN
PCHAS_041980	SSSNSQKNTD	QSTIKENPPS	EPSLKIKVAI	MGLMAVSIPI	VLIIMYKYLY	YGCGKTSKKK	KIVKKIINSN	NEKRRTKKVI	SPIDGKRTLK	TVINPNDRKN
PCHAS_042020	PSGSQKPASK	PQQSPKQEPP	PKGLPTNIIG	IGIIGISIFV	FLVFMYKYLS	FGSRKNSKKK	KITKKVINLV	DGKKMEKTFI	NSIDREKKEK	MIVNSGDNKK
PCHAS_060070	PSGSERPESE	QQSPKQEPPP	KTGIITNIIG	IGIIGISIFV	FLAFMFKYLP	FGSRKKSKKK	KITKKVINLV	DGRKMEKTFI	KSIDRGKKSK	IIANSGDNKK
PCHAS_011330	PQVPDTSPGK	PPGKPPGKPP	GKQLQNDVVG	IGIIAISTLI	MISIMYKYLS	FGWGKKTTKK	RNMRKVINLV	ESKKTEKTVT	NSVTGKKTLQ	IIINSSSQKK
PCHAS_000220	PQGPGQPKGP	QKGQPKGTPK	GTQLPSDVVG	IGIIVISTII	MISIMYKYLS	FGWGKKTTKK	RNMRKVINLV	ESKKTEKTVT	NSVTGKKTLQ	IIISSSSQKK
PCHAS_042070	QAGSQNKDGD	NSHTQKQQQA	STSLPTASTY	VKVIAILIPI	ILGIMYKCLS	SGWRKELKRK	KSMKKVINS-	----------	--IGGKKTTQ	IIINSSTKKK
PCHAS_110030	QSGSTALPPP	PEPQKQDSPT	PPPPKTNRPE	TVITVILIPI	ITLIIYKYLS	RERTKKSEKK	-NMKKVINL-	----------	--AYGKRKTQ	IIIQSCDRTK
PCHAS_140140	QSGGQEETPT	ALPQKQDSPS	PSQPEKNRPE	IVITVILIPI	ISLIIYKYLS	RERTKKSEKK	-NMKKVINL-	----------	--AYGKRKTQ	IIIKSCDRTK
PCHAS_010040	QLGSETLPQP	PIPAPQIQPQ	PPGMQKSLTG	VSVTVVLIPI	FLLVAYKFLS	REWTKKSEKK	-SMKRVINL-	----------	--ADGNRKTQ	IIIKSYDRNK
PCHAS_073150	QLGSDPPSQQ	AETPPQQQPS	PQGTEKSLTG	ISVTVVLIPI	FLLAAYKCLS	REWTKKSEKK	-NMKRIINL-	----------	--SDGNRKTK	IIIKSYDRNK
PCHAS_073130	KTKGELVPGS	QGATSDTTSS	KSGMDKKQVE	ISIIVLLIPI	ALAIMHKYLK	FGWRKELKKK	KNMKHIINLF	DVNKAPKTVI	NPINGKRPMQ	IIINSPTQKK
PCHAS_040060	GADTGSQSKE	NAKGSEHTDP	GSDIGNKQVG	ILIIVILIPI	TLAIMYKYLA	SGRKKELKRK	KNMKKVINL-	----------	--MEGKRQMQ	IIIKSSSQKK
PCHAS_000430	GAN-------	----EDNNPQ	NPKVGSKQTR	ILIIVILIPI	TLAIMYKYLS	FGRRNELKRK	KNMKKVINL-	----------	--MEGKRHMQ	IIIKSSSQKK
PCHAS_104200	GGNE------	----KQNTQP	ESSVGSKQTR	ILIIVILIPI	TLAIMYKYLS	FGRRNELKRK	KNMKKVINL-	----------	--MEGKRQMQ	IIIKSSSQKK
PCHAS_114720	EGNG------	----KQSSQP	ESSVGSKQTR	ILIIVILIPI	TLAIMYKYLS	FGRRNELKRK	KNMKKVINL-	----------	--MEGKRQMQ	IIIKSSSQKK
PCHAS_000770	KGGQEIKETK	QQSSTESSPQ	SAEVPTNRPE	IAITILLIPI	ISLIIYKYFL	SGWRKELKKK	QKMKKVINLF	GANKTAKTVI	NSSDGKKQVQ	IIIKSSSQKK
PCHAS_130220	HGV-SNAAGG	QLSNQENTSK	GSGTAQSLTG	ISVTLVLIPI	ILLIVYKYLS	SGWRKEMKRK	KNMKKVINLF	GANKTTKTVI	NSTYGKNQVQ	IIIKSSSRKK
PCHAS_070040	STSSLPLTPS	PEMQKQDSLS	PSQLHNKKIG	IPIIVIIISI	TLAIMYKFLV	FERRKKLKRK	K-MKKVPNLF	GVNKTT----	----------	----------
PCHAS_011490	KS--EKFSQS	EPNDSDIGPE	GSGTGTKKIG	IPIIVIIISI	TLAIMYKSKK	THIKTYKFRL	WGKIPLLNIY	KLMQEALHHL	LINFFCDFFC	DFFCDFFCDF
PCHAS_070060	VPPPPEESHK	SEKISQSEPN	DSGTDQKKIV	IPIIVIIISI	TLAIMYKYLV	FDRRKKLKRK	K-MKKVTDLF	GVNKTT----	----------	----------
PCHAS_030070	----------	----SEETPS	SSGVGTKKIG	IPIIVIIISI	TLAIMYKFLV	FDRRKKLKRK	K-MKKVPSLF	GVNKTT----	----------	----------
PCHAS_137110	KEPSSEDSTG	GQDAINIVPA	ASSLGEKKIG	IPIIVIIISI	TLVIMYKSKK	AHIKAYKFRL	WGKSSLLNIY	KLMQDTLHHL	LINFFC----	----------
PCHAS_114700	GTPPPETQHL	SSTTPPEEPP	TKPIDSKKIV	ISIIVILIPI	TLTILYKYLS	LGRRNELKKK	NNMKKVINMV	GVNKTTKTVI	NSSDGKKQIQ	IIIKSSSKKK
PCHAS_137030	GTPPPETQQL	SSTTPPEEPP	TKGMDRKKIV	ILIIVILIPI	TLTILYKYLS	SGWRKEMKKK	KNMTKVINMV	GVNKMTKMVI	NSSDGKKQIQ	IIIKSSSKKK
PCHAS_011480	RETPADIPSV	QDKAQETNPT	TSGMIDSITG	IPIILVLIPI	ILLIVCKYLS	SEWRKEMTRK	KNMTKVINVV	GVNKTTKMVI	NSSDGKKQIQ	IIIKSSSQKK
PCHAS_050040	KEPLPETQQS	SSTTPSEEPP	AKGVDTSITG	IPIILVLIPI	ILLIVCKYLS	SEWRREMTRK	KNMTKVINVV	GVNKTTKMVI	NSSDGKKQIQ	IIIKSSSQKK
PCHAS_000470	ESLPPETQQS	SSTTPPEDPP	KKGIDTSITG	IPIILVLIPI	ILLIVCKYLS	SEWRKEMTRK	KNMTKVINVV	GVNKTTKMVI	NSSDGKKQVQ	IIIKSSSKKK
PCHAS_000170	QASGTKTQSD	QGGLSGGSGS	SSGVSDKKIV	ISIIVILIPI	TLTILYKYLS	FGRRNELKKK	NNMKKVINMV	VANKTTKTVI	NSNDGKKQIK	IIIKSSSQKK
PCHAS_130170	EPPPPPPSGQ	LKNSQHETPP	SPGMDQKQFV	ILTIVFLIPI	ALAIMYQYLS	FGRRKKLKGK	KNMKKVINLF	GANKTTKTVI	NSSDGKKQIQ	IIIKSSGQKK
PCHAS_000660	KGRQPETQQL	SSTTPLEEPP	AKGIDQKKIV	ILIIVILIPI	TLTIMYKYLS	FGRRNELKKK	NNMKKVINMV	GVNKTTKTVI	NSSDGKKQIQ	IIIKSYSQKK
PCHAS_040080	EPPPASDNSE	GNTGSENRGP	SSGMDRKQFV	ILTIVLLIPI	ALAIIYKYLS	FGRRNKLKKK	NNMKKVINMV	GVNKTTKTVI	NSSDGKKQIQ	IIIKSYSQKK
PCHAS_070070	EPPPKEGASK	RTEHGGTSPP	GPGSDQKKIG	ISIIVILIPI	TLAILYKYLS	SGWRKEMKRK	KKMTKVINVA	GVNKTIKTVI	NSSDGKKQIQ	IIIKSSSQKK
PCHAS_137090	KESLDPSDGK	GSQVNGGDSE	NSGVPGKKIG	ISIIVILIPI	TLAILYTYLS	SGWRKEMKRK	KKMTKVINMV	GVNKTTKMVI	NSSDGKKQIQ	IIIKSSGQKK

Supplementary data 1 (CIR alignment)

```
              ....|....| ....|....| ....|....| ....|....| ....|....| ....|....| ....|....| ....|....| ....|....| ....|....|
                   405        415        425        435        445        455        465        475        485        495
PCHAS_000950  ---------- -----AVGTN PPKEL----- ---------- ---------- ---------- ---------- ---------- ---------- ----------
PCHAS_001010  ---------- -----VVGTN LPKEL----- ---------- ---------- ---------- ---------- ---------- ---------- ----------
PCHAS_140090  ---------- ---------- ---------- ---------- ---------- ---------- ---------- ---------- ---------- ----------
PCHAS_000730  ---------- ---------- ---------- ---------- ---------- ---------- ---------- ---------- ---------- ----------
PCHAS_070160  ---------- ---------- --KEKINH-- ---------- ---------- ---------- ---------- ---------- ---------- ----------
PCHAS_030020  ---------- ---------- ---------- ---------- ---------- ---------- ---------- ---------- ---------- ----------
PCHAS_000360  ---------- ---------- ---------- ---------- ---------- ---------- ---------- ---------- ---------- ----------
PCHAS_110020  ---------- ---------- ---------- ---------- ---------- ---------- ---------- ---------- ---------- ----------
PCHAS_050060  ---------- ---------- --KRKMNINI ---------- ---------- ---------- ---------- ---------- ---------- ----------
PCHAS_010030  ---------- ---------- ---------- ---------- ---------- ---------- ---------- ---------- ---------- ----------
PCHAS_030030  ---------- ---------- ---------- ---------- ---------- ---------- ---------- ---------- ---------- ----------
PCHAS_000350  ---------- ---------- --KKKMNHYI ---------- ---------- ---------- ---------- ---------- ---------- ----------
PCHAS_011530  ---------- ---------- --KKNVDHYM ---------- ---------- ---------- ---------- ---------- ---------- ----------
PCHAS_073160  ---------- ---------- --KKKMTNYV ---------- ---------- ---------- ---------- ---------- ---------- ----------
PCHAS_070090  ---------- ---------- --KKKMASYI ---------- ---------- ---------- ---------- ---------- ---------- ----------
PCHAS_000580  ---------- ---------- --KKKMELNI ---------- ---------- ---------- ---------- ---------- ---------- ----------
PCHAS_140130  ---------- ---------- --KTKMNDYI ---------- ---------- ---------- ---------- ---------- ---------- ----------
PCHAS_140040  ---------- ---------- --KSKEYNYI LFDESDYSRN SNNY------ ---------- ---------- ---------- ---------- ----------
PCHAS_000740  ---------- ---------- --KKKVYNYI LLEESDYSKN SNNY------ ---------- ---------- ---------- ---------- ----------
PCHAS_100030  ---------- ---------- --KRKEYNYI LFDESDYSRN SNND------ ---------- ---------- ---------- ---------- ----------
PCHAS_001120  ---------- ---------- --KRKEYNYI LFDESDYSRN SNNY------ ---------- ---------- ---------- ---------- ----------
PCHAS_011520  ---------- ---------- --KRKVYNYL LLEERDYSRN SNNY------ ---------- ---------- ---------- ---------- ----------
PCHAS_070030  ---------- ---------- --KRKVYNYL LLEERDYSRN SNND------ ---------- ---------- ---------- ---------- ----------
PCHAS_073190  ---------- ---------- --KKKVYNYI LLEESDYSRN SNNY------ ---------- ---------- ---------- ---------- ----------
PCHAS_000570  ---------- ---------- --KKKEYNYI LLEENDYSRN SNNY------ ---------- ---------- ---------- ---------- ----------
PCHAS_114730  ---------- ---------- --KRKVYNYL LLEERDYSRN SNNY------ ---------- ---------- ---------- ---------- ----------
PCHAS_010020  ---------- ---------- ---------- ---------- ---------- ---------- ---------- ---------- ---------- ----------
PCHAS_000280  ---------- ---------- ---------- ---------- ---------- ---------- ---------- ---------- ---------- ----------
PCHAS_000490  ---------- ---------- ---------- ---------- ---------- ---------- ---------- ---------- ---------- ----------
PCHAS_000030  ---------- ---------- --KKKMNNYI ---------- ---------- ---------- ---------- ---------- ---------- ----------
PCHAS_000090  ---------- ---------- --KKKMNNYI ---------- ---------- ---------- ---------- ---------- ---------- ----------
PCHAS_000270  ---------- ---------- --KKKMNNYI ---------- ---------- ---------- ---------- ---------- ---------- ----------
PCHAS_000120  ---------- ---------- --KKKMNRYV ---------- ---------- ---------- ---------- ---------- ---------- ----------
PCHAS_001090  ---------- ---------- --KKKMKQYI ---------- ---------- ---------- ---------- ---------- ---------- ----------
PCHAS_011510  ---------- ---------- --KNKMELNI ---------- ---------- ---------- ---------- ---------- ---------- ----------
PCHAS_070050  ---------- ---------- ---------- ---------- ---------- ---------- ---------- ---------- ---------- ----------
PCHAS_000720  ---------- ---------- ---------- ---------- ---------- ---------- ---------- ---------- ---------- ----------
PCHAS_070020  ---------- ---------- --KRKEYNYI LFDESDYSRN SNNY------ ---------- ---------- ---------- ---------- ----------
PCHAS_104260  ---------- ---------- --KRKVYNYI ---------- ---------- ---------- ---------- ---------- ---------- ----------
PCHAS_140020  ---------- ---------- --KRKEYNYI LFDESDYSRN SNNY------ ---------- ---------- ---------- ---------- ----------
PCHAS_030060  ---------- ---------- --TKKMELNI ---------- ---------- ---------- ---------- ---------- ---------- ----------
PCHAS_000750  ---------- ---------- --KNKMELNI ---------- ---------- ---------- ---------- ---------- ---------- ----------
PCHAS_000680  ---------- ---------- ---------- ---------- ---------- ---------- ---------- ---------- ---------- ----------
```

Supplementary data 1 (CIR alignment)

```
PCHAS_000420   ---------- ---------- --KRKVYNYL LLEERDYSRN SNNY------ ---------- ---------- ---------- ---------- ----------
PCHAS_000100   ---------- ---------- --KRKVYNYI LLEERDYSRN SNNY------ ---------- ---------- ---------- ---------- ----------
PCHAS_000310   ---------- ---------- --KRKVYNYI LLEENDYSRN SNNY------ ---------- ---------- ---------- ---------- ----------
PCHAS_040040   ---------- ---------- --KSKEYNYI LFDESDYSRN SNNY------ ---------- ---------- ---------- ---------- ----------
PCHAS_120060   ---------- ---------- --KSKEYNYI LFDESDYSRN SNNY------ ---------- ---------- ---------- ---------- ----------
PCHAS_104230   ---------- ---------- --KRKVYNYL LLEERDYSRN SNNY------ ---------- ---------- ---------- ---------- ----------
PCHAS_030040   ---------- ---------- --KRKVYNYL LLEESDYSRN SNNY------ ---------- ---------- ---------- ---------- ----------
PCHAS_000340   ---------- ---------- --KRKVYNYL LLEERDYSRN SNNY------ ---------- ---------- ---------- ---------- ----------
PCHAS_001110   ---------- ---------- --KKKMELNI ---------- ---------- ---------- ---------- ---------- ---------- ----------
PCHAS_120040   ---------- ---------- --KKKMELNI ---------- ---------- ---------- ---------- ---------- ---------- ----------
PCHAS_000130   ---------- ---------- ---------- ---------- ---------- ---------- ---------- ---------- ---------- ----------
PCHAS_000400   ---------- ---------- --TKKMELNI ---------- ---------- ---------- ---------- ---------- ---------- ----------
PCHAS_040020   ---------- ---------- --KKKMELNI ---------- ---------- ---------- ---------- ---------- ---------- ----------
PCHAS_000110   ---------- ---------- --KKKMNRYI ---------- ---------- ---------- ---------- ---------- ---------- ----------
PCHAS_120050   ---------- ---------- --E------- ---------- ---------- ---------- ---------- ---------- ---------- ----------
PCHAS_104250   ---------- ---------- --KKKMNDYI ---------- ---------- ---------- ---------- ---------- ---------- ----------
PCHAS_000320   ---------- ---------- --KKKMNDYI ---------- ---------- ---------- ---------- ---------- ---------- ----------
PCHAS_000410   ---------- ---------- --KKKMNDYI ---------- ---------- ---------- ---------- ---------- ---------- ----------
PCHAS_140030   ---------- ---------- --KKKMNRYI ---------- ---------- ---------- ---------- ---------- ---------- ----------
PCHAS_114740   ---------- ---------- --KKKMNRYI ---------- ---------- ---------- ---------- ---------- ---------- ----------
PCHAS_040030   ---------- ---------- --KKKMNHYI ---------- ---------- ---------- ---------- ---------- ---------- ----------
PCHAS_070130   ---------- ---------- --KKKMNHYI ---------- ---------- ---------- ---------- ---------- ---------- ----------
PCHAS_073180   ---------- ---------- --KRREYNYI LFEESDYSRN SNNY------ ---------- ---------- ---------- ---------- ----------
PCHAS_000300   ---------- ---------- --KKKVYNYI LFEESDYSRN SNNY------ ---------- ---------- ---------- ---------- ----------
PCHAS_120070   ---------- ---------- --KRKEYNYI ---------- ---------- ---------- ---------- ---------- ---------- ----------
PCHAS_001130   ---------- ---------- --KRKEYNYI LFEESDYSRN SNNY------ ---------- ---------- ---------- ---------- ----------
PCHAS_100040   ---------- ---------- --KRKEYNYI LFEESDYSRN SNNY------ ---------- ---------- ---------- ---------- ----------
PCHAS_000560   ---------- ---------- --KRKEYNYI LFEESDYSRN SNNY------ ---------- ---------- ---------- ---------- ----------
PCHAS_050020   ---------- ---------- --KNKMASYV ---------- ---------- ---------- ---------- ---------- ---------- ----------
PCHAS_000020   ---------- ---------- --KNKMASYV ---------- ---------- ---------- ---------- ---------- ---------- ----------
PCHAS_000290   ---------- ---------- --KNKMASYV ---------- ---------- ---------- ---------- ---------- ---------- ----------
PCHAS_060020   ---------- ---------- --KNKINHYI ---------- ---------- ---------- ---------- ---------- ---------- ----------
PCHAS_001040   ---------- ---------- --KKKMNINI ---------- ---------- ---------- ---------- ---------- ---------- ----------
PCHAS_114750   ---------- ---------- --KKKMNINI ---------- ---------- ---------- ---------- ---------- ---------- ----------
PCHAS_000140   ---------- ---------- --KKKMNINI ---------- ---------- ---------- ---------- ---------- ---------- ----------
PCHAS_000070   ---------- ---------- --KKKMNLSM ---------- ---------- ---------- ---------- ---------- ---------- ----------
PCHAS_030080   ---------- ---------- --KKKLNHYI ---------- ---------- ---------- ---------- ---------- ---------- ----------
PCHAS_073200   ---------- ---------- --KKKMNNYI ---------- ---------- ---------- ---------- ---------- ---------- ----------
PCHAS_120020   ---------- ---------- --KKKMASYV ---------- ---------- ---------- ---------- ---------- ---------- ----------
PCHAS_000060   ---------- ---------- --KKKMASYV ---------- ---------- ---------- ---------- ---------- ---------- ----------
PCHAS_130020   ---------- ---------- --KKKINLNI ---------- ---------- ---------- ---------- ---------- ---------- ----------
PCHAS_000150   ---------- ---------- --NKKMASYV ---------- ---------- ---------- ---------- ---------- ---------- ----------
PCHAS_000500   ---------- ---------- -RDKK----- ---------- ---------- ---------- ---------- ---------- ---------- ----------
PCHAS_001100   ---------- ---------- -RDKK----- ---------- ---------- ---------- ---------- ---------- ---------- ----------
PCHAS_042010   ---------- ---------- DDKNKSIKME INSRDEKQKT HITINSGYTK KYTKSVINPG DGKKNPLLNI YKLMQANPIL FINLFFLLIF FVYKRKGSTI
PCHAS_000040   ---------- ---------- -SDKK----- ---------- ---------- ---------- ---------- ---------- ---------- ----------
PCHAS_000390   ---------- ---------- -SDKK----- ---------- ---------- ---------- ---------- ---------- ---------- ----------
```

Supplementary data 1 (CIR alignment)

```
PCHAS_030180   ---------- ---------- -CVKNKIKRN LKYIIENNQR GQL------- ---------- ---------- ---------- ---------- ----------
PCHAS_130070   ---------- ---------- -CVKNKIKRN LKYIIENNQR GQL------- ---------- ---------- ---------- ---------- ----------
PCHAS_011500   ---------- ---------- ---DEKGYKF V--------- ---------- ---------- ---------- ---------- ---------- ----------
PCHAS_146850   ---------- ---------- -CDVNKTKKE VTNGSA---- ---------- ---------- ---------- ---------- ---------- ----------
PCHAS_070100   ---------- ---------- -CDANKIQKD VTNGFVENI- ---------- ---------- ---------- ---------- ---------- ----------
PCHAS_060050   ---------- ---------- -DQKKAQNGF TNAFIEKNQS E--------- ---------- ---------- ---------- ---------- ----------
PCHAS_030110   ---------- ---------- V--------- ---------- ---------- ---------- ---------- ---------- ---------- ----------
PCHAS_130050   ---------- ---------- -NDQKKA--- ---------- ---------- ---------- ---------- ---------- ---------- ----------
PCHAS_030090   ---------- ---------- -SDQKRA--- ---------- ---------- ---------- ---------- ---------- ---------- ----------
PCHAS_146870   ---------- ---------- -SDQKKA--- ---------- ---------- ---------- ---------- ---------- ---------- ----------
PCHAS_130030   ---------- ---------- -SDQKKA--- ---------- ---------- ---------- ---------- ---------- ---------- ----------
PCHAS_040110   ---------- ---------- -SDQKKA--- ---------- ---------- ---------- ---------- ---------- ---------- ----------
PCHAS_042030   ---------- ---------- -SDQKKA--- ---------- ---------- ---------- ---------- ---------- ---------- ----------
PCHAS_060140   ---------- ---------- -SDQKKA--- ---------- ---------- ---------- ---------- ---------- ---------- ----------
PCHAS_030210   ---------- ---------- -SDQKKA--- ---------- ---------- ---------- ---------- ---------- ---------- ----------
PCHAS_060060   ---------- ---------- -SDQKKA--- ---------- ---------- ---------- ---------- ---------- ---------- ----------
PCHAS_050070   ---------- ---------- -SDQKKA--- ---------- ---------- ---------- ---------- ---------- ---------- ----------
PCHAS_040050   ---------- ---------- -CDENKTKKC VKNAFIEKNQ SE-------- ---------- ---------- ---------- ---------- ----------
PCHAS_001050   ---------- ---------- -SDQKKA--- ---------- ---------- ---------- ---------- ---------- ---------- ----------
PCHAS_083760   ---------- ---------- -SDQKKA--- ---------- ---------- ---------- ---------- ---------- ---------- ----------
PCHAS_140070   ---------- ---------- -SDQKKA--- ---------- ---------- ---------- ---------- ---------- ---------- ----------
PCHAS_000180   ---------- ---------- -SDQKKA--- ---------- ---------- ---------- ---------- ---------- ---------- ----------
PCHAS_001060   ---------- ---------- -SDQKKA--- ---------- ---------- ---------- ---------- ---------- ---------- ----------
PCHAS_011450   ---------- ---------- -SDPKKA--- ---------- ---------- ---------- ---------- ---------- ---------- ----------
PCHAS_060030   ---------- ---------- -CDEKKA--- ---------- ---------- ---------- ---------- ---------- ---------- ----------
PCHAS_120030   ---------- ---------- -SDQKKA--- ---------- ---------- ---------- ---------- ---------- ---------- ----------
PCHAS_030120   ---------- ---------- -CDKKKAKNG VTNVFIEKNQ LE-------- ---------- ---------- ---------- ---------- ----------
PCHAS_114640   ---------- ---------- -CEEK----- ---------- ---------- ---------- ---------- ---------- ---------- ----------
PCHAS_130350   ---------- ---------- IGGKRSVKII INPSTQKKQT KKSKN----- -------SVR GEKMSSLNMY ELMKADPLPF INLFFLMIFF VYKRTRDSIE
PCHAS_030150   -------LDR KKTKKIDINS IDGKKTIQIT INSNDKKKET KKIIS----- -------SDN GKTTLLFNIY KQMQLSPMPF IHLFMLLIFF IFKRKKDSIE
PCHAS_060120   -------NDK KKTPKRIINS NDKEKTRKRI INSNDKGKTR KRIIN----- ------YNDK EKTTLLFNIY KQMRLSPMPF IHLFMLLILF IFKRKKNSIE
PCHAS_041960   ---------- KKTTKKIIDS NDKEKTPKRI INSNDKEKTA KRIIS----- -------SNN GKTTLLFNIY KQMQLSPMPF IHLFMLLIFF IFKRKKDSIE
PCHAS_130110   ---------- ------IINS NDKKKPKKRI ISSNDKEKTT KRNIS----- -------SDN GKASLLFNIY KKMQLSPMPF IHLFMLLIFF IFKRKKDFIE
PCHAS_041950   ---------- --------VG VDKTKKTAIN ---------- ---------- ---------- ---------- ---------- ---------- ----------
PCHAS_060130   -------VEI NKTKKTVINS INGKRPMQII INSSTKKKQT KKFIT----- -------SVY GKNFPLLNIY NLMQADPVPF INLFFLLIFF VYK-------
PCHAS_130120   -------VEI NKTKKTVINS INGKRPMQII ISSSTKKKQT KKIIT----- -------SVY GKNFPLLNIY KLMEADPLPF INLFFLLIFF VYKRKDILLN
PCHAS_070170   -------VDK KKSIKKGINS HDGKRKTHIT INSKHKKKST KMVIN-LDDG KKTKIIVNSV NEKMPSLNIY KLMQADPMPF INLFLLWIFF IYKRKRDTIE
PCHAS_100060   -------VDK KKSIEKGINS RDGKQKTHII INSKHKKKNT KMIIN-LGDG KKTKIIVNSV NEKMPSLNIY KLMQADPMPF INLFLLWIFF IYKRKRDTIE
PCHAS_146770   -------DDK NKSIKHEIYS HDGKRKTHIT INSGYKKKNT KSVIN-SDDR KTTKIIVNSV NEKIPLLNIY KLMHADPMPF INLFFLLIFF VYKRKRDTIE
PCHAS_146790   ---------- ---------- TSGKKQIQII INSSTKKKQT KKYIK----- -------PVY RGKPPLLNIY KLMQADPMPF INLFFLLIFF VYKRKRDTIE
PCHAS_060160   -------VNE KKSIKTVINS RDGKRKTYIT INSEYKKKYT KSVIN-FGDG KKTEIVINSV NVKIPLLNIY KLMQADPMPF INLFFLLIFF IYKRKRDTIE
PCHAS_000260   -------DDN KKSIKTVINS RNGKRKTYIT INSEYKKKYT KSVIN-SGDG KKTEIIINSV NVKIPLLNIY KLMQADPMPF INLFFLLIFF IYKRKRDTIE
PCHAS_130280   -------VNE KKSIKTVINS RDGKRKTYIT INSEYKKKYT KSVIN-FGDG KKTEIVINSV NVKIPLLNIY KLMQADPMPF INLFFLWIFF IYKRKRDTIE
PCHAS_083720   -------VNE KKSIKTVINS RDGKRKTYIT INSEYKKKYK KSVIN-FGDG KKTEIVINSV NVKIPLLNIY KLMQADPMPF INLFFLWIFF IYKRKRDTIE
PCHAS_090010   ---------- ---------- TSGKKQIQII IKSSTKKKQT KKSIK----- -------PVY REKSPLLNIY KLMQADPMPF INLFFLLIFF VYKRKRDIIE
PCHAS_114600   ---------- ---------- TSGKRQIQII INSSTKKKQT KKSIK----- -------PVY RGKPPLLNIY KLMQADPMPF INLFFLLIFF VYKRKRDTIE
PCHAS_146860   IAKIIINDDM DEPIKTVINP WNEKRKTHAT INSEYKKKYI KSVIN-SGP- ---------- ---------- ---------- ---------- ----------
```

Supplementary data 1 (CIR alignment)

```
PCHAS_130090   IAKIIINDDT NKPIKTEINS RDEKRKTHIT INSEHAKKYT KSVIN-SSDR KKRKIIVNSV NEKMPLLNIY KLMKADPIPF INLFFLLIFF VYKRKQDTI-
PCHAS_030170   LAKIIINDDN INPIKKVINP WDEKQMTDIT INSENTKKYT KSVIN-SGDR KKTKIIVNSV NEKISLLNIY KFMKADPMPF INLFFLLIFF VYKRKRNTIE
PCHAS_130060   IAKIIMNDDT NKPIKTAINP WDEKRMTHIT INSEHTKKYT KSVIN-SGDR KKTKIIVNSV NEKITLLNIY KLMKADPMPF INLFFLVIFF VYKRKRNTIE
PCHAS_030200   IAKIIMNDDN IKPIKEAINP WDEKQKTDIT INSEHTKKYT KSVIN-SGDR KKTKIIVNSV NEKISLLNIY KFMKADPMPF INLFFLLIFY VYKRKRNTIE
PCHAS_060080   IAKIIMNDDN IKPIKKAINP WDEKRMTHIT INSEHTKKHT KSVIN-SSDR KKTKIIANSV NEKISLLNIY KFMKADPMPF INLFFLLIFF VYKRKRSTIE
PCHAS_060110   IAKIIMNDDN IKPIKKAIDP WDEKQMTHIT INSEHTKKYT KSVIN-SSDR KKTNIIANSV NEKISLLNIY KFMKADPIPF INLFFLLIFF VYKRKYNFL-
PCHAS_030140   IAKIIMNDDN IKPIKKAIDP WDEKQMTHIT INSEHTKKYT KSVIN-SSDR KKTKIIANSV NEKISLLNIY KFMKADPMPF INLLFLLIFF VYKRKRNTIE
PCHAS_130100   IAKIIMNDDN IKPIKKAIDP WDEKQMTHIT INSEHTKKYT KSVIN-SSDR KKTKIIANSV NEKISLLNIY KFMKADPIPF INLFFLLIFF VYKRKYNFL-
PCHAS_041990   TSNTIISIDD ENTTNTIISP NYEETNVKTI IDSDSGEKTT IVIINSYDEK NITIHSIKSS PPKITALNAY KHIFTNPAPF ISLFFLLIFF FYK-------
PCHAS_130080   TSNIIISIDE ENTENATISP NCEETNVKTI INSDCEKKTT IVIINSYDEK DVTIQSVKSS SPKTTSLKGY KHIFANPAPF INLFFLLIFF VYKKKYNFL-
PCHAS_030190   TSNIIISIDE ENTENATISP NCEETNVKTT INSDCEKKTT IVIINSYDEK DVTIQSVKSS SPKTTSLKGY KHIFANPAPF INLFFLLIFF VYKRKYNFL-
PCHAS_060090   ISNTIISIDE KNTENATISP NSEETNVKTT IDSDCEEKTT ILIINSYDEK DVTIQSVKSS SPKTTSLKGY KHIFANPAPF INLFFLLIFF VYKRKYNFL-
PCHAS_041970   ITIQNIK--- ---------- ---------- ---------- ---------- --------FS SPKTTSLNAY KHIFSNSAPF INLFFLLIFF VYKRKYNSL-
PCHAS_041980   TATTVIN--- ---------- ---------- ---------- -----LIGGK NIMIQSIKSS FHQAISLNAY KHIFSNSAPF INLFFLLIFF VYKGKYISL-
PCHAS_042020   IAKIIIN--- ---------- ---------- ---------- ---------- -------PGD GKKNPLLNIY KLMQADPMPF INLFFLLIFF VYKRKSTIE-
PCHAS_060070   IAKIIIN--- ---------- ---------- ---------- -----SGP-- ---------- ---------- ---------- ---------- ----------
PCHAS_011330   QTKKSIN--- ---------- ---------- ---------- ---------- --------SV NEKIPLLNIY KLMQADPVPF INLFFLLIFF VYKRKRDVIE
PCHAS_000220   QTKKSIN--- ---------- ---------- ---------- ---------- --------LV NEKIPLLNIY KLMQADPAPF INLFFLLIFF VYKRKDNSIE
PCHAS_042070   QSKKFIT--- ---------- ---------- ---------- ---------- -------SVY RKNFPLLNIY TLMEADPVPF INLFFLLIFC VYKRKLNYFE
PCHAS_110030   NLKPVIN--- ---------- ---------- ---------- ---------- -------SVD RKKDSLLNIH KLMQADSIPF INLFFLLIFL SIKENTIFWN
PCHAS_140140   DLKPVIN--- ---------- ---------- ---------- ---------- -------SVD RKKDSLLNIP KLMQANSIPF INLFFLLIFL SIKENTIFWN
PCHAS_010040   DLKPVIN--- ---------- ---------- ---------- ---------- -------SVS RKKYPLLNIY KLMQADPVPF INLFFLLIFF VYKENMIL--
PCHAS_073150   DLKPVIN--- ---------- ---------- ---------- ---------- -------PVG RKKRPLLDIY KLMQADPIPF INIFFLLIFF VYKRKYDFLE
PCHAS_073130   QIKKSIN--- ---------- ---------- ---------- ---------- -------PVY RERFPLLNIY KLMQADPVPF INLFFLLTFL FIKEKTILWN
PCHAS_040060   QTKKSIN--- ---------- ---------- ---------- ---------- -------PVY GEKSPSINIY KLMQADPVPF INLIFLLIFL FIKEKTILWK
PCHAS_000430   QTKKSIN--- ---------- ---------- ---------- ---------- -------PVY GEKSPSINIY KLIQADPVPF INLIFLVIFL FIKEKTILWK
PCHAS_104200   QTKKSIN--- ---------- ---------- ---------- ---------- -------PVY GEKSPSINIY KLMQADPVPF INLIFLLIFL FIKEKTILWK
PCHAS_114720   QTKKSIN--- ---------- ---------- ---------- ---------- -------PVY VEKSPSINIY KLMQADPVPF INLIFLVIFL FIKEKTIFWK
PCHAS_000770   QTKKSIN--- ---------- ---------- ---------- ---------- -------FVN RKKPPFLNIY KLMMADAIPF INLFFLLIFF VYKRKRNTIE
PCHAS_130220   KTKKSIN--- ---------- ---------- ---------- ---------- -------SIY GEKFPLVNVY KLMQADPAPF INLFFLLIFF VYKRKENTIE
PCHAS_070040   ---------- ---------- ---------- ---------- ---------- ---------- ---------- ---------- ---------- ----------
PCHAS_011490   ---------- ---------- ---------- ---------- ---------- ---FVDFFVI FLLIFFVDFF VIFLLIFFVI FFVNFFCQFF LFIKENETL-
PCHAS_070060   ---------- ---------- ---------- ---------- ---------- ---------- ---------- ---------- ---------- ----------
PCHAS_030070   ---------- ---------- ---------- ---------- ---------- ---------- ---------- ---------- ---------- ----------
PCHAS_137110   ---------- ---------- ---------- ---------- ---------- ---------- ---------- ---------- ---------- ----------
PCHAS_114700   KTKKSIN--- ---------- ---------- ---------- ---------- -------SVY GEKSLSLKIY QLMQADPVPF INLIFLLIFF VYKRKRDFIE
PCHAS_137030   KTKKSIN--- ---------- ---------- ---------- ---------- -------SVY GEKSPSLNIY QLMQADPVPF INLFFLLIFF VYKRKRDFIE
PCHAS_011480   QTKKSIN--- ---------- ---------- ---------- ---------- -------SVN RKKSPSLNMY QLMQADPVPF INLIFLLIFF VYKRKRDFIE
PCHAS_050040   QTKKSIN--- ---------- ---------- ---------- ---------- -------FVY GEKSPSLKIY QLMQADPVPF INLIFLLIFF VYKRKRDFIE
PCHAS_000470   KTKKSIN--- ---------- ---------- ---------- ---------- -------SVY GEKSPSLNIY QLMQADPVPF INLIFLLIFF VYKRKRDFIE
PCHAS_000170   QTKKFIN--- ---------- ---------- ---------- ---------- -------FVY GEKSPSLNIY QLMQADPVPF INLFFLLIFF VYKRKRDFIE
PCHAS_130170   QTIKSIN--- ---------- ---------- ---------- ---------- -------SVY GEKSPSLNIY QLMQADPVPF INLFFLLIFF VYKRKENTIE
PCHAS_000660   KTKKFIN--- ---------- ---------- ---------- ---------- -------FVY GEKSPSLNIY QLMQADPVPF INLIFLLIFF VYKRKRDFIE
PCHAS_040080   PTKKSIN--- ---------- ---------- ---------- ---------- -------SVN RKKSPSLNIY QLMQADPVPF INLFFLLIFF VYKRKRDFIE
PCHAS_070070   QTKKSIN--- ---------- ---------- ---------- ---------- -------SVN RKKSPSLNIY QLMQADPVPF INLIFLLIFF VYKRKRDFIE
PCHAS_137090   QTKKSIN--- ---------- ---------- ---------- ---------- -------SVN RKKSSSLNIY QLMQADPVPF INLFFLLIFF VYKRKRDFIE
```

b) CIRs excluded from the amino acid sequence alignment:

**Reason for exclusion**	***cir* genes**
Partial *cir* gene	PCHAS_000080<br>PCHAS_000330<br>PCHAS_001070
Poor alignment	PCHAS_042000<br>PCHAS_010120<br>PCHAS_030160<br>PCHAS_030270<br>PCHAS_060100<br>PCHAS_100110<br>PCHAS_104110<br>PCHAS_093820<br>PCHAS_083690<br>PCHAS_104270
CIR-like genes	PCHAS_146840<br>PCHAS_090030<br>PCHAS_052480
