# Supplementary figures and images for: Characterization and gene expression analysis of the cir multi-gene family of plasmodium chabaudi chabaudi (AS)
Source: BMC Genomics. 2012 Mar 29;13:125. doi: 10.1186/1471-2164-13-125 (PMC3384456; doi:10.1186/1471-2164-13-125)

**Supplementary data 2 (Conservation plot of CIR alignment)**

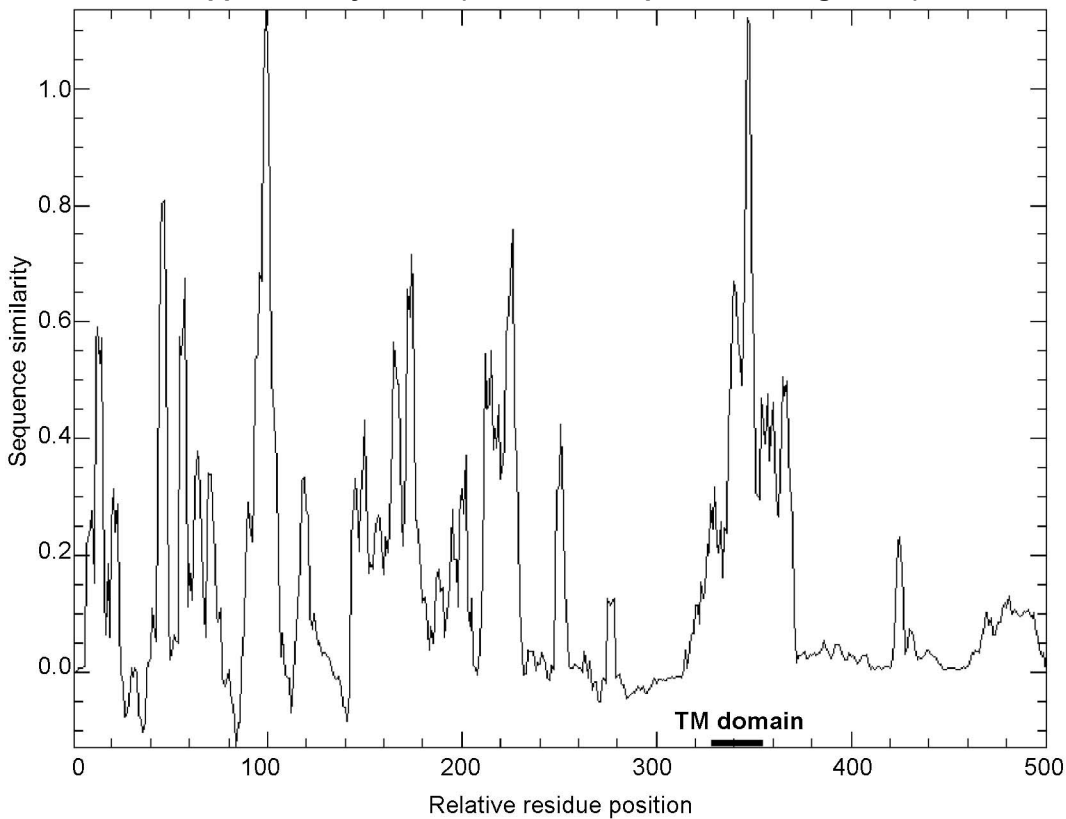

Supplement: Additional file 2 — CIR alignment conservation. [file 1471-2164-13-125-S2.PDF]

# Supplementary data 3 (Network with bootstrap support values)

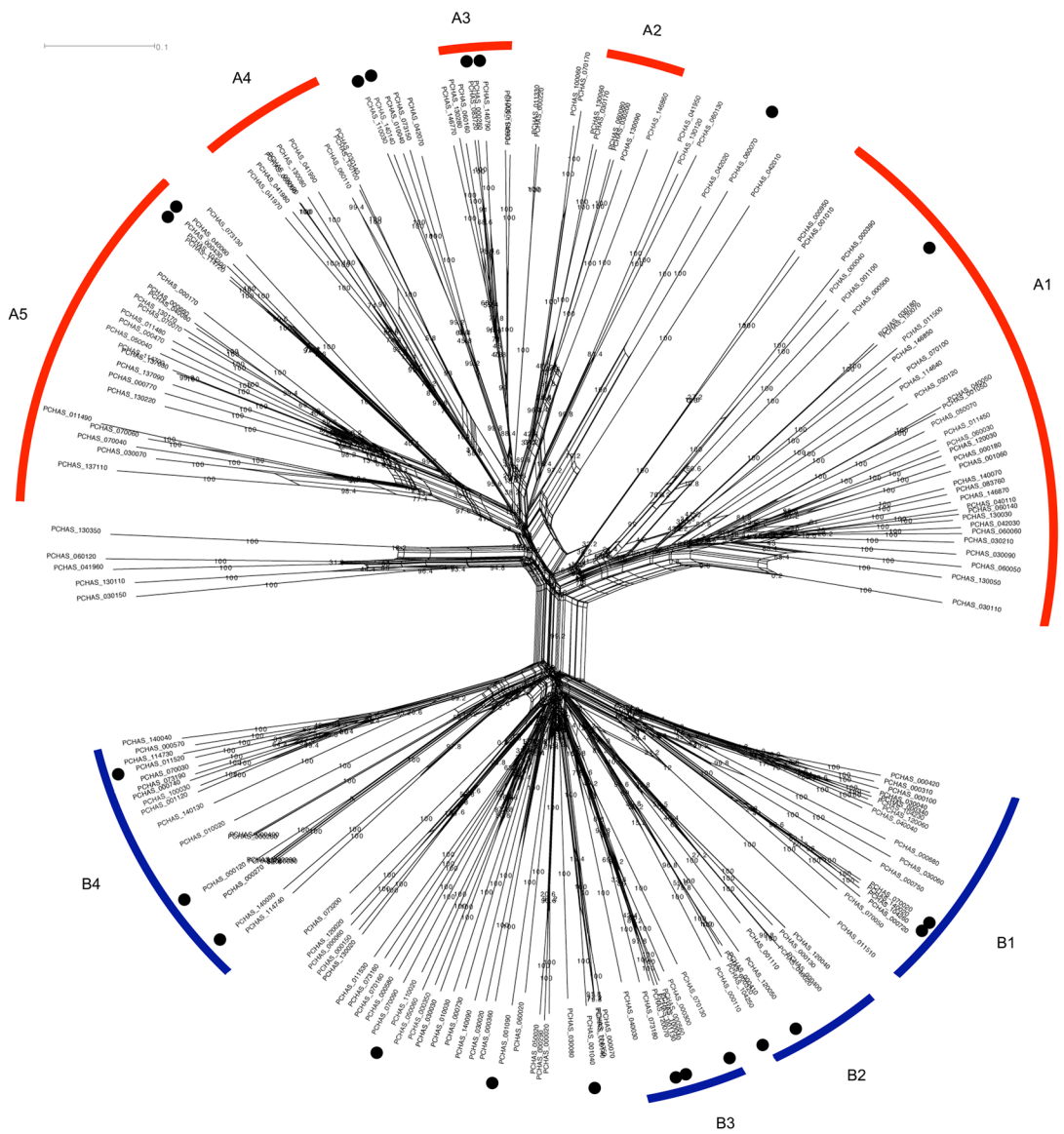

Supplement: Additional file 3 — CIR network showing bootstrap values. [file 1471-2164-13-125-S3.PDF]

Supplementary data 4 (ML tree)

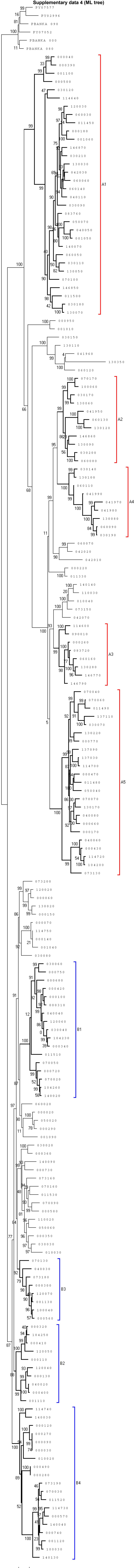

Supplement: Additional file 4 — Maximum likelihood tree of CIR sequences. [file 1471-2164-13-125-S4.PDF]

*P. chabaudi* in BALB/c mice

A

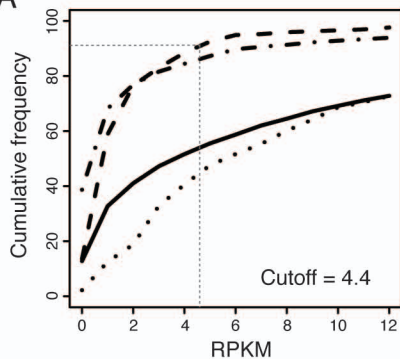

B

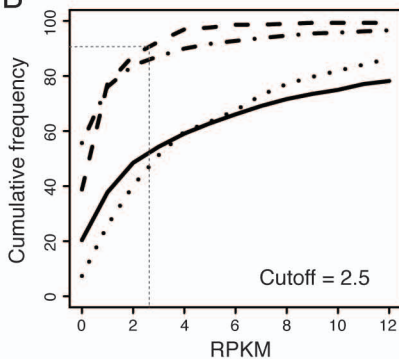

C

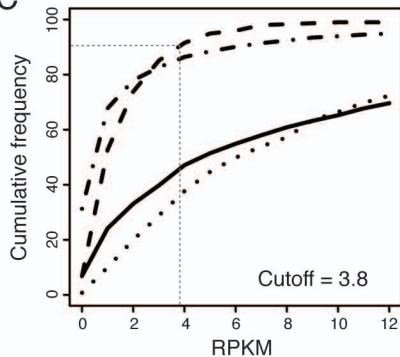

D

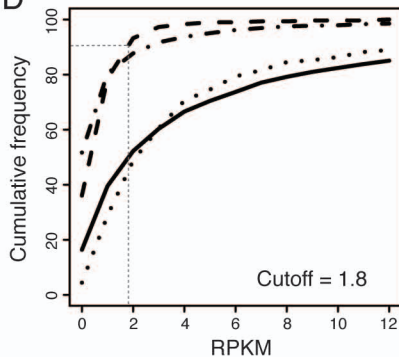

*P. chabaudi* in C57BL/6 mice

D

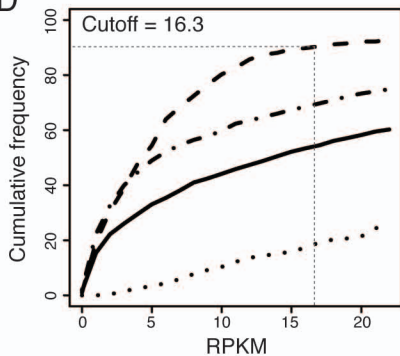

E

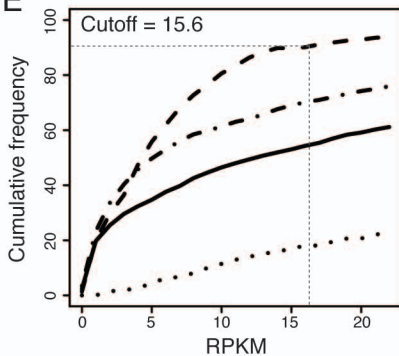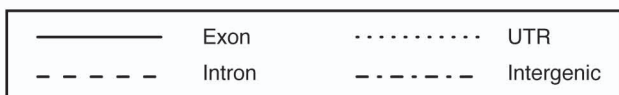

Supplement: Additional file 10 — cir gene expression threshold of detection determination. [file 1471-2164-13-125-S10.PDF]
