# Supplementary material for: Characterization and gene expression analysis of the cir multi-gene family of plasmodium chabaudi chabaudi (AS)
Source: BMC Genomics. 2012 Mar 29;13:125. doi: 10.1186/1471-2164-13-125 (PMC3384456; doi:10.1186/1471-2164-13-125)
Supplement: Additional file 5 — Sub-families identified from the alignment of 183 CIRs. [file 1471-2164-13-125-S5.PDF]

CIR sub-family members

A1	A2	A3	A4	A5	B1	B2	B3	B4
PCHAS_000040	PCHAS_130060	PCHAS_060160	PCHAS_030140	PCHAS_040060	PCHAS_011510	PCHAS_000400	PCHAS_120070	PCHAS_140030
PCHAS_000390	PCHAS_030170	PCHAS_130280	PCHAS_130100	PCHAS_000430	PCHAS_104260	PCHAS_040020	PCHAS_001130	PCHAS_114740
PCHAS_001100	PCHAS_060080	PCHAS_146790	PCHAS_041970	PCHAS_104200	PCHAS_140020	PCHAS_120040	PCHAS_100040	PCHAS_000030
PCHAS_000500	PCHAS_030200	PCHAS_000260	PCHAS_060110	PCHAS_114720	PCHAS_070020	PCHAS_000130	PCHAS_000560	PCHAS_000090
PCHAS_030120	PCHAS_130090	PCHAS_083720	PCHAS_041990	PCHAS_073130	PCHAS_000720	PCHAS_001110	PCHAS_000300	PCHAS_000270
PCHAS_114640	PCHAS_146860	PCHAS_090010	PCHAS_030190	PCHAS_030070	PCHAS_070050	PCHAS_000110	PCHAS_073180	PCHAS_000120
PCHAS_030180	PCHAS_100060	PCHAS_114600	PCHAS_041980	PCHAS_137110	PCHAS_030060	PCHAS_120050	PCHAS_070130	PCHAS_000280
PCHAS_130070	PCHAS_070170	PCHAS_146770	PCHAS_130080	PCHAS_011490	PCHAS_000750	PCHAS_104250	PCHAS_040030	PCHAS_000490
PCHAS_146850	PCHAS_041950		PCHAS_060090	PCHAS_070060	PCHAS_000680	PCHAS_000320		PCHAS_010020
PCHAS_011500	PCHAS_130120			PCHAS_070040	PCHAS_000100	PCHAS_000410		PCHAS_140130
PCHAS_070100	PCHAS_060130			PCHAS_011480	PCHAS_000310			PCHAS_000570
PCHAS_030110				PCHAS_000470	PCHAS_000420			PCHAS_114730
PCHAS_130050				PCHAS_050040	PCHAS_040040			PCHAS_140040
PCHAS_040050				PCHAS_000770	PCHAS_120060			PCHAS_100030
PCHAS_001050				PCHAS_130220	PCHAS_104230			PCHAS_001120
PCHAS_050070				PCHAS_114700	PCHAS_030040			PCHAS_000740
PCHAS_140070				PCHAS_137030	PCHAS_000340			PCHAS_011520
PCHAS_000180				PCHAS_000170				PCHAS_070030
PCHAS_001060				PCHAS_130170				PCHAS_073190
PCHAS_011450				PCHAS_070070				
PCHAS_060030				PCHAS_000660				
PCHAS_120030				PCHAS_040080				
PCHAS_030210				PCHAS_137090				
PCHAS_146870								
PCHAS_083760								
PCHAS_060050								
PCHAS_030090								
PCHAS_060060								
PCHAS_130030								
PCHAS_042030								
PCHAS_040110								
PCHAS_060140								
