# Supplementary material for: Characterization and gene expression analysis of the cir multi-gene family of plasmodium chabaudi chabaudi (AS)
Source: BMC Genomics. 2012 Mar 29;13:125. doi: 10.1186/1471-2164-13-125 (PMC3384456; doi:10.1186/1471-2164-13-125)
Supplement: Additional file 6 — Detection of phylogenetic incompatibilities between cir genes. [file 1471-2164-13-125-S6.PDF]

Supplementary data 6 (Detection of phylogenetic inconsistencies)

a) i

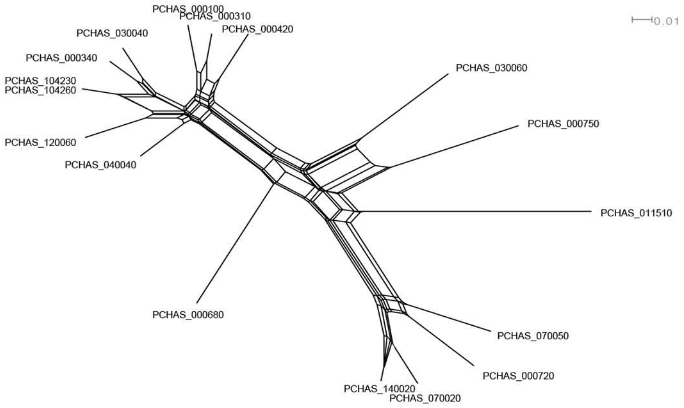

ii

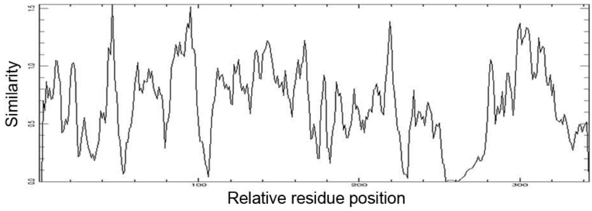

iii

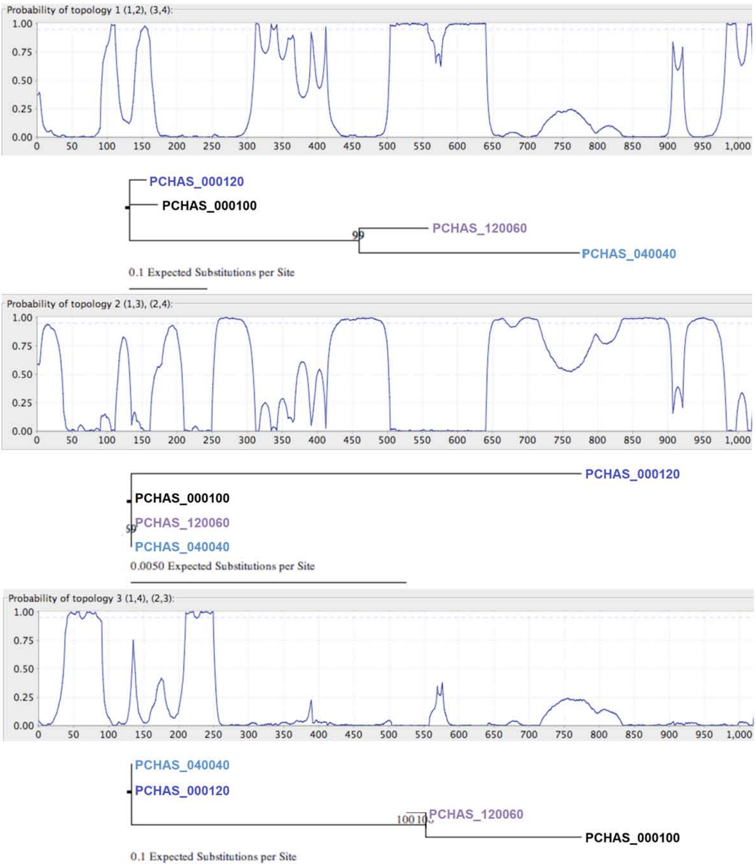

iv

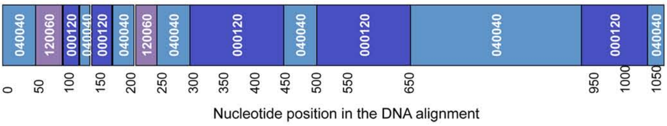

b) Sub-family B1 was chosen as an example since this appeared to be the most conserved of CIR clades, with a longer branch separating this from the other families within sub-type B CIRs (supplementary data 3 and 4). The members of this sub-family were aligned, and a network generated, as described previously (Figure 1), i).

Four *cir* DNA sequences were chosen from within clade B1: PCHAS_000100, PCHAS _000310, PCHAS _120060 and PCHAS _040040. These sequences retained a significant probability of recombination (P = 0.0) using the PHI test within Splitstree (39), when the other *cir*s were removed. The *cir* quartet was aligned as previously described for CIR sequences, a) ii). The program 'tree TOPology-related analysis of ALignments interface' [TOPALi (46,47)] was used to produce a phylogenetic profile along the length of these sequences, iteratively removing sequence information from either the 5' or 3' end. By this approach, the tree topology would change when a recombination break-point was exposed.

One such profile is shown in a) iii), indicating the regions of the *cir* alignment where one of three possible phylogenetic relationships was predicted. Phylogenetic inconsistencies could be observed between the four *cir* sequences, suggesting that different regions of the genes display different relationships with each other. PCHAS_000100 is shown as an example in a) iv). As the PCHAS_000100 gene contained similarity to different *cir*s along its length, it is likely that this *cir* has undergone several recombination events.

c) Tabulated scores from the whole repertoire, using quartets selected at random from each *cir* sub-family

***cir* sub-family**	**Quartet:**				
	**1**	**2**	**3**	**4**	**5**
A1	0.40 (48)	**0.00** (36)	1.00 (11)	0.42 (54)	**0.04** (35)
A2	**0.00** (18)	0.28 (12)	**0.00** (29)	**0.00** (32)	0.58 (31)
A3	**0.00** (34)	0.84 (19)	**0.00** (35)	**0.03** (34)	**0.00** (29)
A4	1.00 (18)	**0.01** (53)	0.69 (36)	**0.00** (32)	**0.01** (0)
A5	**0.01** (33)	**0.00** (43)	0.75 (24)	**0.00** (33)	**0.03** (22)
B1	**0.01** (11)	0.74 (45)	**0.00** (32)	**0.00** (27)	**0.04** (12)
B2	**0.00** (17)	0.56 (8)	1.00 (1)	0.18 (10)	**0.01** (15)
B3	0.76 (13)	**0.03** (23)	0.12 (25)	0.15 (15)	0.32 (18)
B4	0.19 (3)	0.87 (14)	1.00 (1)	0.10 (14)	**0.00** (16)

For each set of four sequences, the PHI score is given [calculated in Splitstree v4.0 (39)], significant phylogenetic inconsistencies at the 95% confidence level are highlighted in bold. The number of breakpoints for each quartet, with 95% confidence, is shown in brackets [calculated in TOPALi v2.5 (46,47)].
