# Supplementary material for: Characterization and gene expression analysis of the cir multi-gene family of plasmodium chabaudi chabaudi (AS)
Source: BMC Genomics. 2012 Mar 29;13:125. doi: 10.1186/1471-2164-13-125 (PMC3384456; doi:10.1186/1471-2164-13-125)

Supplementary data 7 (RIFIN comparison)

a)

Sub-family: A1 = PCHAS_040110, PCHAS_060060, PCHAS_000040, PCHAS_000390, PCHAS_030180; A = PCHAS_040110 to PCHAS_070060; B = PCHAS_000410 to PCHAS_001130

```
                5          15         25         35         45         55         65         75         85         95
PCHAS_040110 -------MSK ELCQGINFA- DKNVVFD--Q KSESYKFNDD IFKVYCP--- --NNNCDSNG LKIGSAFTAL LEYF-----K NIDNENSEDD KLTQYAILWF
PCHAS_060060 -------MTE ELCEAIKFA- DENVVFN--S ESQDYTFKDD IFKAFCS--L SGKGECASDE LKVGSGFMGL LEYV-----K SIDEEELDGD KLAQYAILWF
PCHAS_000040 -------MLE EVCGAINQI- NKVLSDDILT LGQHFPDDEL GYTAYCPAKE GEKGKCVTNG DRISAGFIWL LEMF--KALD DVENLKDIND QYVEYAILWL
PCHAS_000390 -------MIE V-CGVINQI- NKVLSDDILT LGQYFPDDEL GYTAYCPAKE GEKGKCVTNG DRISAGFIWL LEMF--KALD GVENLKDIND QYVEYAILWL
PCHAS_030180 -------MSK EVCEAIKFA- DEIIVFD--K KIKNYTFKND IFKVYCPQRK GPNRKCDSGG KILGAVFVAL LNFF---GSV EDYEENLKND SLSEYAILWL
PCHAS_110030 -------MAD KACTLLREV- DAYFNNE-NV NE-EKFNNSG LFTYRCP-RK GKEYICTTNN ERINTLGVYL YENL-NKISK DFKGEGNEAN RHIEIFMMWL
PCHAS_140140 -------MAI EACKILRDV- DGYFKDE-IV DE-SKFNNSG LFTYKCPKQN RRFRPCENNN ERVNTLGVYL YEKL-NGIAN KLNGEENNAN RHVEIFMMWL
PCHAS_000430 -------MDP NMCETFLEAD KIINGENGAR MKMEEIDKKS SYYEFCP--- --NKKCLTDV QRIGVMTTHV F--------- -LKGKADKNN EYGEYFLMWL
PCHAS_104200 -------MDP NMCETFLEAD KIINGENGAR MKMEDIRKSQ SFNGFCP--- --NNKCVTDE QCIGAMTMYV FSKV------ ----GADKNN EYGEYFLMWL
PCHAS_070060 -------MDS KMCEFLIKA- DKYFAGN-KV DI-KEINKNK SIKSYCH--- --NSDCKTNE DSINALAAYI IMIF------ KRSIKTNEYS HYDECLLMWL
PCHAS_000410 -------MSK GVCEEINNM- GKYIVVK-QK DSGVDIEFDN ILNDYCPKNN GRNGQCETND EKISAGFIWL LVMF--EHWC DEECPQNEKD RYVEYAILWL
PCHAS_110020 -------MDN -LCEAIDQI- DEKITLD-TN GSKQLFHYNT GSYPYCINGE GGTQECFSYE VIVSSLFIKM VNHF------ -MKGDDLKNN KLAEYAILWL
PCHAS_000100 -MRNPSYKIE DVYNDIFKI- SNYFDED-EN DGTKTKVTNK AIHNYCHDDR QEKDKCNDYY EMTSSGVIHL INNL------ -KGKNVLDYD KLAEYAILWL
PCHAS_070130 -------MAS AVCNAIKAI- DNFIVVK-EG NIGVNISFKE ILNPYCTKSL VNKEECQSYN EMVSSAFILL LKFL---NLV DVYDGDLTND RLAEYAILWL
PCHAS_120070 -------MAS GVCNAIKAI- EKFIVVK-EE DSGVYFTPNH ALKAYCNAKE GGTGVCFSYA EMVSSSVLFL LKSL---ETS YDYGDYLKND KLAEYAILWL
PCHAS_100040 -------MAS AVCGAINTI- ERLIDVK-EK DSRVYFTPNH ALKAYCVNKE RGTGVCFSYA EIVSSAVLFL LKLL---EID YHYEDDLKNA KLAEYAILWL
PCHAS_030030 -------MDN -LCKVINRL- DRKITVK-VD NLIAMIEFDK IQSKYCT-AK DEEGTCYSYE AIISSYFITL LNHF-----K SCNDNNLEND KLAQYAVLWL
PCHAS_000280 -------MNE DMCKIFEMMW EDFPDTL-GN DGNYQFKNED IYSKYCD--- SNCDNCRTDL DKINAACFVL FKIF-FEDSE SFMKNAKCNM NIAQYIIIWL
PCHAS_040040 -MPNPSYNIE DVYKEFATI- DGYFYVD-ED DGSKTKVNNQ AIHNYCDDRQ EKDKCCSGYY EMTSSGVIYL LENL------ -KKKCNLDDD KLAEYAILWL
PCHAS_001130 -------MAY GVCNAIKAI- EKLITVK-EE DSRVYFTPNH ALKAYCNVKE DGIGVCFSYA EMVSSAVLFF LKWL---ETS YDYGDYLKND KLAEYAILWL

               105        115        125        135        145        155        165        175        185        195
PCHAS_040110 NSKLSQKPDI EI-ERYTMYN ILKQYN---- ---------W FGEDGKSIEK KKDIMGIHYL YLKNLYDFLK GICETITNCN -GHSKTSECI ESAKKCSGLY
PCHAS_060060 SYKISQNKNI EL-IRGTMYG ILKQNG---- ---------W FGEHSDSIEN RKDTMEIHFE YLNRLYRFLK GICETITNCK -DSSTSSKCQ DSAKKCSGLY
PCHAS_000040 SSKNILINPD MLVSVTGIYD ILERNN---- -------PIG YKEFRNKIEK KKQLMNFGDY HMGKLYELLK EMCTLITKYD QDRSFPDSYL NHANKCANLY
PCHAS_000390 CFKNKLINPD MYVSITGIYD ILERNN---- -------PIW YNEFRNKIEK KKRLMNFDDY HMGKLYELLK EMCTLITKYN VDSSHPDAYL NYANKCANTY
PCHAS_030180 NSKIDRY-EE RLIGVDAVYD MLTRND---- ---------W FGEHYNSIKK KSDMMKLFYT YLTRLYELLK EICNTITKCN -NSPNTEECE KHAKKCVQLY
PCHAS_110030 SGKLYKLENN KSTTLEESYK KYLENN---- --------MP SFNYWNVLGS KREYKIANVW YMSRLYSLLE CICSIVIEYG KSKRN-KQIE QISQQCYQKF
PCHAS_140140 GDKSYKLDKN KTATLEDSYK DNLEKY---- --------TG NFKYWNVIDS KRVYKRANIW YMSELYKLLK SICSIVIEYN KNKSK-KKIE QISSECYQKF
PCHAS_000430 SDKLFNMYQK DKRKGENNRI TLDEAY-KKY LDKD----IG DYKYWNLLGN INGLKEANLS HMNEFYKLLK HICKTIMHHK IKPTESASLI QNSTNSSNQY
PCHAS_104200 SDKLFKMHEE GKKKGQSNIT TLDEAY-KSY LDKN----IG NNKYWDALDN VKGLKDANLS HMNEFYKLLK HICKTIMHHK NKPTESENIL QNSTNSYNQY
PCHAS_070060 SDKLYKMHLK SIGQKDTAEY MDGTTL-NQA YDNYLKNYKV GLGYWDLLDM IMGLKEANLK YMAEFYKLLN NICKIITDYN DNGSESTKLS KYSENCLNQY
PCHAS_000410 SYMLNQISNE GISTLKNFYT NHIETN-TNY TNNVTS--AS DSNYKEIIDK KINLMNANKN IIPKFYDVFK SLCSMYSELD DDEPDYTNCL KNAQNFVDEY
PCHAS_110020 CYKLNKKKEN EISNLNDFHN KYIKGNENHI DKING--AGS YNSYKNIINN KKYLNTIDIK EMSKFYVPLK SLCKLYTEYS KKKKNYTTCS QDAQDFAKHF
PCHAS_000100 SYKLKIKENP IIKKLSVFYD SYIERN-EYY NKNING---D NLTYKEIIDK KKDLMDMDIN EIFKLEAPFN ILYYLYHQIY DKNSFCSEYS DYANQFVQKF
PCHAS_070130 SYKLNQNPQK GINTLNDFYT RNIGKN-THY TSTTDD-IDV YKSYKDFIDK NNDLMTVNIK EISQFYAPFK SLCGMYIECN GKKTNHTKCL EKANEFVENF
PCHAS_120070 SYKLNKYPQK GINTLNDFYT EHIEKN-EYY NVKIT---KS SKTYKDIIDR KHDLMNIGIK EISQFYDAFK SLCDMYTELD KKNQDCENYF RKANKLVENF
PCHAS_100040 SYKLNKYSQK GITKLNDFYT QHIEKN-KYY NVEITK-SSD KKTYKDIIDR KHDLMNIGIK EISQFYDAFK SLCDMYTELD KENQDCEYYF RKANELVENF
PCHAS_030030 CHKLNKNSQN GVSNLKDIYD VYIKDNKKDI EKMSG---EA YNSCKDIINK KIYSMPIDIK EMSRLYEALK ALCKLYTECD EKKEKYTSCS RDAQDFANEF
PCHAS_000280 SYILSLKENN NISNLKDFYD LYINSNDKYN DKITG--VKD YTSYKDLIDK NNYFLSMNMK IVSKFYTSFK SLCSMYNEIE GNKSNSTKCL EEAKNFVDEY
PCHAS_040040 SYKLKIKENN MIKKLSDFYN SYIERN-EYY NKNING--GD GLTYKAIIDK KKDLMDMNIS EIFKLEAPFN ILYYLYYKIS DKHTDCEKNL NDAKKFVDKI
PCHAS_001130 SYKLNKYPQN KITTLNDFYT KHIEKN-EYY NVEIT---KS SKTYKDIIYR KHDLMNIGIK EISQFYDAFK SLCDMYNELD EENQDCENYF RKANKLVANF

               205        215        225        235        245        255        265        275        285        295
PCHAS_040110 RACILHREIC NPYCNVLTNL KNDYDKIREN ---------- ---NRDKKLP EL-------- ---TLPEGLY DCKTECYKQE EGHKARVAAL NRSSDGS---
PCHAS_060060 RACLMQREIC NPYCSVLSNL KKDYDKLREN ---------- -----NKNIP EL-------- ---ELTEGLS DCYGECSKQE KRYEEWLAAQ KHLSGGSEIV
PCHAS_000040 QNLVTESKMC DSYCYVLSTL KDAYDKFRRE ---NE-FDSD Y------QLP EF-------- ---TLPEGIK SCEKLCEKKN QELKVESSKI DVSETVTPTE
PCHAS_000390 KDLVSKVKNC DSYCNVLSTL KNAYDKFREE --KIMHDPEC -------KLP EF-------- ---NVEE-IE SCESLCKKKS QEPAIKNPIA KESEIDTPPK
PCHAS_030180 QGFVKEYGYC NPYCRILSNL KKDYENFRKN ---------- ---YTPNNLP EL-------- ---NLPNGIP SCESLCKNKE QETNTKKPIT EVSEIATPQK
PCHAS_110030 INIYKDVKEC YSYFHLLKFL KNIYDVIRND -AINE-TVAK KLNALTISLK DLTPKDWDQR FLDESDQTID LYTKNCNALH SEISKQAKKD ISKNPSTEQS
PCHAS_140140 MNINKDIKDC YSYFHLLKYL KSIYDGIRND --AIK-DADI KLNDLTISLK DLTTPEWNQR FSDESDQIID FHTQKCVELY SELAEQVKKD TPQNAPTEQS
PCHAS_000430 MLLYQNVSEC DSYLHLLDNL KKTYEKFRST --IKN-GDPN L-ASSLQTLT TI---ENTDS YFVKGFSTFD FSNSKCQSEY DDEILEKWRK DQARTKQKDN
PCHAS_104200 MLLYQNVSEC DSYLHLLDNL KKTYEKFRST --IKN-GDPN L-ASSLQTLT TI---ENTGS YLVEGFKNFD FSDRKCQSEY DDSILEKLEK TKAQRKQKDN
PCHAS_070060 RTLYINIYEC KSYLHLLNKL KGIYDDFRNS -AINK-NNSN N--NLATNLQ KLTKPDGEEM NAVRSFISYK FNKKICNSLH KKTTTQKPTN PPGLSSSSKE
PCHAS_000410 QTFLNNDIDD SSYKQILPIL SNCYDNFKKK --CNN-TQSS N----FPLLP TT-------- ---KTTQNVV EI-------- ---------- ----------
PCHAS_110020 EDLNQ-ITEN ISYREILSSL SIDYDDFKNE --CAK-NCSY C--NDIPTLS EI-------- ---------- ---------- ---------- ----------
PCHAS_000100 EDLNN-NKEN SSYNKLLSIL SDDYNNLQKK ---------- C--TNFPSLP VY-------- ---------- ---------- ---------- ----------
PCHAS_070130 EKLNG-ITGN SSYRHILYTL SADYNSLKSD --CAE-KCTD C--KDIPILS EI-------- ---------- ---------- ---------- ----------
PCHAS_120070 EKLNG-ITGN NSYRKILYTL STDYDDFKNY --FVE-KCSG Y--TNLPTLS KI-------- ---------- ---------- ---------- ----------
PCHAS_100040 EKLNG-INES SSYRKILYTL STDYDDFKNY --FVK-KCSG Y--TDLPPLS KI-------- ---------- ---------- ---------- ----------
PCHAS_030030 NKLND-ITEN NLYSQILYAL FNDYNSFKNG --CAK-NCSS C--NDVPTLS EI-------- ---------- ---------- ---------- ----------
PCHAS_000280 EKHNE-INKD SLYSQILSTL LTDYNNFKSY --FVE-KCSH C--RDISAFP VI-------- ---------- ---------- ---------- ----------
PCHAS_040040 EDLNN-NKEN SPFSQILYTL SDDYNNLQKK ---------- C--TNFPSLP VY-------- ---------- ---------- ---------- ----------
PCHAS_001130 EKLNG-INEN SSYRKILYTL STDYDDFKNY --FVE-KCSG Y--TDLPTLS KI-------- ---------- ---------- ---------- ----------

               305        315        325        335        345        355        365        375        385        395
PCHAS_040110 ---------- ----EIDTTP GISLGNKLPY IAVPLILIPI ILGISYKYLT PVWRKKTKRK -AMKKIINL- ---------- ---------- ----------
PCHAS_060060 TP-------- ----TEDSLP GSSVGNKIPY IAVPLILIPI ILGISYKYLT PVWRKKTKRK -AMKKIINL- ---------- ---------- ----------
PCHAS_000040 TILSYQTSTE LSDSREESLP KIEVGNKLPY IAVPFILIPV IFGISYKYLT LMWKKKMKSK KNVRKIINL- ---------- ---------- ----------
PCHAS_000390 ISLPVRSTTE LTDNRENTLS VNEVGNKLPY IAVPFILIPV IFGISYKYLT LMWKKKMKSK KNVRKIINL- ---------- ---------- ----------
PCHAS_030180 NSLPLLDFLN KLKGKEGESP SSGLSNKVIY IVVAFISILI ILGISYKYLP LGGVKKLKRK KKMKKIINM- ---------- ---------- ----------
PCHAS_110030 QSGSTALPPP PEPQKQDSPT PPPPKTNRPE TVITVILIPI ITLIIYKYLS RERTKKSEKK -NMKKVINL- ---------- --AYGKRKTQ IIIQSCDRTK
PCHAS_140140 QSGGQEETPT ALPQKQDSPS PSQPEKNRPE IVITVILIPI ISLIIYKYLS RERTKKSEKK -NMKKVINL- ---------- --AYGKRKTQ IIIKSCDRTK
PCHAS_000430 GAN------- ----EDNNPQ NPKVGSKQTR ILIIVILIPI TLAIMYKYLS FGRRNELKRK KNMKKVINL- ---------- --MEGKRHMQ IIIKSSSQKK
PCHAS_104200 GGNE------ ----KQNTQP ESSVGSKQTR ILIIVILIPI TLAIMYKYLS FGRRNELKRK KNMKKVINL- ---------- --MEGKRQMQ IIIKSSSQKK
PCHAS_070060 VPPPPEESHK SEKISQSEPN DSGTDQKKIV IPIIVIIISI TLAIMYKYLV FDRRKKLKRK K-MKKVTDLF GVNKTT---- ---------- ----------
PCHAS_000410 ---------- ----SEATSS SS-VASKLIP VLSIF-AISL FLGIAYKYSL FGFDKQRHRQ YLREKLKKI- ---------- ---------- ----------
PCHAS_110020 ---------- ------KTPP SS-IASKLIP VLLTC-SISF FLGIAYKYSL FGFDKRRRQQ YLRENLKK-- ---------- ---------- ----------
PCHAS_000100 ---------- --------PR SF-IKVTLIP ITFIFVAIPI FLEFAYKYSL FGFGKRSQKQ YLREKRKKA- ---------- ---------- ----------
PCHAS_070130 ---------- ------KTPP FS-ITSKLIS VLLIFVAIPI SLGIAYKYSL FGFDKRLHRQ YLREKLKKI- ---------- ---------- ----------
PCHAS_120070 ---------- ------KTLQ FS-ITSKLIP VLLAF-AIPI FLGIAYKYSL LGFDKRLHIQ YLREKRKKI- ---------- ---------- ----------
PCHAS_100040 ---------- ------KTLQ FS-ITSKLIP VLLAF-AIPI FLGIAYKYSL LGFDKRLHIQ YLREKRKKI- ---------- ---------- ----------
PCHAS_030030 ---------- ------KAPP SS-IPRKLIP VLLTF-SIPF FLGIAYKNSL FGFHKRVQRK HLRERLKK-- ---------- ---------- ----------
PCHAS_000280 ---------- ------KTTS SL-IASKLIP VLLMF-AIPI FLGIAYKYSL FGFDKRVQRQ TLRKKYK--- ---------- ---------- ----------
PCHAS 040040 ---------- --------PR SF-IKVTLIP ITFIFVAIPI FLGFAYKHSL FGFGKRSQKQ YLREKRKKI- ---------- ---------- ----------
PCHAS_001130 ---------- ------KTLQ FS-ITSKLIP VLLAF-AIPI FLGIAYKYSL LGFDKRLHIQ YLREKRKKI- ---------- ---------- ----------

               405        415        425        435        445        455        465        475        485        495
PCHAS_040110 ---------- ---------- -SDQKKA--- ---------- ---------- ---------- ---------- ---------- ---------- ----------
PCHAS_060060 ---------- ---------- -SDQKKA--- ---------- ---------- ---------- ---------- ---------- ---------- ----------
PCHAS_000040 ---------- ---------- -SDKK----- ---------- ---------- ---------- ---------- ---------- ---------- ----------
PCHAS_000390 ---------- ---------- -SDKK----- ---------- ---------- ---------- ---------- ---------- ---------- ----------
PCHAS_030180 ---------- ---------- -CVKNKIKRN LKYIIENNQR GQL------- ---------- ---------- ---------- ---------- ----------
PCHAS_110030 NLKPVIN--- ---------- ---------- ---------- ---------- -------SVD RKKDSLLNIH KLMQADSIPF INLFFLLILF SIKENTIFWN
PCHAS_140140 DLKPVIN--- ---------- ---------- ---------- ---------- -------SVD RKKDSLLNIP KLMQANSIPF INLFFLLILF SIKENTIFWN
PCHAS_000430 QTKKSIN--- ---------- ---------- ---------- ---------- -------PVY GEKSPSINIY KLIQADPVPF INLIFLVIFL FIKEKTILWK
PCHAS_104200 QTKKSIN--- ---------- ---------- ---------- ---------- -------PVY GEKSPSINIY KLMQADPVPF INLIFLLIFL FIKEKTILWK
PCHAS_070060 ---------- ---------- ---------- ---------- ---------- ---------- ---------- ---------- ---------- ----------
PCHAS_000410 ---------- ---------- --KKKMNDYI ---------- ---------- ---------- ---------- ---------- ---------- ----------
PCHAS_110020 ---------- ---------- ---------- ---------- ---------- ---------- ---------- ---------- ---------- ----------
PCHAS_000100 ---------- ---------- --KRKVYNYI LLEERDYSRN SNNY------ ---------- ---------- ---------- ---------- ----------
PCHAS_070130 ---------- ---------- --KKKMNHYI ---------- ---------- ---------- ---------- ---------- ---------- ----------
PCHAS_120070 ---------- ---------- --KRKEYNYI ---------- ---------- ---------- ---------- ---------- ---------- ----------
PCHAS_100040 ---------- ---------- --KRKEYNYI LFEESDYSRN SNNY------ ---------- ---------- ---------- ---------- ----------
PCHAS_030030 ---------- ---------- ---------- ---------- ---------- ---------- ---------- ---------- ---------- ----------
PCHAS_000280 ---------- ---------- ---------- ---------- ---------- ---------- ---------- ---------- ---------- ----------
PCHAS_040040 ---------- ---------- --KSKEYNYI LFDESDYSRN SNNY------ ---------- ---------- ---------- ---------- ----------
PCHAS_001130 ---------- ---------- --KRKEYNYI LFEESDYSRN SNNY------ ---------- ---------- ---------- ---------- ----------
```

b) CIR A1 sequences:

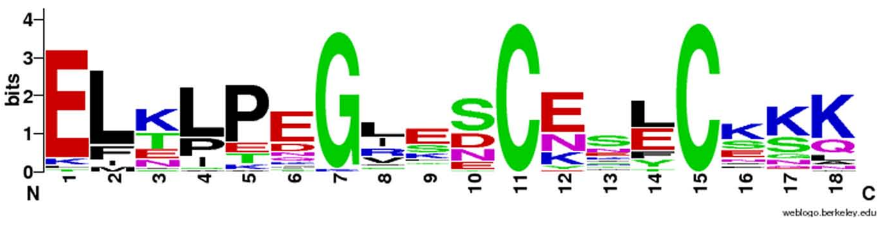

c) RIFIN A sequences:

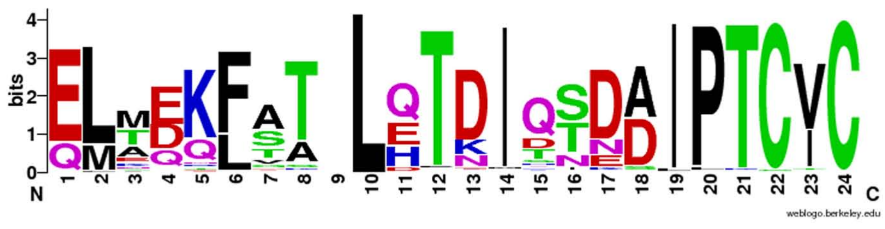

Supplement: Additional file 7 — Identification of similarities between the CIR and RIFIN repertoires. [file 1471-2164-13-125-S7.PDF]
