# Supplementary material for: Characterization and gene expression analysis of the cir multi-gene family of plasmodium chabaudi chabaudi (AS)
Source: BMC Genomics. 2012 Mar 29;13:125. doi: 10.1186/1471-2164-13-125 (PMC3384456; doi:10.1186/1471-2164-13-125)
Supplement: Additional file 8 — Raw microarray data. [file 1471-2164-13-125-S8.PDF]

Supplementary data 8 (Microarray raw data)

Oligo	NEW ID	Start / End Location	NEW identity	NEW E-value	Phase2	Phase2_TR	2h post invasion	4h post invasion
PC200021.00.0	PCHAS_146870	471 / 530	60%	5E-28	1.7296	-1.4120	0.5911	0.5735
PC200001.00.0	PCHAS_146840	261 / 320	60%	5E-28	1.7520	-1.3896	0.6704	0.5956
PC100862.00.0	PCHAS_146770	475 / 534	60%	5E-28	-3.0343	0.1073	-1.3984	-0.9519
PC106410.00.0	PCHAS_140140	291 / 350	60%	5E-28	-2.6238	0.5178	-2.1176	-1.5462
PC500048.00.0	PCHAS_140130	674 / 733	60%	5E-28	1.6916	-1.4500	0.4274	0.3261
PC200058.00.0	PCHAS_140090	101 / 160	60%	5E-28	-1.9622	1.1794	-1.9459	-1.5836
PC405624.00.0	PCHAS_140040	41 / 100	60%	5E-28	0.0458	-3.0958	1.8768	1.1364
PC107487.00.0	PCHAS_130170	884 / 943	60%	5E-28	-1.2433	1.8983	-0.7703	-0.6986
PC109216.00.0	PCHAS_130110	856 / 915	60%	5E-28	-2.2584	0.8832	-0.7380	-0.4124
PC401927.00.0	PCHAS_130100	678 / 737	60%	5E-28	-0.4534	2.6882	0.8840	0.4149
PC106237.00.0	PCHAS_130080	16 / 75	60%	5E-28	-0.5554	2.5862	0.2464	0.0922
PC401402.00.0	PCHAS_130060	599 / 658	60%	5E-28	0.9217	-2.2198	0.6355	0.4684
PC200023.00.0	PCHAS_120040	104 / 163	60%	5E-28	1.5680	-1.5736	1.1947	1.0687
PC500026.00.0	PCHAS_114730	60 / 119	60%	5E-28	-2.6999	0.4417	-0.6897	-0.4637
PC106523.00.0	PCHAS_110030	676 / 735	44%	2E-18	-2.7139	0.4277	-2.2905	-1.5792
PC500043.00.0	PCHAS_110020	39 / 98	60%	5E-28	-2.4651	0.6765	-1.1763	-0.9216
PC107858.00.0	PCHAS_090030	239 / 298	60%	5E-28	0.7681	-2.3735	-0.3757	-0.0566
PC109406.00.0	PCHAS_090010	78 / 137	60%	5E-28	2.5231	-0.6185	-0.8612	-0.3409
PC400986.00.0	PCHAS_073150	290 / 349	60%	5E-28	-2.0965	1.0451	-1.5118	-1.2980
PC100498.00.0	PCHAS_070170	2277 / 2336	60%	5E-28	1.2786	-1.8630	1.5505	1.4226
PC500040.00.0	PCHAS_070130	99 / 158	60%	5E-28	-1.7483	1.3933	-0.4338	-0.3338
PC404185.00.0	PCHAS_070060	835 / 894	60%	5E-28	1.3170	-1.8246	1.1920	1.1948
PC108118.00.0	PCHAS_060130	906 / 965	60%	5E-28	1.7038	-1.4378	0.4198	0.4682
PC101365.00.0	PCHAS_060120	343 / 402	60%	5E-28	-2.9320	0.2096	-0.4349	-0.3138
PC107506.00.0	PCHAS_060110	939 / 998	60%	5E-28	2.2481	-0.8935	-0.2728	-0.0384
PC200035.00.0	PCHAS_060060	666 / 725	60%	5E-28	1.5334	-1.6082	1.2764	1.1353
PC500027.00.0/PY02277_py	PCHAS_050020				-2.6347	0.5069	-1.6197	-1.1001
PC102124.00.0	PCHAS_042020	919 / 978	60%	5E-28	1.1259	-2.0157	0.8616	0.6588
PC400872.00.0	PCHAS_042000	1038 / 1097	60%	5E-28	1.4607	-1.6809	1.3891	1.2194
PC404354.00.0	PCHAS_041990	1160 / 1219	60%	5E-28	-2.5901	0.5515	-2.8469	-2.0220
PC105627.00.0	PCHAS_041960	1222 / 1281	60%	5E-28	1.6353	-1.5063	0.5895	0.6193
PC100220.00.0	PCHAS_030190	1594 / 1653	60%	5E-28	1.6496	-1.4920	0.7857	0.7479
PC200054.00.0	PCHAS_030180	458 / 517	60%	5E-28	2.0251	-1.1165	0.0134	0.1135
PC101826.00.0/PC107236.00.0	PCHAS_030160				1.3963	-1.7453	0.5774	0.4826
PC200059.00.0	PCHAS_011500	22 / 81	60%	5E-28	-1.6282	1.5134	-1.3307	-1.0144
PC200022.00.0	PCHAS_011450	297 / 356	39%	2E-15	1.7823	-1.3593	0.3860	0.5047
PC400436.00.0	PCHAS_010120	715 / 774	60%	5E-28	2.0098	-1.1318	0.1427	0.2103
PC106057.00.0	PCHAS_010040	455 / 514	60%	5E-28	0.5237	-2.6179	0.6491	0.4712
PC500050.00.0	PCHAS_010030	40 / 99	60%	5E-28	1.2604	-1.8812	1.3083	1.0955
PY06489_py	PCHAS_001130	875 / 923	34%	2E-12	-3.1354	0.0062	-0.7665	-0.4090
PC500011.00.0	PCHAS_001090	502 / 561	60%	5E-28	-2.6215	0.5201	-0.5969	-0.4581
PC500002.00.0	PCHAS_001010	640 / 699	60%	5E-28	0.4402	-2.7014	0.9717	0.6266
PC500046.00.0/PC200004.00.0	PCHAS_000950				2.0092	-1.1324	0.6749	0.6812
PC500045.00.0	PCHAS_000730	49 / 108	60%	5E-28	2.3010	-0.8406	-0.1243	0.0154
PC500020.00.0	PCHAS_000390	635 / 694	60%	5E-28	-2.8310	0.3106	-1.5264	-0.8881
PC200002.00.0	PCHAS_000150	667 / 726	60%	5E-28	0.0699	-3.0717	0.5935	0.3653
PC500008.00.1	PCHAS_000120	641 / 700	60%	5E-28	0.8806	-2.2610	1.0584	0.7907
PC200036.00.0	PCHAS_000110	477 / 536	60%	5E-28	-2.3807	0.7609	-1.1350	-0.8462
PC200043.00.0	PCHAS_000040	767 / 826	60%	5E-28	-0.6603	2.4813	0.0844	0.0073

Supplementary data 8 (Microarray raw data)

6h post invasion	8h post invasion	10h post invasion	12h post invasion	14h post invasion	16h post invasion	18h post invasion	20h post invasion	22h post invasion	24h post invasion	Max-min
0.5404	0.5034	0.4464	0.3410	0.1881	-0.0498	-0.3417	-0.6271	-0.9280	-1.2373	1.8285
0.5182	0.4377	0.4116	0.3698	0.2516	-0.0071	-0.3418	-0.6425	-0.9686	-1.2949	1.9653
-0.4922	-0.0500	0.4146	0.8358	1.0159	0.8899	0.5434	0.1519	-0.2627	-0.6964	2.4144
-0.9716	-0.4013	0.1914	0.6876	0.9918	1.1196	0.9804	0.6747	0.3631	0.0281	3.2372
0.2353	0.1493	0.1162	0.0943	0.1017	0.0489	-0.0962	-0.2798	-0.4661	-0.6572	1.0846
-1.2166	-0.8568	-0.4263	0.0090	0.3388	0.6491	0.9265	1.1442	1.3679	1.5934	3.5393
0.4347	-0.2405	-0.6566	-0.8803	-0.8828	-0.7331	-0.4737	-0.1640	0.1428	0.4402	2.7596
-0.6331	-0.5622	-0.5553	-0.5698	-0.4644	-0.1542	0.3308	0.8279	1.3563	1.8929	2.6632
-0.1135	0.1638	0.1418	0.0488	-0.0258	-0.0924	0.0080	0.1735	0.3364	0.5097	1.2477
-0.0302	-0.4641	-0.6651	-0.7479	-0.7123	-0.4572	-0.0959	0.2533	0.6222	0.9983	1.7461
-0.0517	-0.1984	-0.2650	-0.2957	-0.3022	-0.2052	-0.0465	0.1408	0.3380	0.5471	0.8493
0.3117	0.1522	0.0100	-0.0964	-0.1607	-0.1766	-0.1984	-0.2612	-0.3147	-0.3699	1.0053
0.9369	0.8045	0.6361	0.4249	0.1212	-0.2544	-0.6360	-1.0338	-1.4338	-1.8290	3.0238
-0.2510	-0.0444	0.1072	0.2161	0.2676	0.2783	0.2416	0.1800	0.1134	0.0446	0.9680
-0.8849	-0.2033	0.3668	0.7909	0.9747	0.9898	0.8285	0.5936	0.3360	0.0775	3.2803
-0.6338	-0.3385	-0.0141	0.3469	0.5864	0.5702	0.4939	0.4290	0.3617	0.2960	1.7627
0.2198	0.4446	0.2207	-0.0869	-0.2977	-0.3746	-0.2673	-0.0355	0.1988	0.4104	0.8204
0.1331	0.5664	0.6314	0.5622	0.3878	0.1231	-0.0674	-0.2102	-0.3766	-0.5479	1.4926
-1.0554	-0.7957	-0.3617	0.1141	0.4905	0.7730	0.9091	0.9106	0.9139	0.9114	2.4257
1.2619	1.0795	0.6927	0.1920	-0.3448	-0.7520	-1.0182	-1.1892	-1.3658	-1.5291	3.0796
-0.2431	-0.1495	-0.0617	-0.0153	-0.0064	0.0235	0.1278	0.2392	0.3582	0.4949	0.9287
1.1485	1.0734	0.6926	0.1976	-0.2526	-0.6755	-0.9965	-1.0881	-1.1907	-1.2956	2.4905
0.5143	0.5516	0.4279	0.2671	0.1154	-0.1043	-0.3136	-0.5426	-0.7820	-1.0219	1.5735
-0.1805	-0.0524	0.0762	0.2260	0.3486	0.3750	0.2401	0.0796	-0.0856	-0.2784	0.8099
0.1871	0.4004	0.3794	0.2853	0.1929	0.0509	-0.0717	-0.2188	-0.3694	-0.5250	0.9254
0.9794	0.8153	0.6329	0.3985	0.1033	-0.2876	-0.6946	-1.0654	-1.4539	-1.8395	3.1159
-0.6123	-0.1680	0.1671	0.4683	0.6016	0.6573	0.6751	0.5026	0.3155	0.1127	2.2958
0.4578	0.2558	0.0983	-0.0169	-0.1132	-0.2188	-0.3674	-0.4421	-0.5369	-0.6370	1.4986
1.0378	0.8388	0.5677	0.3148	0.0410	-0.3498	-0.7558	-1.0790	-1.4291	-1.7949	3.1840
-1.2037	-0.3980	0.2805	0.8179	1.1324	1.2206	1.1002	0.8767	0.6414	0.4009	4.0675
0.6283	0.6210	0.4780	0.3056	0.1209	-0.1488	-0.4577	-0.6758	-0.9127	-1.1677	1.7960
0.6975	0.6482	0.5387	0.3604	0.1354	-0.1095	-0.4643	-0.7879	-1.1130	-1.4391	2.2248
0.1964	0.2735	0.2863	0.2437	0.1602	0.0151	-0.1373	-0.2546	-0.3879	-0.5223	0.8085
0.3796	0.2753	0.1780	0.0830	-0.0171	-0.1508	-0.2907	-0.3914	-0.5051	-0.6206	1.1980
-0.7070	-0.4127	-0.3013	-0.2331	-0.1242	0.0766	0.3706	0.7882	1.2238	1.6644	2.9951
0.5986	0.6682	0.5338	0.3836	0.2166	-0.0715	-0.3926	-0.6516	-0.9353	-1.2406	1.9088
0.2591	0.2965	0.2875	0.2696	0.2001	0.0599	-0.1113	-0.3126	-0.5372	-0.7646	1.0611
0.2934	0.1206	-0.0449	-0.2128	-0.3119	-0.3311	-0.2891	-0.2076	-0.1182	-0.0187	0.9802
0.8826	0.6578	0.3851	0.1111	-0.1645	-0.4572	-0.7246	-0.8766	-1.0310	-1.1864	2.4947
-0.0821	0.2079	0.2697	0.2611	0.1094	-0.0193	0.0393	0.0802	0.1258	0.1834	1.0362
-0.3156	-0.1745	0.0390	0.2413	0.3323	0.3585	0.3117	0.2049	0.0885	-0.0311	0.9554
0.2950	-0.0302	-0.1664	-0.2338	-0.3020	-0.3163	-0.2654	-0.2293	-0.1991	-0.1509	1.2880
0.6725	0.6549	0.5491	0.3869	0.1603	-0.1504	-0.4970	-0.7586	-1.0433	-1.3304	2.4849
0.1490	0.2810	0.3598	0.3598	0.2962	0.1434	-0.0803	-0.2722	-0.4677	-0.6602	1.0200
-0.3244	0.2127	0.4453	0.4377	0.2559	0.1132	0.1576	0.2673	0.3704	0.4788	2.0052
0.1473	-0.0629	-0.1797	-0.2682	-0.3468	-0.3343	-0.2348	-0.0696	0.1024	0.2879	0.9403
0.5220	0.2529	0.0382	-0.1391	-0.2728	-0.3729	-0.4643	-0.4593	-0.4701	-0.4839	1.5423
-0.5571	-0.2746	0.0171	0.2393	0.3744	0.4446	0.4883	0.4561	0.4181	0.3750	1.6233
-0.0740	-0.1317	-0.3254	-0.4599	-0.4128	-0.2272	0.0007	0.2492	0.5118	0.7777	1.2376
