# Supplementary material for: Characterization and gene expression analysis of the cir multi-gene family of plasmodium chabaudi chabaudi (AS)
Source: BMC Genomics. 2012 Mar 29;13:125. doi: 10.1186/1471-2164-13-125 (PMC3384456; doi:10.1186/1471-2164-13-125)
Supplement: Additional file 9 — Raw RNA sequencing data. [file 1471-2164-13-125-S9.PDF]

Supplementary data 9 (RNAseq raw data)

ID	Sub-family	BALB/c 1	BALB/c 2	BALB/c 3	BALB/c 4	C57BL/6 1	C57BL/6 2
PCHAS_110030	A	412.24620	595.51767	552.26282	563.52188	1234.66081	1123.03175
PCHAS_104200	A	5.83554	17.10556	4.69527	17.53656	208.49932	205.97652
PCHAS_000720	B	0.00000	0.00000	4.57162	12.19624	56.70908	30.29978
PCHAS_110020	B	26.29787	6.20366	12.48742	6.80164	51.78661	20.90476
PCHAS_070130	B	132.85097	16.49163	47.30460	33.47619	44.59240	26.08151
PCHAS_000770	A	0.00000	0.00000	0.00000	0.00000	44.23141	29.87805
PCHAS_000100	B	10.57163	2.81712	0.00000	3.97114	39.73252	32.55524
PCHAS_030270	A	27.87233	5.82248	14.87557	17.04663	37.30868	34.73326
PCHAS_030040	B	6.81015	0.00000	0.00000	0.00000	34.84499	34.13682
PCHAS_001090	B	25.93297	5.30734	9.51474	1.87037	34.24773	22.22144
PCHAS_011490	A	0.00000	15.30116	7.69997	6.59059	32.12656	41.86216
PCHAS_000430	A	45.72518	20.72378	13.25325	20.28693	29.53350	24.38107
PCHAS_000120	B	0.00000	0.00000	13.70894	7.44758	29.23944	16.11602
PCHAS_140020	B	7.95033	38.13476	11.99405	11.19927	27.92240	0.00000
PCHAS_140140	A	23.69719	28.24443	46.15018	40.30017	27.85895	17.55852
PCHAS_000310	B	14.97390	0.00000	0.00000	0.00000	27.55360	19.36101
PCHAS_114720	A	20.06840	9.48655	9.68821	12.58605	24.14627	20.95023
PCHAS_060130	A	0.00000	3.43026	0.00000	3.62659	23.44639	0.00000
PCHAS_000140	B	0.00000	7.41285	12.12365	3.48316	21.76724	18.40843
PCHAS_073180	B	23.57855	0.00000	0.00000	0.00000	20.86663	0.00000
PCHAS_073200	B	6.47086	0.00000	7.09971	7.07119	20.29183	23.79832
PCHAS_000340	B	6.53232	6.96291	0.00000	4.90762	19.62502	0.00000
PCHAS_073190	B	9.10464	0.00000	0.00000	2.41417	18.99535	17.93272
PCHAS_000300	B	0.00000	15.67148	0.00000	3.48809	17.54383	0.00000
PCHAS_073130	A	0.00000	0.00000	0.00000	0.00000	17.49328	17.87914
PCHAS_070020	B	0.00000	0.00000	0.00000	0.00000	17.16196	0.00000
PCHAS_104260	B	27.58396	15.92620	19.88216	17.26946	16.94313	0.00000
PCHAS_000090	B	0.00000	6.74155	0.00000	0.00000	16.57789	0.00000
PCHAS_001130	B	57.07689	6.38812	0.00000	3.85928	0.00000	0.00000
PCHAS_001040	B	24.94467	3.62577	7.52642	4.25920	0.00000	0.00000
PCHAS_100040	B	20.55790	0.00000	0.00000	0.00000	0.00000	0.00000
PCHAS_011450	A	17.67562	0.00000	0.00000	0.00000	0.00000	0.00000
PCHAS_040060	A	17.35116	4.43878	5.86351	3.12855	0.00000	0.00000
PCHAS_011520	B	15.69992	42.48070	0.00000	2.72194	0.00000	0.00000
PCHAS_040110	A	14.55461	0.00000	0.00000	0.00000	0.00000	0.00000
PCHAS_000420	B	10.90404	0.00000	4.53796	0.00000	0.00000	0.00000
PCHAS_070060	A	8.74125	11.08456	9.82224	4.75552	0.00000	0.00000
PCHAS_001100	A	6.54363	0.00000	0.00000	0.00000	0.00000	0.00000
PCHAS_040020	B	6.30642	0.00000	0.00000	7.10686	0.00000	0.00000
PCHAS_000280	B	6.28830	0.00000	0.00000	7.08643	0.00000	0.00000
PCHAS_000020	B	5.85795	0.00000	0.00000	4.59233	0.00000	0.00000
PCHAS_070040	A	5.10973	9.44069	7.40032	8.70140	0.00000	0.00000
PCHAS_000030	B	0.00000	0.00000	0.00000	3.18615	0.00000	0.00000
PCHAS_000040	A	0.00000	0.00000	0.00000	0.00000	0.00000	0.00000
PCHAS_000060	B	0.00000	2.71862	0.00000	2.87422	0.00000	0.00000
PCHAS_000070	B	0.00000	0.00000	6.97318	0.00000	0.00000	0.00000
PCHAS_000110	B	0.00000	23.63158	3.93488	0.00000	0.00000	0.00000
PCHAS_000130	B	0.00000	5.22664	0.00000	0.00000	0.00000	0.00000
PCHAS_000150	B	0.00000	3.74611	0.00000	0.00000	0.00000	0.00000
PCHAS_000170	A	0.00000	2.96464	0.00000	3.34327	0.00000	0.00000
PCHAS_000180	A	0.00000	0.00000	0.00000	0.00000	0.00000	0.00000
PCHAS_000220	A	0.00000	0.00000	0.00000	0.00000	0.00000	0.00000
PCHAS_000260	A	0.00000	0.00000	0.00000	19.57206	0.00000	0.00000
PCHAS_000290	B	0.00000	0.00000	0.00000	0.00000	0.00000	0.00000
PCHAS_000320	B	0.00000	3.00590	11.34489	2.11862	0.00000	0.00000
PCHAS_000350	B	0.00000	0.00000	0.00000	0.00000	0.00000	0.00000

PCHAS_000360	B	0.00000	0.00000	0.00000	0.00000	0.00000	0.00000
PCHAS_000390	A	0.00000	0.00000	0.00000	0.00000	0.00000	0.00000
PCHAS_000400	B	0.00000	0.00000	0.00000	0.00000	0.00000	0.00000
PCHAS_000410	B	0.00000	0.00000	9.27586	0.00000	0.00000	0.00000
PCHAS_000470	A	0.00000	0.00000	0.00000	0.00000	0.00000	0.00000
PCHAS_000490	B	0.00000	0.00000	0.00000	1.91615	0.00000	0.00000
PCHAS_000500	A	0.00000	0.00000	14.34936	0.00000	0.00000	0.00000
PCHAS_000560	B	0.00000	11.98988	0.00000	0.00000	0.00000	0.00000
PCHAS_000570	B	0.00000	0.00000	0.00000	0.00000	0.00000	0.00000
PCHAS_000580	B	0.00000	0.00000	4.32049	0.00000	0.00000	0.00000
PCHAS_000660	A	0.00000	0.00000	0.00000	0.00000	0.00000	0.00000
PCHAS_000680	B	0.00000	0.00000	0.00000	1.96422	0.00000	0.00000
PCHAS_000730	B	0.00000	0.00000	0.00000	0.00000	0.00000	0.00000
PCHAS_000740	B	0.00000	0.00000	0.00000	0.00000	0.00000	0.00000
PCHAS_000750	B	0.00000	0.00000	0.00000	0.00000	0.00000	0.00000
PCHAS_001010	B	0.00000	0.00000	0.00000	0.00000	0.00000	0.00000
PCHAS_001050	A	0.00000	0.00000	0.00000	0.00000	0.00000	0.00000
PCHAS_001060	A	0.00000	5.06453	0.00000	3.05965	0.00000	0.00000
PCHAS_001110	B	0.00000	0.00000	0.00000	1.89625	0.00000	0.00000
PCHAS_001120	B	0.00000	0.00000	5.22643	4.33787	0.00000	0.00000
PCHAS_010020	B	0.00000	0.00000	4.43136	0.00000	0.00000	0.00000
PCHAS_010030	B	0.00000	0.00000	0.00000	0.00000	0.00000	0.00000
PCHAS_010040	A	0.00000	0.00000	0.00000	0.00000	0.00000	0.00000
PCHAS_011330	A	0.00000	0.00000	0.00000	0.00000	0.00000	0.00000
PCHAS_011480	A	0.00000	0.00000	0.00000	1.81931	0.00000	0.00000
PCHAS_011500	A	0.00000	0.00000	0.00000	0.00000	0.00000	0.00000
PCHAS_011510	B	0.00000	0.00000	0.00000	0.00000	0.00000	0.00000
PCHAS_011530	B	0.00000	0.00000	0.00000	0.00000	0.00000	0.00000
PCHAS_030020	B	0.00000	0.00000	0.00000	0.00000	0.00000	0.00000
PCHAS_030060	B	0.00000	0.00000	0.00000	0.00000	0.00000	0.00000
PCHAS_030070	A	0.00000	0.00000	0.00000	0.00000	0.00000	0.00000
PCHAS_030080	B	0.00000	0.00000	0.00000	0.00000	0.00000	0.00000
PCHAS_030090	A	0.00000	0.00000	0.00000	0.00000	0.00000	0.00000
PCHAS_030110	A	0.00000	0.00000	0.00000	0.00000	0.00000	0.00000
PCHAS_030120	A	0.00000	0.00000	0.00000	0.00000	0.00000	0.00000
PCHAS_030140	A	0.00000	0.00000	0.00000	5.94594	0.00000	0.00000
PCHAS_030180	A	0.00000	0.00000	0.00000	0.00000	0.00000	0.00000
PCHAS_030190	A	0.00000	0.00000	0.00000	0.00000	0.00000	0.00000
PCHAS_030210	A	0.00000	10.59059	0.00000	4.66530	0.00000	0.00000
PCHAS_040030	B	0.00000	0.00000	0.00000	0.00000	0.00000	0.00000
PCHAS_040040	B	0.00000	0.00000	0.00000	4.71526	0.00000	0.00000
PCHAS_040050	A	0.00000	0.00000	0.00000	0.00000	0.00000	0.00000
PCHAS_041950	A	0.00000	0.00000	0.00000	0.00000	0.00000	0.00000
PCHAS_041970	A	0.00000	0.00000	7.88816	0.00000	0.00000	0.00000
PCHAS_041980	A	0.00000	0.00000	0.00000	0.00000	0.00000	0.00000
PCHAS_041990	A	0.00000	0.00000	0.00000	0.00000	0.00000	0.00000
PCHAS_042020	A	0.00000	0.00000	0.00000	0.00000	0.00000	0.00000
PCHAS_042030	A	0.00000	0.00000	0.00000	0.00000	0.00000	0.00000
PCHAS_042070	A	0.00000	0.00000	4.89833	4.57374	0.00000	0.00000
PCHAS_050020	B	0.00000	0.00000	0.00000	0.00000	0.00000	0.00000
PCHAS_050040	A	0.00000	0.00000	0.00000	0.00000	0.00000	0.00000
PCHAS_050060	B	0.00000	6.11690	0.00000	0.00000	0.00000	0.00000
PCHAS_050070	A	0.00000	0.00000	0.00000	0.00000	0.00000	0.00000
PCHAS_060020	B	0.00000	0.00000	0.00000	0.00000	0.00000	0.00000
PCHAS_060030	A	0.00000	0.00000	0.00000	0.00000	0.00000	0.00000
PCHAS_060050	A	0.00000	0.00000	0.00000	0.00000	0.00000	0.00000
PCHAS_060060	A	0.00000	0.00000	0.00000	0.00000	0.00000	0.00000
PCHAS_060070	A	0.00000	5.01475	5.67802	13.80143	0.00000	0.00000

PCHAS_060090	A	0.00000	0.00000	0.00000	0.00000	0.00000	0.00000
PCHAS_060110	A	0.00000	0.00000	0.00000	0.00000	0.00000	0.00000
PCHAS_060140	A	0.00000	0.00000	0.00000	0.00000	0.00000	0.00000
PCHAS_060160	A	0.00000	0.00000	0.00000	0.00000	0.00000	0.00000
PCHAS_070030	B	0.00000	0.00000	0.00000	3.40149	0.00000	0.00000
PCHAS_070050	B	0.00000	0.00000	0.00000	2.41772	0.00000	0.00000
PCHAS_070070	A	0.00000	0.00000	0.00000	0.00000	0.00000	0.00000
PCHAS_070100	A	0.00000	0.00000	0.00000	0.00000	0.00000	0.00000
PCHAS_070160	B	0.00000	0.00000	8.53065	0.00000	0.00000	0.00000
PCHAS_070170	A	0.00000	0.00000	0.00000	2.61459	0.00000	0.00000
PCHAS_073150	A	0.00000	0.00000	0.00000	0.00000	0.00000	0.00000
PCHAS_073160	B	0.00000	0.00000	0.00000	0.00000	0.00000	0.00000
PCHAS_083720	A	0.00000	6.48839	0.00000	15.43443	0.00000	18.70962
PCHAS_083760	A	0.00000	5.25355	0.00000	2.46855	0.00000	0.00000
PCHAS_090010	A	0.00000	0.00000	0.00000	0.00000	0.00000	0.00000
PCHAS_100030	B	0.00000	0.00000	0.00000	0.00000	0.00000	0.00000
PCHAS_100060	A	0.00000	0.00000	0.00000	5.52225	0.00000	0.00000
PCHAS_104230	B	0.00000	6.02214	0.00000	5.65939	0.00000	0.00000
PCHAS_104250	B	0.00000	4.24105	5.20215	2.98919	0.00000	0.00000
PCHAS_114600	A	0.00000	6.30965	0.00000	1.87250	0.00000	0.00000
PCHAS_114640	A	0.00000	3.41020	0.00000	0.00000	0.00000	0.00000
PCHAS_114700	A	0.00000	0.00000	0.00000	0.00000	0.00000	0.00000
PCHAS_114730	B	0.00000	0.00000	0.00000	0.00000	0.00000	0.00000
PCHAS_114740	B	0.00000	0.00000	0.00000	0.00000	0.00000	0.00000
PCHAS_114750	B	0.00000	0.00000	0.00000	0.00000	0.00000	0.00000
PCHAS_120020	B	0.00000	0.00000	0.00000	0.00000	0.00000	0.00000
PCHAS_120030	A	0.00000	0.00000	0.00000	0.00000	0.00000	0.00000
PCHAS_120040	B	0.00000	0.00000	0.00000	0.00000	0.00000	0.00000
PCHAS_120050	B	0.00000	24.85532	0.00000	5.39034	0.00000	0.00000
PCHAS_120060	B	0.00000	0.00000	0.00000	0.00000	0.00000	0.00000
PCHAS_120070	B	0.00000	0.00000	15.75292	5.04311	0.00000	0.00000
PCHAS_130020	B	0.00000	0.00000	0.00000	0.00000	0.00000	0.00000
PCHAS_130030	A	0.00000	0.00000	0.00000	0.00000	0.00000	0.00000
PCHAS_130050	A	0.00000	0.00000	0.00000	0.00000	0.00000	0.00000
PCHAS_130060	A	0.00000	0.00000	0.00000	3.35360	0.00000	0.00000
PCHAS_130070	A	0.00000	0.00000	0.00000	0.00000	0.00000	0.00000
PCHAS_130080	A	0.00000	0.00000	0.00000	0.00000	0.00000	0.00000
PCHAS_130100	A	0.00000	0.00000	0.00000	5.41355	0.00000	0.00000
PCHAS_130120	A	0.00000	2.76897	0.00000	2.73228	0.00000	0.00000
PCHAS_130170	A	0.00000	2.99707	0.00000	0.00000	0.00000	0.00000
PCHAS_130220	A	0.00000	0.00000	0.00000	0.00000	0.00000	0.00000
PCHAS_130280	A	0.00000	0.00000	0.00000	2.53364	0.00000	0.00000
PCHAS_137030	A	0.00000	0.00000	0.00000	1.89407	0.00000	0.00000
PCHAS_137110	A	0.00000	0.00000	0.00000	0.00000	0.00000	0.00000
PCHAS_140030	B	0.00000	24.13886	0.00000	1.79091	0.00000	0.00000
PCHAS_140040	B	0.00000	0.00000	0.00000	0.00000	0.00000	0.00000
PCHAS_140070	A	0.00000	0.00000	0.00000	0.00000	0.00000	0.00000
PCHAS_140090	B	0.00000	0.00000	0.00000	8.59261	0.00000	0.00000
PCHAS_140130	B	0.00000	0.00000	0.00000	0.00000	0.00000	0.00000
PCHAS_146790	A	0.00000	0.00000	0.00000	3.03985	0.00000	0.00000
PCHAS_146850	A	0.00000	0.00000	0.00000	0.00000	0.00000	0.00000
PCHAS_146860	A	0.00000	0.00000	0.00000	0.00000	0.00000	0.00000
PCHAS_146870	A	0.00000	0.00000	0.00000	6.37230	0.00000	0.00000
