# Supplementary material for: Characterization and gene expression analysis of the cir multi-gene family of plasmodium chabaudi chabaudi (AS)
Source: BMC Genomics. 2012 Mar 29;13:125. doi: 10.1186/1471-2164-13-125 (PMC3384456; doi:10.1186/1471-2164-13-125)
Supplement: Additional file 11 — Legends to Additional Files 1-10. [file 1471-2164-13-125-S11.PDF]

SUPPLEMENTARY DATA

1) Alignment of 183 CIR sequences

CIR amino acid sequences were aligned using Muscle (38). Sequences which aligned poorly were removed from the alignment, leaving 183 CIRs. This alignment is presented in FASTA format, a), underneath which, the CIRs excluded from the alignment are tabulated, b).

2) CIR alignment conservation

Sequence similarity within the CIR alignment was calculated using Plotcon (http://emboss.bioinformatics.nl/cgi-bin/emboss/plotcon). Sequence similarity is plotted against relative residue position, for each position within the alignment.

3) CIR network showing bootstrap values

The CIR network presented in Figure 1 is shown here including the CIR identifiers and support values generated from 500 bootstrap replicates in Splitstree (39). The dominant cir transcripts identified in Figure 5 by RNA sequencing, are indicated by black dots.

4) Maximum likelihood tree of CIR sequences

To validate the network of CIR sequences presented in Figure 2 and supplementary data 2, a Maximum Likelihood tree was created in PhyML (42). In order to correctly identify the relationships between CIR sub-families, three YIR and three BIR sequences were added to the CIR alignment. The resulting tree was rooted using the YIR and BIR sequences as an out-group. The

PCHAS_ species identifier was not shown for the CIR sequences.

5) Sub-families identified from the alignment of 183 CIRs

Members of each CIR sub-family identified in Figure 2 are tabulated.

6) Detection of phylogenetic incompatibilities between cir genes

An example analysis between members of cir sub-family B1 is shown, a) [which is further described, b)]. A NeighborNet network (40) generated using aligned amino acid sequences from CIR subfamily B1, i). Sequence identities of the aligned DNA sequences for CIR sub-family B1 members, generated in Plotcon (http://emboss.bioinformatics.nl/cgi-bin/emboss/plotcon), ii). An example TOPALi profile (46,47), iii), showing the three possible phylogenetic arrangements of sequences PCHAS_000100, PCHAS_000310, PCHAS_120060 and PCHAS_040040. The phylogenetic profile indicates how the relatedness of the four analyzed sequences changes along their length. An example phylogenetic reconstruction is found to the right of the profile window, displaying the topology of that relationship. A reconstruction of PCHAS_000100, iv), according to the phylogenetic profile shown above. Each block represents the cir sequence which displays closest homology with that region of PCHAS_000100, such that effectively this gene is a mosaic of the other three. This was performed for 5 quartets selected at random from each cir clade. Results from all cir clades are tabulated, c).

7) Identification of similarities between the CIR and RIFIN repertoires.

a) Ten representative CIR sequences from subfamilies A or B are shown,

from the alignment of 183 CIRs. Between the conserved DY and YK residues at the N terminus and C terminus of this region, there are striking differences between these sets of CIRs. The majority (87 and 96 sequences) of members of either group of CIRs were aligned separately, and a weblogo created of the most conserved amino acids found in this region of CIR subfamilies A and B. A striking motif was observed within subfamily A sequences, particularly clade A1, identified by a red line. The subfamily A motif was enlarged, b), and compared to the insert present in the RIFIN subfamily A (16,17), c). Weblogos were created of each insert (37). Polar residues are shown in green, basic residues in blue, acidic residues in red and hydrophobic residues in black.

8) Raw microarray data

The probe (oligo) name is given for each expressed cir gene, along with its current gene ID and the E-value for each probe matching that cir gene. The position of each probe binding to their respective gene is indicated in the start and end location columns. Expression levels at each of the 12 time-points and the difference between maximum and minimum levels of detection are also given. Average readings for each time point were taken only for blue rows of genes which are detected by two different oligos but bind to different regions of the gene.

9) Raw RNA sequencing data

The RPKM values for each cir transcript are shown for each of four BALB/c and two C57/BL6 experiments. These values were set to zero where they fell below the cut-off for that experiment.

10) cir gene expression threshold of detection determination

The threshold of detection above which a gene was deemed to be expressed was determined for each RNAseq experiment. To do this we picked the RPKM values below which 90% of intronic sequences were expressed. We assume that intronic sequences should not be expressed and therefore are a reliable region to quantify the extent of mapping due to random chance. a) BALB/c 1, b) BALB/c 2, c) BALB/c 3, d) BALB/c 4, e) C57BL/6 1, f) C57BL/6 2.
